# Large-scale compressive microscopy via diffractive multiplexing across a sensor array

**DOI:** 10.1038/s41566-026-01974-4

**Published:** 2026-07-28

**Authors:** Kevin C. Zhou, Chaoying Gu, Muneki Ikeda, Tina M. Hayward, Nicholas Antipa, Rajesh Menon, Roarke Horstmeyer, Saul Kato, Laura Waller

**Affiliations:** 1 Department of Electrical Engineering and Computer Sciences, University of California, Berkeley, CA USA; 2 Department of Biomedical Engineering, University of Michigan, Ann Arbor, MI USA; 3 Department of Neurology, University of California, San Francisco, CA USA; 4 Department of Electrical and Computer Engineering, University of Utah, Salt Lake City, UT USA; 5 Department of Electrical and Computer Engineering, University of California, San Diego, CA USA; 6 Department of Biomedical Engineering, Duke University, Durham, NC USA; 7 Ramona Optics, Durham, NC USA

**Keywords:** Microscopy, Imaging and sensing, Optical sensors

## Abstract

Microscopes face a trade-off between spatial resolution, field of view and frame rate—improving one of these properties typically requires sacrificing others, owing to the limited spatiotemporal throughput of the sensor. To overcome this, we propose a new microscope that achieves snapshot gigapixel-scale imaging with a sensor array and a diffractive optical element. We improve spatiotemporal throughput in two ways. First, we capture data with an array of 48 sensors, resulting in 48× more pixels than a single sensor. Second, we use point spread function engineering and compressive sensing algorithms to fill in the missing information from the gaps between the individual sensors in the array, further increasing the spatiotemporal throughput of the system by an additional >5.4×. The array of sensors is modelled as a single large-format ‘super sensor’, with erasures corresponding to the gap areas between sensors. The sensor array is placed at the output of a (nearly) 4*f* imaging system, with a diffractive optical element in the Fourier plane that generates a distributed multi-spot point spread function. This enables encoding of information from the entire super-sensor area, including the gaps. We then perform a large-scale regularized reconstruction using a direct fully shift-variant convolutional forward model, assuming that the object is sparse in some domain. Our microscope can achieve ~3 μm resolution over >5.2 cm^2^ field of view at up to 120 fps, culminating in an effective total spatiotemporal throughput of 25.2 billion pixels per second. We demonstrate the versatility of our microscope in two different modes: structural imaging via dark-field contrast and functional fluorescence imaging of calcium dynamics across dozens of freely moving *Caenorhabditis elegans*.

## Abstract (web summary)

By combining an array of 48 sensors with a diffractive mask and compressive sensing, researchers developed a 25 gigapixel-per-second computational microscope that can image at micrometre resolution across multi-centimetre areas at 120 frames per second.

## Main

Optical engineers design microscopes within the constraints of spatiotemporal throughput limits set by the pixel count and frame rate of the sensor. Spatiotemporal throughput is defined as the space-bandwidth product (SBP)—the number of spatially resolvable points—per unit time. Strategies that expand the SBP^1^ typically sacrifice frame rate. For example, physically scanning the sample^2^ or using scanning mirrors to build up a larger composite field of view (FOV)^3,4^ limits acquisition times. Other techniques keep a constant FOV and improve resolution by capturing multiple images, then computationally combining them (for example, Fourier ptychography^5–7^, single-molecule localization microscopy^8^ and structured illumination microscopy^9^). These methods increase SBP and often achieve faster acquisition times than sample scanning since they do not require any moving parts. However, sequential measurements may not be appropriate for imaging dynamic samples, since the sample is assumed to remain static during the sequence.

To achieve high SBP microscopy with faster acquisition, multiplexing and compressive sensing can enable fewer measurements, for example, by illuminating the sample with multiple light-emitting diodes (LEDs) simultaneously in Fourier ptychography^10,11^ or overlapping multiple FOVs^12–15^, which are demixed computationally. More recently, computational approaches can model and correct for sample motion during multi-shot acquisition^16,17^. However, multi-camera array architectures have proven most useful for truly single-shot large spatiotemporal throughput imaging since they directly increase the spatiotemporal throughput of the sensor^18–20^.

**Fig. 1:**
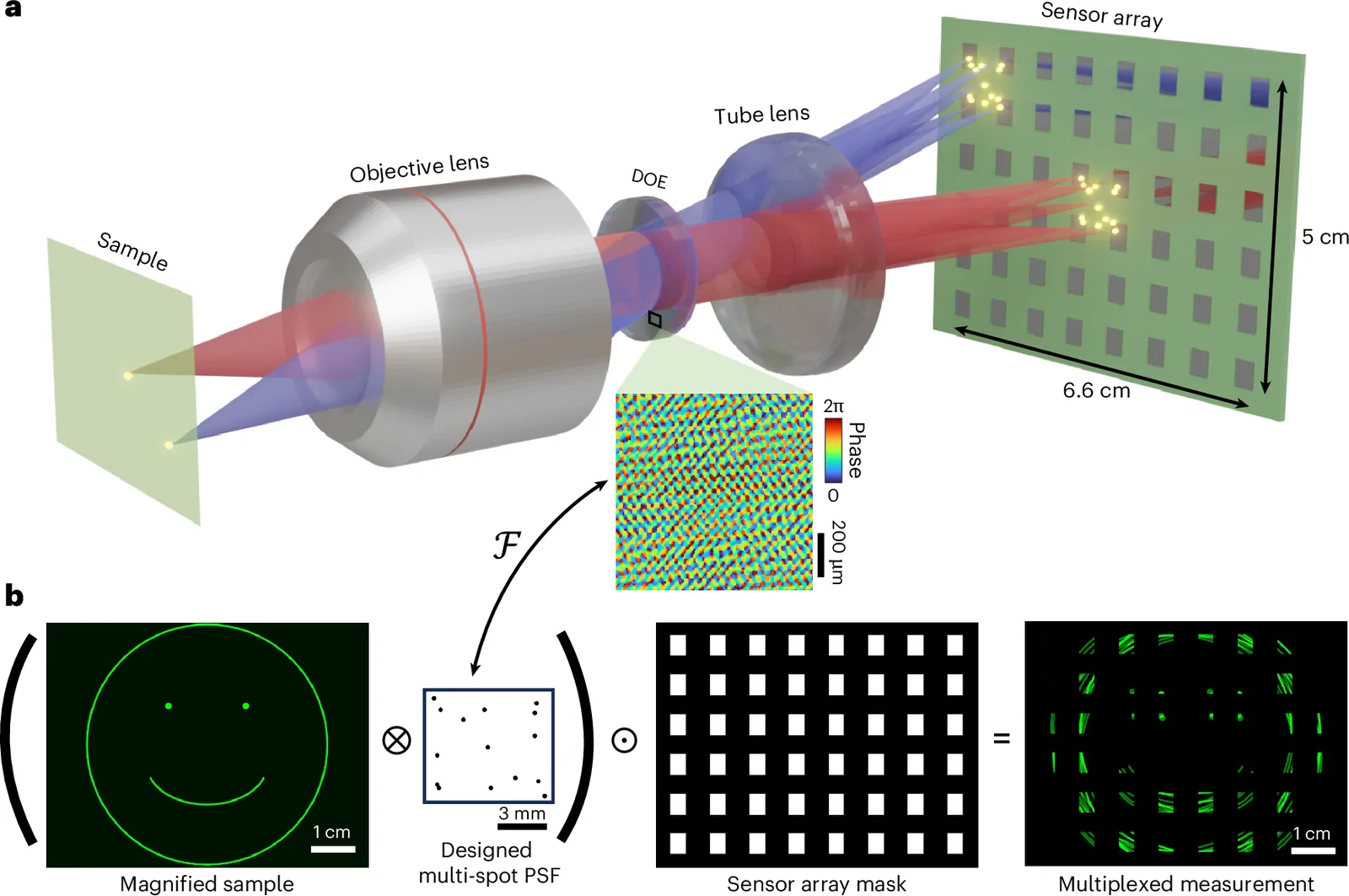
Overview of our large-scale compressive microscope. **a**, The imaging system uses an engineered DOE in the pupil plane to generate a distributed multi-spot PSF at the image plane. Images are captured by an array of 48 sensors arranged in a grid, with gaps between individual sensors. **b**, The system forward model is a masked convolution with the PSF, which compressively encodes the parts of the image that would normally fall on the gap areas. A computational inverse problem then recovers the full-scale image.

Here we present a new computational microscope that starts with a multi-sensor array and further improves SBP to achieve single-shot gigapixel-scale imaging (Fig. 1). In contrast to previous multi-camera systems, ours treats the sensor array as a single large-format sensor, without associating each individual sensor with its own lens. This keeps the optical design simple but results in areas between the sensors where light is not collected. To fill in these inter-sensor gaps and solve for the full-scale image, using only the data captured at the sensors (comprising only ~22% of the total area), we use point spread function (PSF) engineering and a compressed sensing reconstruction approach. Our PSF design is optimized to maximize the rank of the forward model, which we evaluated using a triple correlation between the PSF, itself, and the sensor array support (Supplementary equation (2)). The resulting PSF consists of 15 focused spots, which generates 15 copies of the sample image, arranged such that every point in the sample contributes light to at least one of the sensors. The multi-spot nature of our PSF lends itself to a direct convolution approach, enabling computational efficiency for the inverse problem. To implement this PSF, we fabricated a 64-level diffractive optical element (DOE), described in the ‘DOE fabrication’ section, using greyscale lithography and placed it in the Fourier plane of a nearly 4*f* system. Including both inter-sensor interpolation and extra-sensor extrapolation, we achieve an effective FOV >5.4× larger than the active sensor area. Conveniently, our method does not require a physical PSF calibration step and is robust to system perturbations. We show experimental results for structural and functional (calcium dynamics) microscopy of dozens of freely moving *Caenorhabditis elegans* across up to multiple square centimetre FOVs at micrometre resolutions at 120 fps.

## High-throughput microscopy with a diffractive pupil

Our microscope is a pupil-coded imaging system, consisting of an objective and tube lens with a DOE placed in the pupil plane and a two-dimensional (2D) array of sensors acting as a ‘super sensor’ in the image plane (Fig. 1). The DOE creates a sparse but extended PSF of 15 delta-like spots (see the ‘DOE design’ section for design considerations). In combination with compressed sensing reconstruction algorithms, this enables not only filling of the gaps between the sensors in the array but also extrapolation beyond the outermost sensors (Supplementary Section 2). Our ‘super sensor’ consists of a 6 × 8 array of 48 8-bit monochrome sensors (3,136 × 4,096 pixels, 1.1 μm pixel pitch, 9 mm centre-to-centre spacing) for a total of 0.617 gigapixels (GPs) captured in each snapshot (without downsampling), whose rectangular hull covers an area of 4.95 cm × 6.64 cm. The sensor array frame rate has two bottlenecks: the data transfer bandwidth of 6.17 GB s^−1^ and the sensor readout rate. At full 0.617 GP sampling, the frame rate is bandwidth-limited to 10 fps, which in practice is too slow for imaging freely moving *C. elegans* at high resolution. To achieve higher frame rates, we use 4 × 4 pixel binning (38.5 MP per snapshot) to achieve 120 fps (readout-limited). Since our method requires demixing of overlapped images as part of an underdetermined inverse problem (we solve for more pixels than we measure), samples should be sparse (see Supplementary Fig. 5 for a quantification of the sparsity requirements).

We report two different imaging configurations: fluorescence and dark field. Since dark-field imaging has very little background, it promotes sample sparsity. Both configurations share the same tube lens (Jupiter-36B, *f* = 250 mm, *f*/3.5) and DOE, but use different objective lenses. In dark-field imaging mode, we use an FJW Industries lens (*f* = 90 mm, *f*/1.0) as the objective (for a total magnification of ~2.78×), 4 pseudo-collimated (Thorlabs ACL2520U-A, *f* = 20 mm) off-axis green LEDs (Thorlabs M530L4) as the illumination source and a 514/2 nm band-pass filter (Alluxa) in the pupil plane. The narrowband filter reduces the radial spectral blurring of the non-zero diffraction orders, which would otherwise degrade spatial resolution (Supplementary Section 4).

In fluorescence imaging mode, we replace the objective with a Rodenstock lens (*f* = 42 mm, nominal numerical aperture (NA) = 0.7, design wavelength = 532 nm) for a total magnification ~6×. We use a high-power blue LED as the wide-field Köhler illumination source (Thorlabs SOLIS-470C), a 480/40 nm excitation filter (Chroma) and a 530/55 nm fluorescence emission filter (Edmund) placed directly in front of the DOE. Compared with dark-field mode, the emission filter bandwidth is significantly broader to compensate for weaker signals in fluorescence mode. Here we take advantage of the fact that the objective lens was designed for one particular wavelength, resulting in strong axial chromatic aberrations. Thus, each wavelength focuses at a different depth plane, so the PSF induced by the DOE still has sharp peaks, albeit with heavy tails that contribute to out-of-focus background. However, we empirically found that the increased background was offset by the increase in signal-to-noise ratio (SNR) from having a substantially broader emission filter bandwidth (Supplementary Section 4).

### DOE design

The DOE is a phase mask placed in the pupil plane to generate a multi-spot PSF, which enables filling in the gaps between sensors. To this end, there were five chief criteria we optimized for when designing the PSF: (1) at every point in the sample FOV, the PSF will result in light falling on at least one sensor, (2) the PSF should have minimal translational ambiguity, (3) the PSF is as narrow in extent as possible to minimize spectral blurring (owing to the wavelength dependence of diffraction), (4) the PSF is as sparse as possible to minimize the impact of read noise, and (5) the PSF is robust to scaling errors, which may arise in practice owing to deviations in the lens focal lengths. The second criterion ensures maximization of the rank of the linear forward model, which can be efficiently evaluated using a triple correlation (Supplementary equation (2)) between the PSF, itself, and an indicator function, Mask(*x*, *y*), that is 1 where there is a pixel and 0 in the gaps of the sensor array plane. This triple correlation is a generalization of the typical PSF autocorrelation procedure extended to the case of a sensor with erasures in the gap areas. See Supplementary Section 1 for details on PSF optimization.

On the basis of these design criteria, we chose a PSF that consists of an asymmetric distribution of 15 impulses (Supplementary Section 1.3, Supplementary Fig. 2 and Supplementary Table 1). This design enables a simple (windowed) convolution as our forward model, concentrates light into a sparse set of spots for mitigating loss in SNR, and achieves a spatial extent (6.8 mm × 6 mm) wider than the largest gap in the sensor array (5.55 mm × 4.49 mm) to ensure that all points in the FOV contribute to the measured light at the sensors. Another benefit of the extended PSF is that we can extrapolate beyond the rectangular hull of the sensor array (4.95 cm × 6.64 cm) by the width of the PSF (that is, to 5.63 cm × 7.24 cm; Supplementary Section 2).

We solved for the pupil phase pattern, *ϕ*(*x*, *y*), that generates this PSF using the Gerchberg–Saxton algorithm^21^, modified to discretize the phase values to 64 levels at each iteration. We then converted *ϕ*(*x*, *y*) to height values, *h*(*x*, *y*):

1 $$h(x,y)=\frac{\lambda \phi (x,y)}{2\pi (n-1)},$$

based on the refractive index of the photoresist (S1813) at a design wavelength of *λ* = 515 nm (*n* = 1.6546). We fabricated the custom 64-level DOE based on *h*(*x*, *y*) using greyscale lithography, with a maximum height variation of 0.787 μm (corresponding to 2π at *λ* = 515 nm), a pixel size of 6 μm and a diameter of 39.8 mm (‘DOE fabrication’ section).

### Computational forward modelling and reconstruction

In principle, the forward model describing our imaging system is a masked 2D convolution:

2 $$P({\bf{r}})=\left(\rm{psf}({\bf{r}})\otimes \rm{Obj}({\bf{r}})\right)\odot \rm{Mask}({\bf{r}}),$$

where **r** = (*x*, *y*) is the 2D position, *P*(**r**) is the forward prediction of the data, ⊙ denotes element-wise multiplication, psf(**r**) is the designed multi-impulse PSF, ⊗ denotes 2D convolution, Obj(**r**) is the 2D object and Mask(**r**) is an indicator function defining whether there is a camera pixel at a given **r** position. Since the PSF is a collection of near-delta functions, the convolution in equation (2) can be interpreted as an incoherent superposition of 15 shifted copies of the object via the sifting property:

3 $$\begin{array}{ccc}P({\bf{r}}) & = & \left(\sum _{i}{A}_{i}\delta ({\bf{r}}-{{\Delta }{\bf{r}}}_{i})\otimes \mathrm{Obj}({\bf{r}})\right)\odot \mathrm{Mask}({\bf{r}})\\ & = & \left(\sum _{i}{A}_{i}\mathrm{Obj}({\bf{r}}-{{\Delta }{\bf{r}}}_{i})\right)\odot \mathrm{Mask}({\bf{r}}),\end{array}$$

where *A*_*i*_ is the amplitude and Δ**r**_*i*_ = (Δ*x*_*i*_, Δ*y*_*i*_) is the relative position of the *i*th impulse of the PSF.

In practice, the forward model is not shift invariant owing to distortions and aberrations in the objective and tube lenses. A common way to model distortion is with a radial model, whereby the magnification, *M*(**r**), varies radially (for example, barrel and pincushion distortion):

4 $$M({\bf{r}})=1+\mathop{\sum }\limits_{i=1}^{m}{a}_{i}{\left\vert {\bf{r}}-{{\bf{r}}}_{0}\right\vert }_{2}^{2i},$$

which for simplicity is normalized by the nominal magnification, *M*_0_, to 1 (that is, so that the actual magnification is *M*_0_*M*(**r**)), ${\{{a}_{i}\}}_{i = 1}^{m}$ are the *m* distortion coefficients, **r**_0_ = (*x*_0_, *y*_0_) is the centre of distortion, and ∣ ⋅ ∣_2_ is the L2 norm. This model assumes a rotationally symmetric imaging system; however, our microscope is not rotationally symmetric since the DOE has an asymmetric diffraction pattern. Thus, we modelled distortions via two separable radial distortion models, one for the objective and one for the tube lens:

5 $$\begin{array}{ll}{M}_{obj}({\bf{r}})&=1+\mathop{\sum }\limits_{i=1}^{m}{a}_{i}^{obj}{\left\vert {\bf{r}}-{{\bf{r}}}_{0}^{obj}\right\vert }_{2}^{2i},\\ {M}_{tube}({\bf{r}})&=1+\mathop{\sum }\limits_{i=1}^{m}{a}_{i}^{tube}{\left\vert {\bf{r}}-{{\bf{r}}}_{0}^{tube}\right\vert }_{2}^{2i}.\end{array}$$

With this model, each diffracted copy undergoes a unique distortion operation (Supplementary Fig. 8). For example, given a point in the object plane at position **r**_*p*_ and a 2D spatial shift Δ**r**_*i*_ corresponding to the *i*th impulse of the PSF, the point experiences a magnification of

6 $$M({{\bf{r}}}_{p},\Delta {{\bf{r}}}_{i})={M}_{0}{M}_{obj}({{\bf{r}}}_{p}){M}_{tube}(-{{\bf{r}}}_{p}-\Delta {{\bf{r}}}_{i}).$$

We use this model (equations (5) and (6)) to generate the PSF at any desired position in the FOV for a fully shift-variant model. By using a direct convolution approach, as opposed to FFT-based, we benefit from a few more computational efficiencies. First, the multi-impulse PSF only has 15 non-zero points contributing to the convolution. Second, we only needed to compute the convolution at spatial positions where there exists a sensor pixel (rather than for the full FOV, followed by masking). Thus, this shift-variant forward model can be written within the loss function as

7 $$\begin{array}{c}\mathrm{Loss}=\frac{1}{| S| }\sum _{{\bf{r}}\in S}{\left(\left(\sum _{i}{A}_{i}\mathrm{Obj}\left(M({\bf{r}},{\Delta }{{\bf{r}}}_{i})({\bf{r}}-{{\Delta }{\bf{r}}}_{i})\right)\right)-I({\bf{r}})\right)}^{2}\\\,\,\,\,\,+{\lambda }_{1}\mathrm{TV}(\mathrm{Obj})+{\lambda }_{2}| \mathrm{Obj}{| }_{1},S=\{{\bf{r}}| \mathrm{Mask}({\bf{r}})=1\},\end{array}$$

where the first term is the mean square error (MSE), with the sum defined over positions in the image plane, *I*(**r**), that contain detection pixels. We found that this fully shift-variant model was better than a patch-based shift-invariant model in terms of memory and accuracy, even when using FFT-based convolutions (Supplementary Section 3). The second term is an isotropic total variation (TV) regularizer and the third term is an L1 regularizer. We minimized equation (7) using gradient descent for each time point, also applying a non-negativity constraint at every iteration. Each frame took <5 s to reconstruct (that is, 50 iterations at >10 iter s^−1^) on a 24-GB GPU (Nvidia RTX 3090). While our direct convolution approach enabled fast reconstructions and spatially variant distortion correction, a patch-based FFT approach could potentially yield more accurate results by modelling the aberrations of the individual PSF impulses.

## Results

We first characterized the spatially varying resolution, field curvature and depth of field (DOF) of both imaging modes by translating and acquiring *z*-stacks of a negative USAF target across the entire FOV. From these image stacks, we computed an image sharpness metric based on the mean square image gradient to identify the most in-focus image across the extended FOV (Supplementary Figs. 11–15). Dark-field imaging mode can resolve down to group 8 element 4 (2.76 μm full-pitch resolution) across most of the extended FOV, and at some positions, group 9. Thus, when we operate the sensors at 4× downsampling, the optical resolution and Nyquist sampling resolution (1.58 μm pixel size at the sample) are nearly matched across the reconstructed ~20.0 × 26.4 mm^2^ FOV, for a total per-frame pixel count of 12,621 × 16,655 = 210 MPs and a total effective imaging throughput of up to ~25.2 GP s^−1^. While the fluorescence imaging mode can resolve down to group 9 near the centre, it has significant aberrations in the periphery. However, the fluorescence imaging mode can resolve down to group 7 element 5 (4.38 μm full-pitch resolution) across most of the 9.3 × 9.3 mm^2^ FOV. Thus, our fluorescence imaging resolution is aberration-limited with an effective imaging throughput of up to ~2.17 GP s^−1^.

Plotting the best focus position and subtracting the plane of best fit (to account for tilt in the translational scan) reveal minimal field curvature across the FOV in both imaging modes (Supplementary Fig. 11a,e). In addition, we computed the DOF across the extended FOV, defined as the axial full-width at half-maximum of the sharpness metric (Supplementary Fig. 11b,f). As expected, the DOF is larger further away from the centre of the FOV, owing to aberrations. The fluorescence imaging mode has a larger DOF across the entire FOV than dark field, owing to axial chromatic aberrations. Sample sharpness curves across all three spatial dimensions are shown in Supplementary Fig. 11c,d,g,h.

### Imaging of static samples

**Fig. 2:**
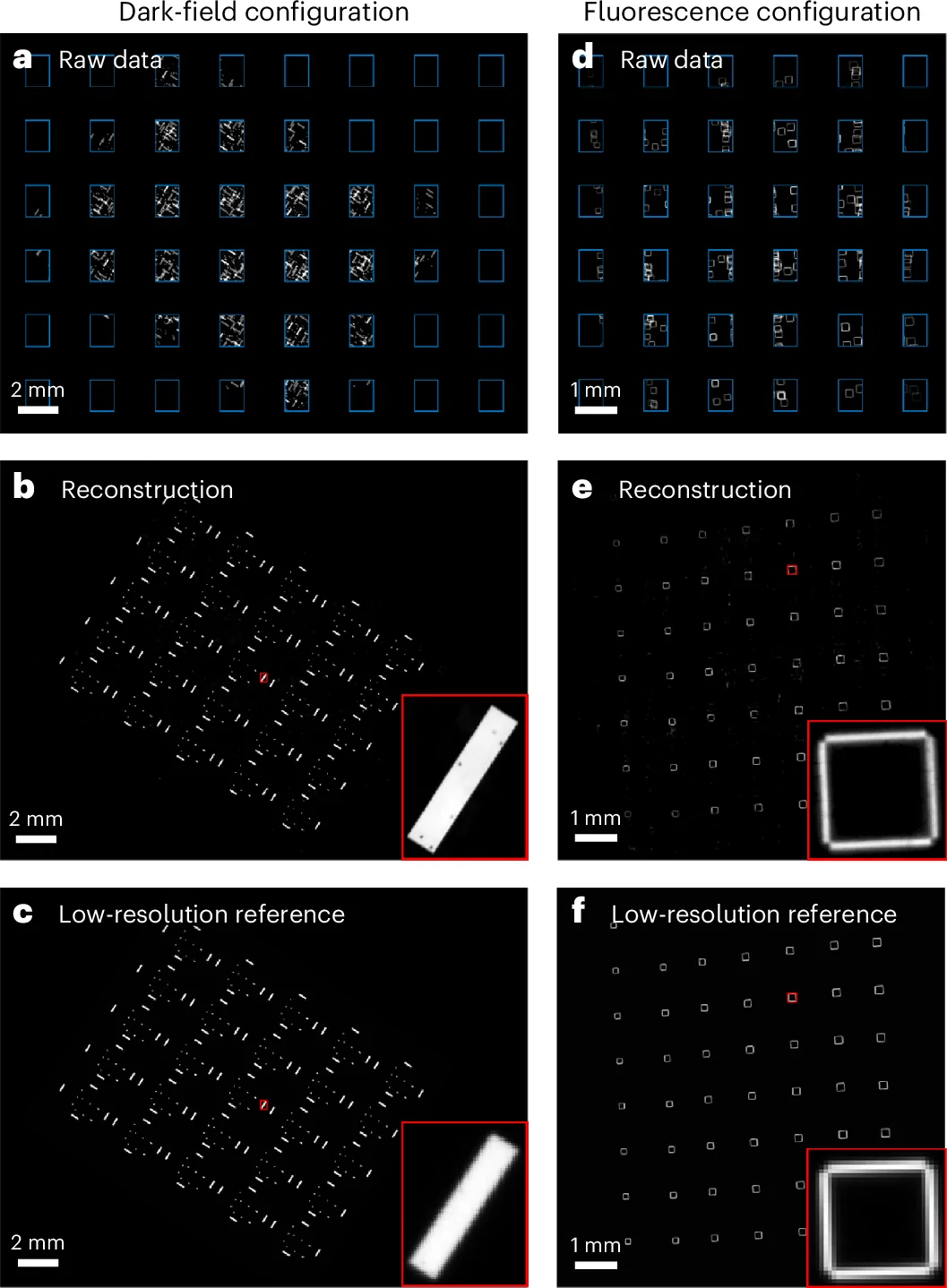
Wide-field, high-resolution reconstructions of static samples. Single-frame images reconstructed from captured data of photolithography masks using dark-field configuration (**a**,**b**,**c**) and fluorescence configuration (**d**,**e**,**f**). For the latter, we use a green LED, whose spectrum roughly matches that of green fluorescent protein. **a**,**d**, Raw captured data. **b**,**e**, Reconstructions of the full-scale image, with a zoom-in on the area denoted by the red box. **c**,**f**, Low-resolution reference image, taken with a 1×/NA = 0.04 objective, with a zoom-in on the same area.

Next, we imaged static photolithography masks and compared our reconstructions with those captured using a traditional Nikon microscope with a low-magnification objective (1×/NA = 0.04), covering an FOV of 13.3 × 13.3 mm^2^ with a pixel size of 6.5 μm. Our raw, multiplexed data and the corresponding reconstructions are shown in Fig. 2 for both imaging configurations (dark field and fluorescence). In the raw data, we clearly see the superimposed multiple copies of the sample image. The global features of our reconstructions closely match those of the low-resolution traditional images. However, the zoom-ins show that our reconstructions have higher resolution (Fig. 2b,c,e,f).

### Imaging of freely moving *C. elegans*

**Fig. 3:**
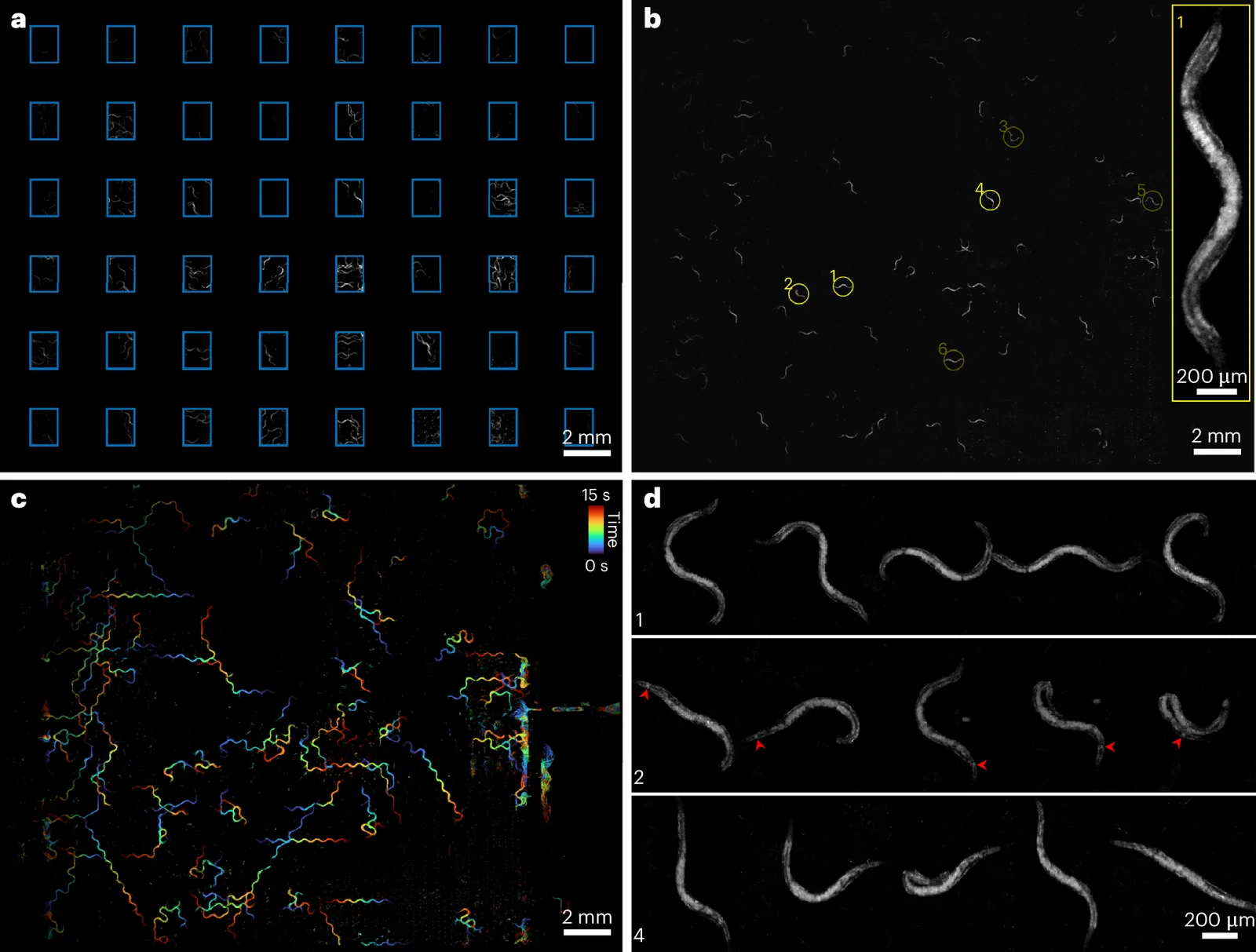
High-speed (120 fps) dark-field imaging of wild-type *C. elegans* at high resolution across a 20.0 × 26.4 mm^2^ FOV. **a**, Raw data at a single time frame, shown with each sensor in its correct relative position (with the static background subtracted). The blue outlines indicate the sensor boundaries. **b**, Reconstruction of the full-scale image. The circled and numbered worms were tracked in the full video. Inset: a zoom-in of worm number 1. **c**, The full video reconstruction, colour-coded by time, showing worms moving around the array of pillars. **d**, Select frames of the video (separated by 3.7 s) offset and superimposed to show the motion of a few of the worms. The red arrowheads mark the posterior end of the gut, which can be tracked throughout the video. See Supplementary Videos 1 and 2 for the full raw video and reconstructions, respectively.

To demonstrate the utility of our method in imaging highly dynamic samples, we imaged dozens of freely moving *C. elegans* at high speed (120 fps) for 15 s using both imaging modes. In dark-field mode, we performed structural imaging of wild-type *C. elegans* freely moving across a 2 cm × 2 cm microfluidic device containing a hexagonal grid of cylindrical pillars (Fig. 3) to mimic their natural soil environment^22,23^. Before video reconstruction, we estimated and subtracted out the static background, removing dark-field signals from the pillars and any other static structures, leaving only the signal from the *C. elegans*. The full background-subtracted multi-sensor video data is shown in Supplementary Video 1, a single frame of which is visualized in Fig. 3a. The multiplexed copies of the worms are computationally demixed, leading to a contiguous, gap-free video reconstruction of the worms and their trajectories across the 15 s video (Supplementary Video 2). A single frame of and a time-encoded image of the whole video are shown in Fig. 3b,c, respectively. The high-resolution, contiguous nature of our reconstructions enabled tracking of the worms and some structures within them (for example, the posterior end of the gut, circled in red in Supplementary Video 2 and indicated by red arrowheads in Fig. 3d).

**Fig. 4:**
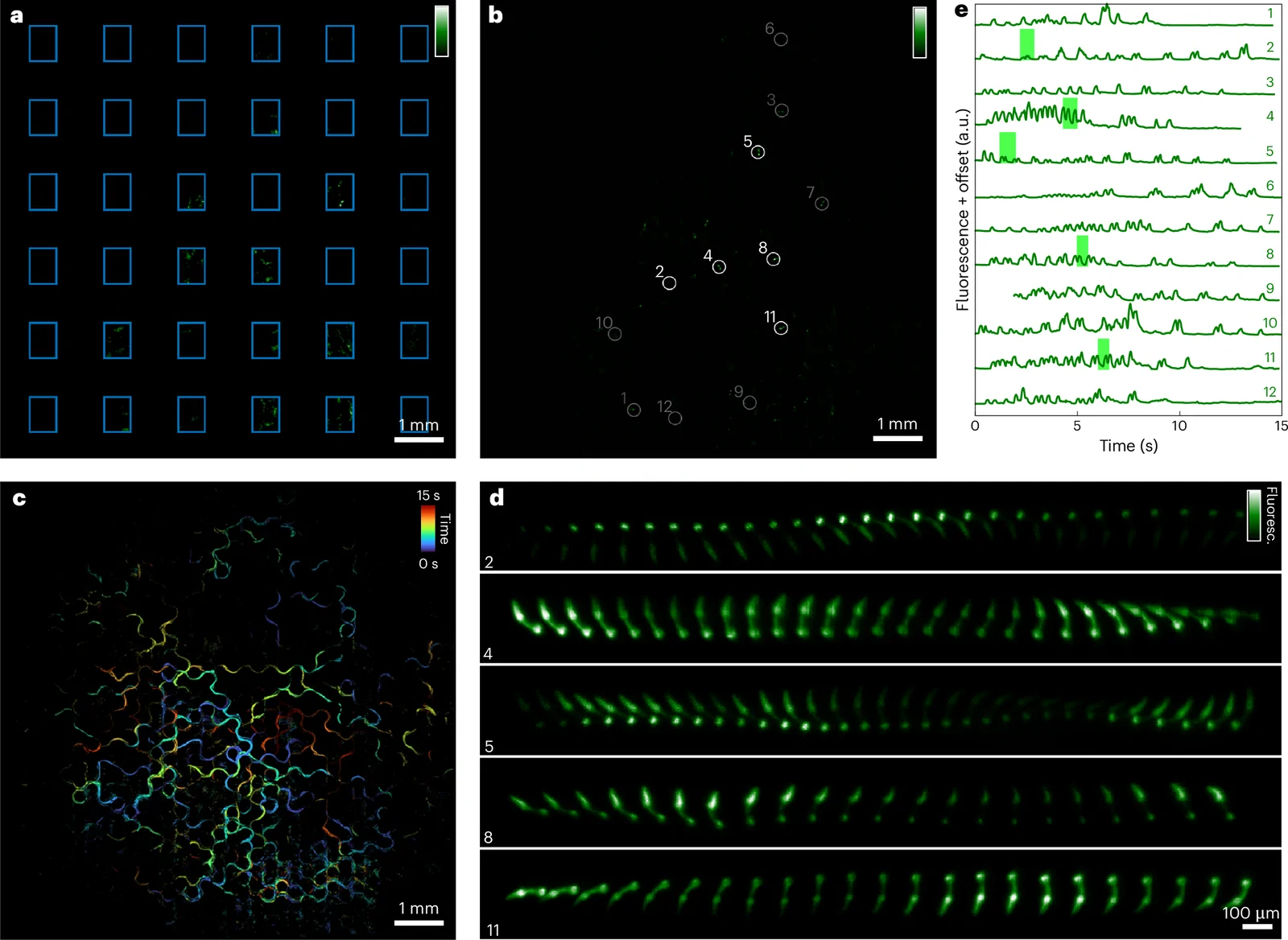
High-speed (120 fps) functional fluorescence imaging of *C. elegans* expressing GCaMP8f in their pharynx. **a**, Raw data (with static background subtraction) of a single frame. The blue outlines indicate the sensor boundaries. See Supplementary Video 3 for full video. **b**, Reconstruction of the corresponding full-scale image. The circled and numbered worms are analysed in **d** and **e** as well as in Supplementary Video 4. **c**, The full 15 s reconstructed video, colour-coded by time, as worms move around the array of pillars. **d**, Multiple frames (separated by 25 ms) offset and superimposed to show the motion of 5 selected worms. Fluoresc., fluorescence. **e**, Fluorescence traces for the worms indicated in **b**. The green highlighted time windows correspond to the image sequences plotted in **d**. See Supplementary Videos 3 and 4 for the full raw video and full video reconstructions and tracking of all 12 highlighted worms, respectively.

Finally, using the fluorescence imaging mode, we imaged freely moving *C. elegans* expressing GCaMP8f in the pharynx^24^. Here the GCaMP8f indicator responds to the influx of intracellular calcium ions during rapid pharyngeal pumping events of *C. elegans* (up to 4–5 Hz) as part of their feeding behaviour. The worms were loaded in a smaller, 8 mm × 8 mm microfluidic chip with the same hexagonal array of pillars^25^. Supplementary Video 3 shows the raw, background-subtracted, multi-sensor video data, a single frame of which is shown in Fig. 4a. The corresponding reconstructions are visualized in Fig. 4b,c and Supplementary Video 4. Both the raw data and reconstructions appear sparser than their dark-field counterparts (Fig. 3) because only the pharynx is fluorescently labelled. From the video reconstruction, we were able to track 12 individual worms and extract their pumping behaviour from the fluorescence traces (Fig. 4e). A few sample pumping events are illustrated in Fig. 4d.

## Discussion

We present a computational microscope that uses a threefold strategy to expand spatiotemporal throughput: a discontiguous array of 48 independent camera sensors, diffractive pupil coding to further expand the FOV by >5.4× and a computational reconstruction algorithm. Specifically, we designed and fabricated a DOE that, when placed in the pupil plane of our microscope, creates a multi-impulse PSF that encodes and compresses information from the gaps between the 48 sensors, enabling minimally ambiguous computational reconstruction. Our method, capable of achieving micrometre resolution over multiple square centimetre FOVs at 120 Hz frame rates, stands out among the highest-throughput microscopes at 25.2 GP s^−1^. With these capabilities, we demonstrate high-resolution structural and functional imaging of dozens of *C. elegans* in parallel.

There are several avenues for future investigation that could further improve our microscope. We could extend our system to 3D by modifying the DOE design to include depth encoding^26,27^. An important limitation of our design is that it requires lenses with relatively high SBPs, capable of achieving high resolution over a large FOV, which is practically difficult to achieve. As a result, our microscope contains aberrations and vignetting (see corners of Figs. 3c and 4c) that prevent diffraction-limited resolution across the entire FOV (Supplementary Figs. 12–15). Fabricating an even larger DOE would further improve the theoretical diffraction-limited performance, although in practice it would increase aberrations. To partially bridge the resolution gap, we could calibrate the spatially varying PSF and incorporate it into the forward model (‘High-throughput microscopy with a diffractive pupil’ section). This could be particularly useful for the fluorescence imaging mode, whose PSF contains both geometric and chromatic aberrations owing to the DOE and objective (Supplementary Section 4). A hybrid diffractive–refractive design could also be used to reduce these DOE-induced chromatic aberrations^28^. Careful PSF calibration with lensless approaches^29,30^ could also help alleviate some of these challenges with aberration, albeit at the cost of SNR.

Our PSF design could also potentially be improved. Here we used a design heuristic based on a discrete distribution of impulses that maximizes the rank of the linear forward model while balancing SNR considerations (Supplementary Section 1 and Supplementary Fig. 17). Our heuristic also reduces the column correlations of our linear model by breaking PSF symmetry so that the maximum PSF overlap between any two field positions is a single impulse. However, there are infinitely many PSFs that satisfy these criteria. Furthermore, such a design presupposes sparsity in the native space domain, which we enforced by ensuring sparse distributions of *C. elegans*. Thus, there is room for future investigation to further optimize the PSF design for more general samples based on sparsity in other domains or in a data-driven fashion^31^. Such multiplexing PSF designs must be balanced with practical constraints, such as low photon counts typical of high-speed fluorescence imaging, non-negativity of the PSF and chromatic aberrations of larger PSFs.

Despite these challenges, we have demonstrated experimentally the high-speed, high-resolution, wide-FOV imaging capabilities conferred by our microscope’s highly parallelized and multiplexed design. With these new capabilities, our microscope can open up new applications not only in functional and behavioural imaging of large populations of freely moving model organisms but also in diagnostic imaging for cell counting, detection of rare species and semiconductor inspection.

## Methods

### *C. elegans* preparation

*C. elegans* animals were maintained under standard conditions and fed OP50 bacteria^32^. Wild-type worms were strain N2 (Bristol). The other strain used in functional imaging was INF418 *nonEx106[myo-2p::GCaMP8f::unc-54 3’UTR]*^24^.

Structural and functional imaging were performed using monolayer microfluidic devices^22,25^, featuring arena areas 2 cm × 2 cm × 70 μm or 8 mm × 8 mm × 70 μm in size, respectively, where 200-μm-diameter cylindrical pillars were arrayed hexagonally with a 300 μm centre-to-centre spacing. In this structured environment, worms were inducted to crawl rather than swim^23,33^. Before each experiment, 150 young adult hermaphrodites were collected from cultivation plates using 2 ml of S-basal buffer (100 mM NaCl and 50 mM potassium phosphate; pH 6.0), washed twice in fresh 2 ml of S-basal buffer to remove excess bacteria, drawn into a loading tubing using a 1 ml syringe and gently introduced into the microfluidic devices. These steps were completed within 10 min. Then, gravity-fed S-basal was allowed to flow through the arena for an additional 10 min, and the loaded worms were left to crawl freely for 1 h before video recording began, allowing them to enter a starved state.

### DOE fabrication

The DOE was fabricated on a 50.8-mm-diameter, 500-μm-thick glass substrate using greyscale lithography (Supplementary Fig. 16). A layer of positive-tone photoresist (MICROPOSIT S1813 G2) was spin-coated at 1,000 rpm for 60 s and baked on a hotplate at 110 °C for 2 min. The design was patterned onto the sample with a laser pattern generator (DWL66+, Heidelberg Instruments). Post-exposure, the sample was heated at 50 °C for 1 min and developed in AZ Developer (1:1) for 1 min and 20 s. A calibration sample, prepared and developed in the same way, was used to map photoresist depths to laser intensities from the pattern generator before the final write. The DOE pattern consisted of a 6,638 × 6,638 matrix of heights, with each index measuring 6 μm × 6 μm. The maximum depth of the DOE pattern was 0.787 μm. A confocal microscope (Olympus LEXT OLS5000) was used to measure the heights of several outermost pixels in various locations on the sample (Supplementary Fig. 16c). The average height difference between fabrication and ideal design was approximately 20 nm, with a standard deviation of 30 nm, which would have a negligible impact on the accuracy of the PSF (Supplementary Fig. 16d).

### Data preprocessing

Before computational reconstruction (‘Computational forward modelling and reconstruction’ section), we first performed two preprocessing steps. First, we estimated the background owing to unrejected fluorescence excitation, dark-field signals from the pillars in the chips containing the *C. elegans*, and any other signals that remain static throughout the video recording. To do this, for every pixel in the raw recordings, we computed the *n*th percentile across time. We then subtracted this estimated background from each frame of the video. In the second step, we estimated the imaging system distortion and DOE orientation (‘Computational forward modelling and reconstruction’ section) by jointly optimizing them with the reconstruction of a single frame from the video (or a superposition of multiple frames).

### Tracking

Tracking of *C. elegans* was performed almost entirely automatically using Trackpy in Fig. 3 and Supplementary Video 2. Automatic tracking of the GCaMP8f-expressing *C. elegans*, however, was much more challenging, given the fluorescence signal fluctuations during pharyngeal pumping. Thus, we first performed automatic tracking using Trackpy, followed by manual tracking of the 12 worms highlighted in Fig. 4 and Supplementary Video 4.

## Online content

Any methods, additional references, Nature Portfolio reporting summaries, source data, extended data, supplementary information, acknowledgements, peer review information; details of author contributions and competing interests; and statements of data and code availability are available at https://doi.org/10.1038/s41566-026-01974-4.

## Supplementary information

Supplementary Information Supplementary Figs. 1–17 and Sections 1–8.

Peer Review File

Supplementary Video 1 Raw multiplexed multi-sensor video of wild-type *C. elegans* under dark-field illumination.

Supplementary Video 2 Reconstructed wide-field, high-resolution, high-speed video of wild-type *C. elegans*.

Supplementary Video 3 Raw multiplexed multi-sensor fluorescence video of *C. elegans* expressing GCaMP8f in the pharynx.

Supplementary Video 4 Reconstructed wide-field, high-resolution, high-speed video of *C. elegans* expressing GCaMP8f in the pharynx.

## Data Availability

The raw data underlying the results are available at https://doi.org/10.5061/dryad.tmpg4f5b6.

## Code availability

The image reconstruction code is available via GitHub at https://github.com/kevinczhou/diffractive-compressive-array-microscopy.
