# Supplementary material for: Large-scale compressive microscopy via diffractive multiplexing across a sensor array
Source: Nat Photonics. 2026 Jul 28;20(10):1240–6. doi: 10.1038/s41566-026-01974-4 (PMC13626960; doi:10.1038/s41566-026-01974-4)
Supplement: Supplementary file 1 [file 41566_2026_1974_MOESM1_ESM.pdf]

nature photonics

Supplementary information https://doi.org/10.1038/s41566-026-01974-4

Large-scale compressive microscopy via diffractive multiplexing across a sensor array

In the format provided by the authors and unedited

Supplementary information

S1. PSF design details

In this section, we explain in more detail the five criteria mentioned in the main text that we optimized for, followed by the exact optimization procedure.

S1.1. Criteria for a good PSF

Pan-visibility. First and foremost, the PSF should always be visible across the entire FOV on at least one sensor, including when the PSF is centered within a gap between sensors. This criterion ensures that we obtain a continuous, gap-free reconstruction. To fulfill this criterion, the PSF needs to be at least as large as the largest gap in the sensor array, which is 5.5504 mm by 4.4944 mm in the image plane. For example, a 3×3 grid of dots with these dimensions satisfies this criterion (Fig. S1a); however, it has ambiguities (Fig. S1b) that renders reconstruction challenging, which leads us to the second criterion.

Minimal translational ambiguity. Specifically, no two PSF positions in the FOV should give rise to the same measurement and they should be as dissimilar as possible (low autocorrelation). This argument is analogous to that of the standard, linear shift-invariant (LSI) imaging system (with no gaps), as minimizing PSF autocorrelation results in maximizing the frequency content of the MTF. Here, due to the discontinuous FOV, this criterion is harder to satisfy. For example, the aforementioned 3×3 PSF fails this test (Fig. S1b), but would not fail if there were no gaps (although this PSF is autocorrelated).

These first two criteria have intuitive interpretations, but could be understood more rigorously by examining the imaging problem more abstractly as a simple linear equation. To be concrete, let

$$\underset{(m\times 1)}{\mathbf{b}} = \underset{(m\times n)}{\mathbf{A}}\,\underset{(n\times 1)}{\mathbf{x}} \tag{S1}$$

describe a simplified forward model of our imaging system, ignoring distortion and aberrations, where $\mathbf{b}$ is a vector of length m, corresponding to the number of pixels across the sensor array, $\mathbf{A}$ is a Toeplitz matrix with the rows corresponding to shifted copies of the PSF, and $\mathbf{x}$ is a vector of length n, corresponding to the number pixels in the object reconstruction. Since there are gaps in the sensor array, $n > m$, so that this is an underdetermined problem. Further, since we're modeling an incoherent imaging system, the PSF and therefore the entries of $\mathbf{A}$ are strictly nonnegative. The best we can do for a general imaging system is maximize the rank of $\mathbf{A}$ (i.e., m); however, not all PSFs that maximize the rank of $\mathbf{A}$ are desirable. For example, a delta function PSF is the "best" PSF in terms of having zero translational ambiguity – in other words, its corresponding $\mathbf{A}$ not only has maximal rank m, but also has orthogonal rows. However, such an $\mathbf{A}$ matrix is effectively an $m \times m$ identity matrix, as the remaining columns are all zeros – in other words, certain parts of $\mathbf{x}$ would never be sampled, resulting in a gappy reconstruction.

Thus, if we want $\mathbf{A}$ to be a nonnegative, $m \times n$ matrix without any zero-only columns, we must accept a PSF that has non-zero autocorrelation (i.e., the rows of $\mathbf{A}$ cannot be mutually orthogonal). Nevertheless, we want the rows and columns of $\mathbf{A}$ to be as close to orthogonal as possible – doing so minimizes the condition number of $\mathbf{A}$. In compressive sensing, this is minimizing the mutual coherence of $\mathbf{A}$ (the max normalized correlation between any two columns of $\mathbf{A}$). Doing so ensures that no two positions in the FOV give rise to the same measurement on the sensor array. This minimization is also analogous to minimizing the autocorrelation of the PSF in a standard, gap-free LSI imaging system to improve its MTF, which we can generalize to our system by modulating the autocorrelation with the sensor array mask, $Mask(\mathbf{r})$. In particular, when testing candidate PSFs, rather than working with the very large $\mathbf{A}$ matrix, we can evaluate a normalized

triple correlation:

$$C(\mathbf{\Delta r}, \mathbf{\Delta r'}) = \frac{\sum_r psf(\mathbf{r}) psf(\mathbf{r} + \mathbf{\Delta r}) Mask(\mathbf{r} + \mathbf{\Delta r'})}{\sum_r psf(\mathbf{r})^2 Mask(\mathbf{r} + \mathbf{\Delta r'})}, \tag{S2}$$

where we would like $C(\mathbf{\Delta r}, \mathbf{\Delta r'})$ to be close to 0 when $\mathbf{\Delta r} \neq (0,0)$. The reason why *Mask* also needs to be cross-correlated is that the PSF should have low autocorrelation, regardless of whether it is centered in a gap or on a sensor. In practice, it is sufficient to evaluate Eq. S2 for *Mask* corresponding to 3×3 sensors, as our imaging system is "periodically LSI".

For a gap-free imaging system with a PSF containing k impulses, the lowest possible max autocorrelation for $\mathbf{\Delta r} \neq (0,0)$ would be $1/k$ – at most one impulse overlapping, which is only achievable if the PSF doesn't have inversion symmetry (proof: PSF points $\mathbf{\Delta r}_i$ and $\mathbf{\Delta r}_j$ will simultaneously overlap with $-\mathbf{\Delta r}_j$ and $-\mathbf{\Delta r}_i$, respectively). Thus, our PSF design should not have inversion symmetry. However, for a gappy sensor, the triple correlation could be higher than $1/k$, because not all k impulses may be visible at a given position in the sensor plane (this inflation is accounted for by the presence of *Mask* in the denominator of Eq. S2).

Narrowest possible spatial extent. While the PSF needs to be larger than the largest inter-sensor gap, it should be no larger due to the lateral chromatic aberrations of the DOE. In particular, since the diffraction pattern off of the DOE scales with the wavelength, an off-axis spot experiences radial smearing (δr) proportionally with distance from the zero diffraction order,

$$\delta r \propto \frac{\Delta R \Delta \lambda}{\lambda}, \tag{S3}$$

where ΔR is the radial distance from the zero order, $\Delta\lambda$ is the bandwidth of the detected light and λ is the center wavelength (see Sec. S4 for further discussions). Practically, this equation places a limit on the bandwidth of the detected light for a given spot size on the sensor. For the darkfield imaging mode, we use a 2-nm bandwidth bandpass filter as a compromise between resolution and light throughput. However, for the fluorescence imaging mode, we take advantage of the wavelength-dependent focusing properties of the objective (i.e., axial chromatic aberration) to mitigate the radial blurring effects. In practice, we specify a bounding box within which to propose the random points that make up the PSF.

PSF sparsity. The fewer pixels into which our imaging system maps each point in the sample, the less sensor read noise we incur. This criterion also suggests that a good PSF design would be a collection of points. In practice, we have to balance this criterion with the minimal translational ambiguity criterion, which we found required a minimum number of points to satisfy. Furthermore, the more points, k, in the PSF, the smaller the max possible autocorrelation ($1/k$); however, we prioritized PSF sparsity due to the low light levels of our imaging applications.

Robustness to scaling errors. Due to experimental imperfections, the actual PSF size may not match the design PSF size. For example, since the PSF size is proportional to the tube lens focal length, any error therein would lead to an artifactually large or small PSF. Thus, we searched for a PSF that would satisfy all the other criteria over a broad range of magnification errors.

S1.2. PSF optimization procedure

The goal was to optimize a PSF parameterized by the positions of a distribution of points. Gradient-based methods are unsuitable because the gradients of the visibility and minimal ambiguity criteria with respect to the PSF point positions are zero almost everywhere. We thus opted for a gradient-free random search strategy, which involved systematically proposing random distribution points as the PSF candidates and checking adherence to the aforementioned criteria. In principle, there are an infinite number of suitable PSFs, so in practice, we ranked the suitable PSFs we obtained, based on their robustness to scaling errors. We caution that the

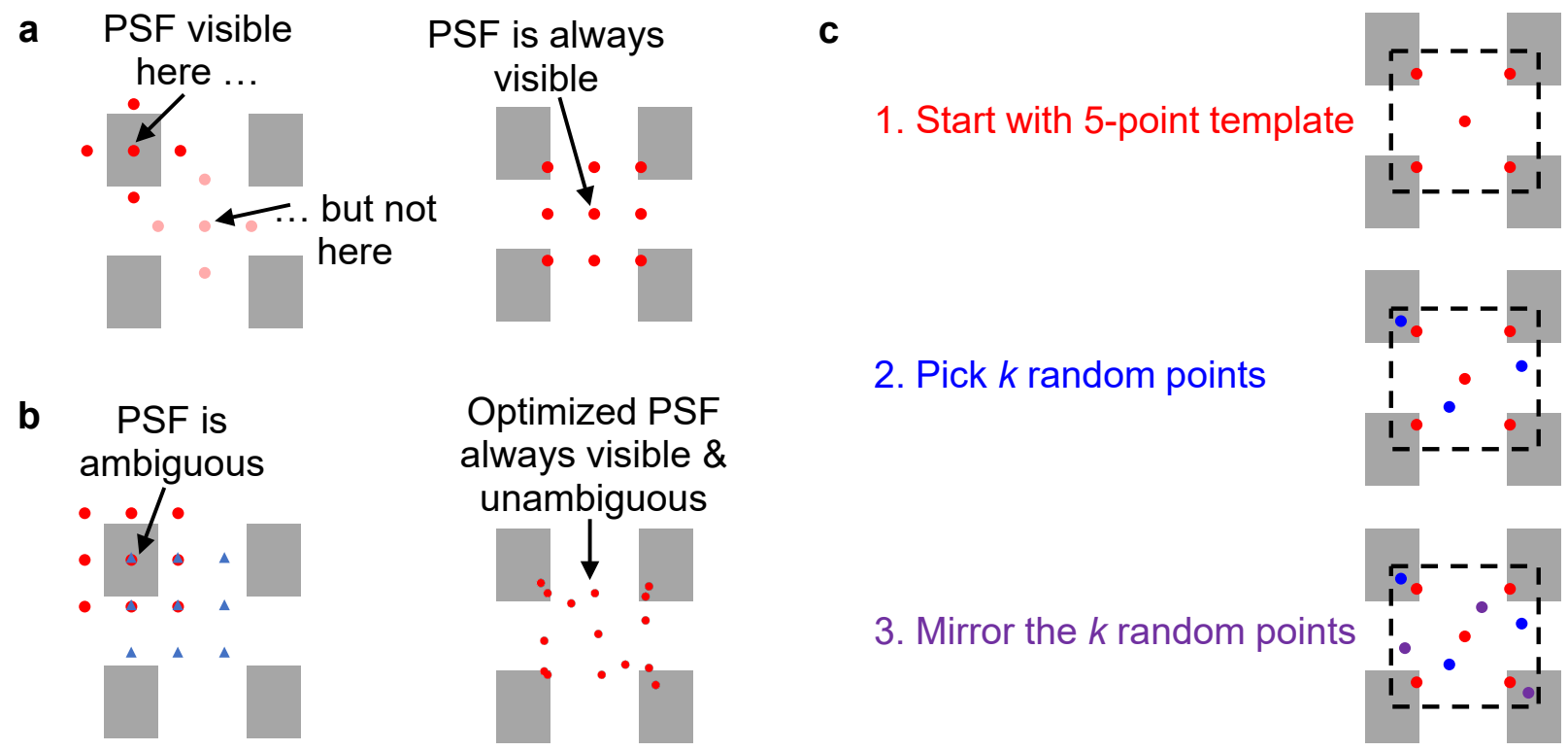

Fig. S1. (a) The PSF should be visible on all sensors (gray rectangles), regardless of its position. While a 5-point PSF arranged in a "+" is not visible everywhere, a 3×3 grid PSF slightly larger than the largest inter-sensor gap is visible everywhere. (b) The PSF should be minimally ambiguous with translation. While the 3×3 PSF is visible everywhere, it produces translational ambiguities. The optimized PSF has no strict ambiguities. (c) The three steps in proposing a candidate PSF in the PSF search algorithm.

PSF produced by the following procedure is not optimal by any metric, but rather is sufficient in practice for our imaging system.

Step 1: Propose candidate PSF. We specified a number of points, k, the PSF can have, and a box bounding the PSF points, which we set to 1.3 times larger than the largest rectangular inter-sensor gap. We found that uniform random proposals overwhelmingly generated PSFs that failed the pan-visibility and minimal ambiguity criteria. Instead, we employed two strategies that increased the probability of generating a good PSF: 1) using a fixed five-point template just barely larger than the largest rectangular gap (Fig. S1c), and 2) proposing PSFs with inversion symmetry (we break the symmetry later – see step 5 below). Due to this symmetry plus the zero order spot, k is always odd.

Step 2: Check adherence to pan-visibility criterion This step was fast, and screened out many candidates, before computing the triple correlation in the next step, which was much slower.

Step 3: Compute triple correlation efficiently and identify shift ambiguities. Since triple-correlating three 2D arrays (6D total) would be very slow, especially given the sizes of the arrays, we instead exploited a shortcut possible due to the multi-impulse parameterization of the PSF. Specifically, we considered Eq. S2 as a modulated cross-correlation between $psf(\mathbf{r})$ and a windowed version, $psf(\mathbf{r})Mask(\mathbf{r}+\Delta\mathbf{r}')$, where the former is a collection of k points, $\Delta\mathbf{r}_i$, and the latter is a collection of $k' \leq k$ points, $\Delta\mathbf{r}_j$. Rather than create two 2D arrays to cross-correlate, we computed the pairwise 2D vectors, $\Delta\Delta\mathbf{r}_{ij} = \Delta\mathbf{r}_i - \Delta\mathbf{r}_j$, and counted the number of unique $\Delta\Delta\mathbf{r}_{ij}$ vectors. Note, then, that the computational complexity of the 2D cross-correlation scales like $k'k$ (where $k = 15$ for our final PSF), rather than with the number of pixels in the extended PSF.

After counting the unique $\Delta\Delta\mathbf{r}_{ij}$'s, we identified the maximum number of occurrences of any one $\Delta\Delta\mathbf{r}_{ij}$ (in other words, the number of points overlapping when the PSF and its modulated copy were offset by a shift of $\Delta\Delta\mathbf{r}_{ij}$) – if this number was equal to k' and there were at least two different $\Delta\Delta\mathbf{r}_{ij}$'s that occurred k' times (one of them being $\Delta\Delta\mathbf{r}_{ij} = (0,0)$), then each additional $\Delta\Delta\mathbf{r}_{ij}$ besides $(0,0)$ was a potential 2D shift that produced an ambiguity in the proposed PSF. Put another way, this condition meant that all k' points of the modulated PSF fully overlapped with the k-point PSF at at least two unique relative shifts (one of them being zero shift). We then

tested for the only situation that this was not an ambiguity, which was if more than k' points of the full PSF shifted by $\Delta\Delta\mathbf{r}_{ij}$ were seen by the sensor array – the additional points break the ambiguity.

We must still repeat step 3 for all $\Delta\mathbf{r}'$ – that is, shifting the PSF to different parts of the sensor array (which incidentally also may change k').

Step 4: Save the PSF and compute its scale robustness. Many candidate PSFs based on our sampling heuristic make it this far. We differentiate these candidate PSFs based on how robust they are to scaling errors. In particular, we isotropically scale the PSF by factors between $s = 0.8$ and 1.2 and rerun steps 2 and 3. Supposing that scaling the PSF by any s value within interval of $[s_{min}, s_{max}]$ passes steps 2 and 3, we report s_{max}/s_{min} as the PSF's scale robustness factor. After running steps 1-3 many times and producing many candidate PSFs, we picked the one with the highest scale robustness factor.

Step 5: Break PSF symmetry. We previously enforced inversion symmetry in the proposed PSFs to decrease the rejection rate. However, symmetric PSFs lead to larger autocorrelations, as discussed in Sec. S1.1. Essentially, if one picks any two non-opposite PSF points, $\Delta\mathbf{r}_i$ and $\Delta\mathbf{r}_j$, they will simultaneously overlap with $-\Delta\mathbf{r}_j$ and $-\Delta\mathbf{r}_i$, respectively, leading to autocorrelation peaks with amplitude $2/k$. There are (k choose 2) $-$ $(k-1)/2$ of such autocorrelation peaks (the second term arises due to the fact that picking opposite PSF points, $\Delta\mathbf{r}_i$ and $-\Delta\mathbf{r}_i$, does not lead to overlapping).

Thus, in this step, we perturb the PSF point coordinates to break the symmetry, allowing the max autocorrelation of the PSF to be $1/k$. In other words, we wanted to split the $2/k$-amplitude peaks into pairs of $1/k$-amplitude peaks, so that the split distance was as long as possible. If we pose this problem as a maximization of the minimum possible split distance among all the splits, it becomes equivalent to a circle packing problem (for $(k-1)/2$ circles). Since there's no guarantee that the perturbed PSF preserves the scale robustness factor, and since there's no inherent ordering of the $(k-1)/2$ circles, we randomly permuted the 2D perturbation vectors and picked the order that maximized the scale robustness factor.

Step 6: Repeat the above procedure for other values of k. We wanted k to be as small as possible to minimize the number of pixels into which the light would be distributed, but large enough that the PSF satisfies the pan-visibility and minimal ambiguity conditions with a large scale robustness factor. We empirically found that $k = 13$ was sufficient, but yielded low scale robustness factors (<1.05), so we opted for $k = 15$ to obtain larger robustness factors (~1.15).

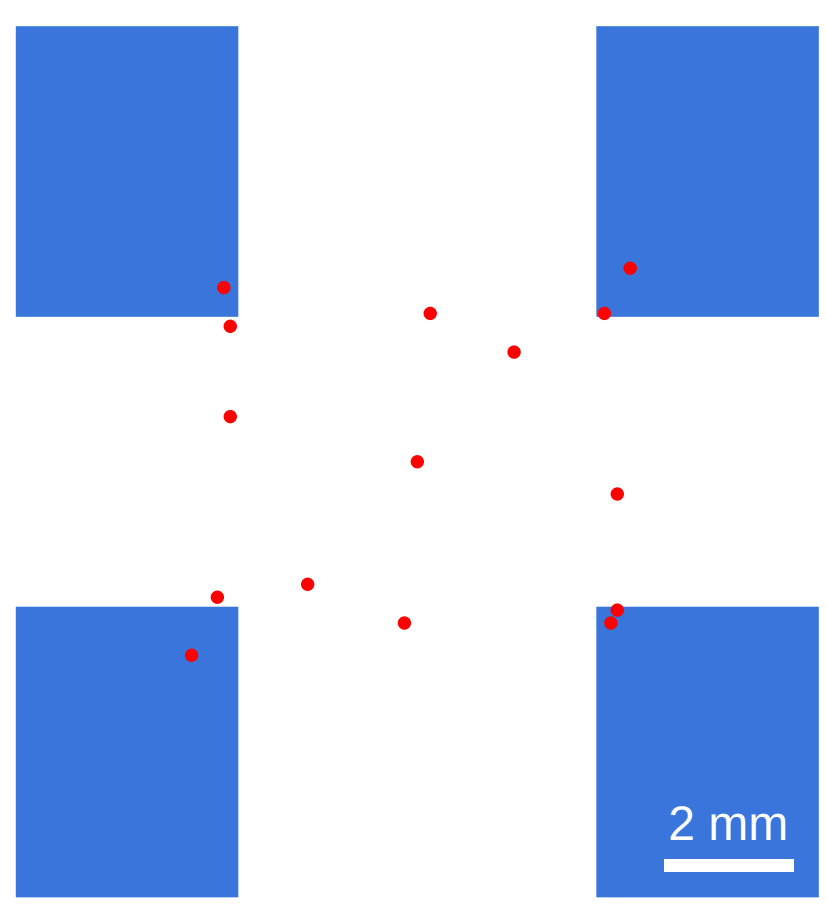

Fig. S2. Optimized PSF, positioned such that the zero order is at the center of the gap of a 2×2 sensor subarray. The individual PSF impulses are enlarged so that they are visible.

S1.3. Final PSF design

The final PSF design is shown in Fig. S2 and its impulse coordinates are listed in Table S1.

x (mm)	3.3	3.0	3.1	1.5	-0.2	3.1	2.9	-3.5	-3.0	-2.9	-1.7	0.2	-2.9	-3.1	0
y (mm)	3.0	-2.5	-0.5	1.7	-2.5	-2.3	2.3	-3.0	2.7	0.7	-1.9	2.3	2.1	-2.1	0

Table S1. The optimized PSF coordinates, relative to the zero order (the last coordinate).

S2. Field of view extrapolation

The large spatial extent of the PSF on the sensor (6 mm × 6.8 mm) enables extrapolation beyond the native rectangular hull of the sensor array (4.95 cm × 6.64 cm). If we require half of the width or height of the PSF to be within the native rectangular hull, then the extrapolated FOV is simply the sum of the corresponding dimensions: 5.63 cm × 7.24 cm. Here, we validate that we can obtain good reconstruction quality in this extrapolation region by imaging a 2D target, translated well beyond the edge of the extrapolated FOV (Fig. S3).

S3. Comparison of shift-invariant and shift-variant model

In the forward model described in Sec. 2.2, we introduced a convolutional forward model, interpreting it as an incoherent superposition of multiple shifted copies of the object without explicitly using convolution. This interpretation also allows full modeling of the distortion-induced spatial variance of the PSF. In this section, we justify this "direct" approach by demonstrating that it not only leads to more accurate reconstructions, but also uses the least memory and does not significantly trade-off speed compared to FFT-based approaches. In particular, here, we compare three algorithms: full-FFT, patch-FFT, and direct:

Full-FFT: a shift-invariant model utilizing FFT-based convolution, as described in Eq. 2. To achieve effective interpolation of the PSF's FFT, we pad the PSF to match the reconstruction size and then perform matrix multiplication in the frequency domain.

Patch-FFT: a patch-wise shift-variant model using FFT-based convolution. The FOV is divided into patches corresponding to the sensor array (e.g., 6×8 patches). For each sensor patch, we determined a support region encompassing all potential object-space points contributing to that sensor patch. The size of this region is the sum of the sensor size and the PSF size. In the forward model, we employ a sliding window to extract patches based on this support, apply FFT-based convolution with each patch's corresponding PSF, and crop the resulting sensor region to predict the measurement.

Direct: a fully shift-variant model utilizing direct superposition, as formulated in Eq. 7. This approach implements a direct convolution, where the PSF is approximated as delta functions, with all zero-multiplication operations eliminated for computational efficiency.

In Fig. S4, we use a lithography target as the sample and compare the algorithms' reconstruction against the ground truth captured by a regular microscope with 1x maginification. The algorithms' time and memory performance metrics are evaluated using the JAX framework with its just-in-time (JIT) compilation, executed on a single NVIDIA RTX 3090 GPU. We apply 8x downsampling to the measurement for all the methods, resulting in a reconstruction with dimensions of 6.3k×8.3k (52 MP). The analysis includes two key performance indicators: the total computation time for 100 optimization iterations and the peak memory usage. These metrics are displayed in the bottom right corner of the figure, where the top row indicates the time consumption, and the bottom row shows the peak memory usage as reported by the memory_stats field of the JAX device.

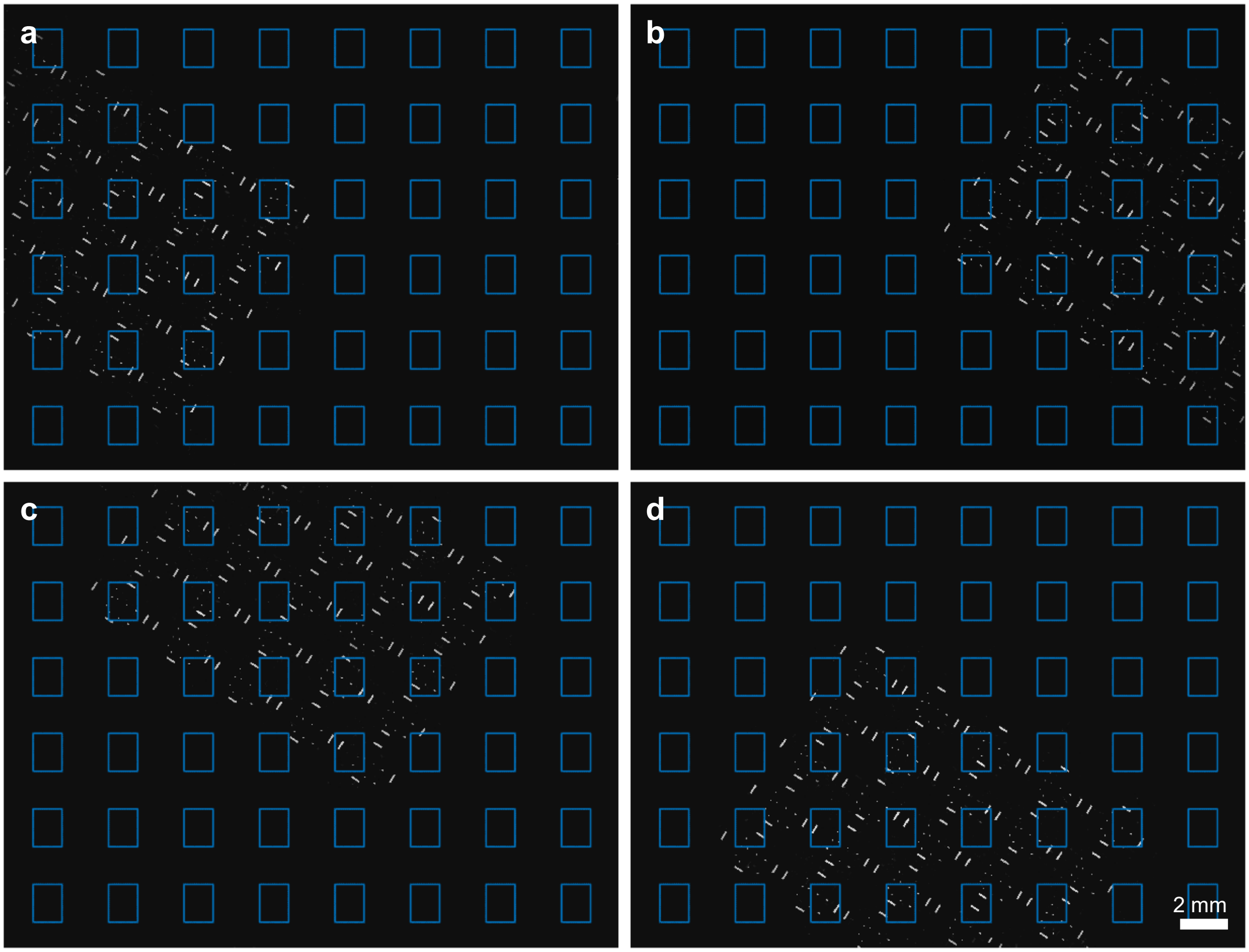

Fig. S3. Our method enables extrapolation beyond the rectangular hull of the sensor array (i.e., the smallest box bounding all 48 sensors, denoted by blue boxes). This figure shows the same target as in Fig. 2b translated and reconstructed beyond the left (a), right (b), top (c), and bottom (d) edges.

The direct approach yields the best reconstruction quality, followed by the patch-FFT and full-FFT approaches. When PSF shift variance is not properly accounted for (as in the full-FFT and patch-FFT method), the reconstruction suffers from either extra artifacts or eliminated objects. This trade-off underscores the importance of accurately modeling the shift-variant PSF distortion.

We notice that the improvement in quality from patch-FFT over full-FFT is relatively minor. This is possibly because the patches are divided uniformly across the FOV, while the shift variance is not uniformly distributed for a lens system. It is optimized to have relatively small distortions in the central region, whereas the peripheral region exhibits more drastic PSF variations. We also observe that even with the direct method, the squares in the corner regions (especially in the bottom left corner) are not well reconstructed. This degradation primarily stems from lens vignetting, which naturally attenuates light intensity in peripheral regions.

To ensure a fair comparison, we generated the central and patch-wise PSFs for both FFT methods using lens parameters co-optimized according to Sec. 2.2. Specifically, we jointly optimize the 2D object $Obj(\mathrm{r})$ and the lens distortion parameters $\{a_i^{tube}\}_{i=1}^{m}$, $\{a_i^{obj}\}_{i=1}^{m}$ for both the tube lens and objective. The optimized lens parameters are then used to generate PSFs at

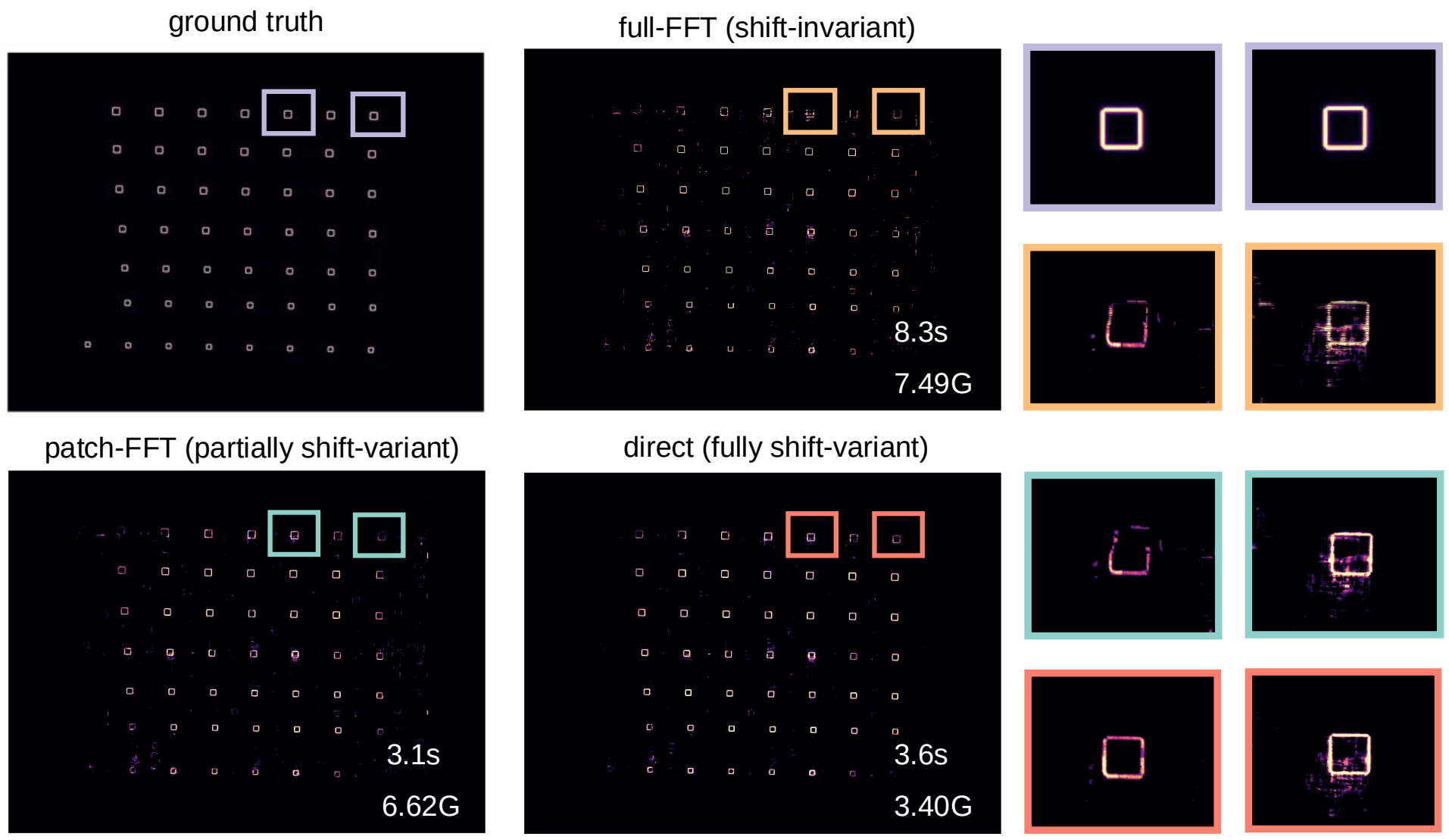

Fig. S4. Performance comparison across different algorithms: full-FFT, patch-FFT, and direct. Despite of being slightly slower than patch-FFT, the direct method achieves the best reconstruction quality, particularly in the peripheral regions. Additionally, the direct method consumes the least memory, making it suitable for full-resolution reconstruction without downsampling.

the center of the full reconstruction and at each sensor's center for the full-FFT and patch-FFT methods, respectively. For the direct method evaluation, we disable lens parameter optimization since these parameters need only be co-optimized once.

S4. Radial spectral blurring: SNR-resolution tradeoff

The wavelength dependence of DOEs can affect the spatial resolution of our computational microscope. In particular, the size of the PSF, as the far-field diffraction pattern of the DOE, is scaled in proportion to the wavelength:

$$psf(\mathbf{r}, \lambda) = psf\left(\frac{\lambda}{\lambda_0}\mathbf{r}, \lambda_0\right), \tag{S4}$$

where $\mathbf{r} = (x, y)$ is the 2D spatial coordinate in the image plane, λ_0 is the design wavelength, and λ is an arbitrary wavelength. The overall broadband PSF is thus

$$psf_{P(\lambda)}(\mathbf{r}) = \int_0^\infty P(\lambda) psf(\mathbf{r}, \lambda) d\lambda, \tag{S5}$$

where $P(\lambda)$ is the power spectral density of the detected light. As a result, the individual impulses of the PSF experience radial blurring, δr, in proportion to the bandwidth of the source, $\Delta\lambda$, and the radial distance from the zero order impulse,

$$\delta r \propto \frac{\Delta\lambda}{\lambda_0}|\mathbf{r}|, \tag{S6}$$

suggesting that the PSF should be as small as possible and that the bandwidth of the detected light be as narrow as possible. The tolerance to bandwidth and PSF size depends on how δr

compares to the size of each PSF impulse, which in turn is determined by the tube lens NA and system aberrations. Assuming an impulse spot size of 8.8 µm (the size that is critically sampled in the 4×4 superpixel, with a pixel size of 1.1 µm), a maximum radius of 3.5709 mm (given an inter-sensor gap size of 5.5504 mm by 4.4944 mm), and a design wavelength of 515 nm, the bandwidth that produces a radial blur of 8.8 µm is $\Delta\lambda \approx 1.3$ nm. Thus, we used a 2-nm bandpass filter for the darkfield imaging mode as a compromise between resolution and light throughput.

For the fluorescence imaging mode, it would have been unacceptable to impose a 1-2-nm bandpass filter, given how weak fluorescence signals tend to be. Instead, we used a 55-nm bandpass filter and took advantage of the chromatic aberrations in the objective lens. Thus, when the wavelength deviates from the design wavelength, λ_0, we not only get scaling, but also defocus. We can modify Eq. S4 to incorporate blurring due to defocus:

$$psf(\mathbf{r}, \lambda) = psf\left(\frac{\lambda}{\lambda_0}\mathbf{r}, \lambda_0\right) \otimes defocus\left(\mathbf{r}, \frac{df}{d\lambda}(\lambda - \lambda_0)\right), \tag{S7}$$

where $defocus(\mathbf{r}, dz)$ is a 2D blur kernel due to a defocus of dz and $df/d\lambda$ describes the focal shift per wavelength shift governing the axial chromatic aberration.

Applying Eq. S5 results in a PSF that has a sharp in-focus component corresponding to the power at wavelengths close to the nominal wavelength, λ_0, and heavy tails due to the defocused components corresponding to the other wavelength components. With sufficient axial chromatic aberration, the lateral resolution is less impacted – this principle has been previously applied for extended DOF imaging [1]. However, there is still a tradeoff between total light throughput and background signal when spectrally filtering the fluorescence emission, with SNR being the ultimate metric of interest. That is, while increasing total light throughput improves SNR, increasing the background, although it can be digitally subtracted, introduces shot noise – the total noise is a quadrature sum of the signal shot noise, the background shot noise, and the detector noise. Empirically, we found that the signal enhancement from increased light throughput outweighed the deleterious effects of background.

We have not included the effects of spectrally-dependent diffraction efficiency of the DOE in Eqs. S4-S7, which increases the power in the zero order as the wavelength deviates from λ_0, modifying the effective $P(\lambda)$ for the nonzero orders. This effect could be compensated for in future designs by decreasing the zero order contribution at the λ_0, based on the expected fluorescence emission bandwidth. However, in practice, we found that the spectrally-dependent diffraction efficiency over the 55-nm bandwidth had minimal impact.

S5. L1 hyperparameter sweep

Fig. S5 demonstrates how reconstruction MSE varies with object density and L1 regularization strength. Here, density is defined as the percentage of non-zero points within the reconstructed FOV. For dense objects, weaker sparsity constraints (lower L1 regularization) yield better results. In contrast, relatively sparse objects exhibit an optimal L1 regularization strength, with performance degrading when the regularization is either too weak or too strong.

These results were obtained through simulated measurements. We generated ground truth samples with randomly scattered dots at specified densities and simulate the measurements through the forward model. The analysis employed the direct method detailed in Sec. S3, assuming accurately known lens parameters.

S6. Impact of sample sparsity on reconstruction quality

To quantify the effect of sample sparsity on reconstruction fidelity, we conducted an empirical study using a programmable target. Sparsity is quantified by the sample density, d, defined as the fraction of non-zero pixels across the object field-of-view (FOV). We utilized the darkfield

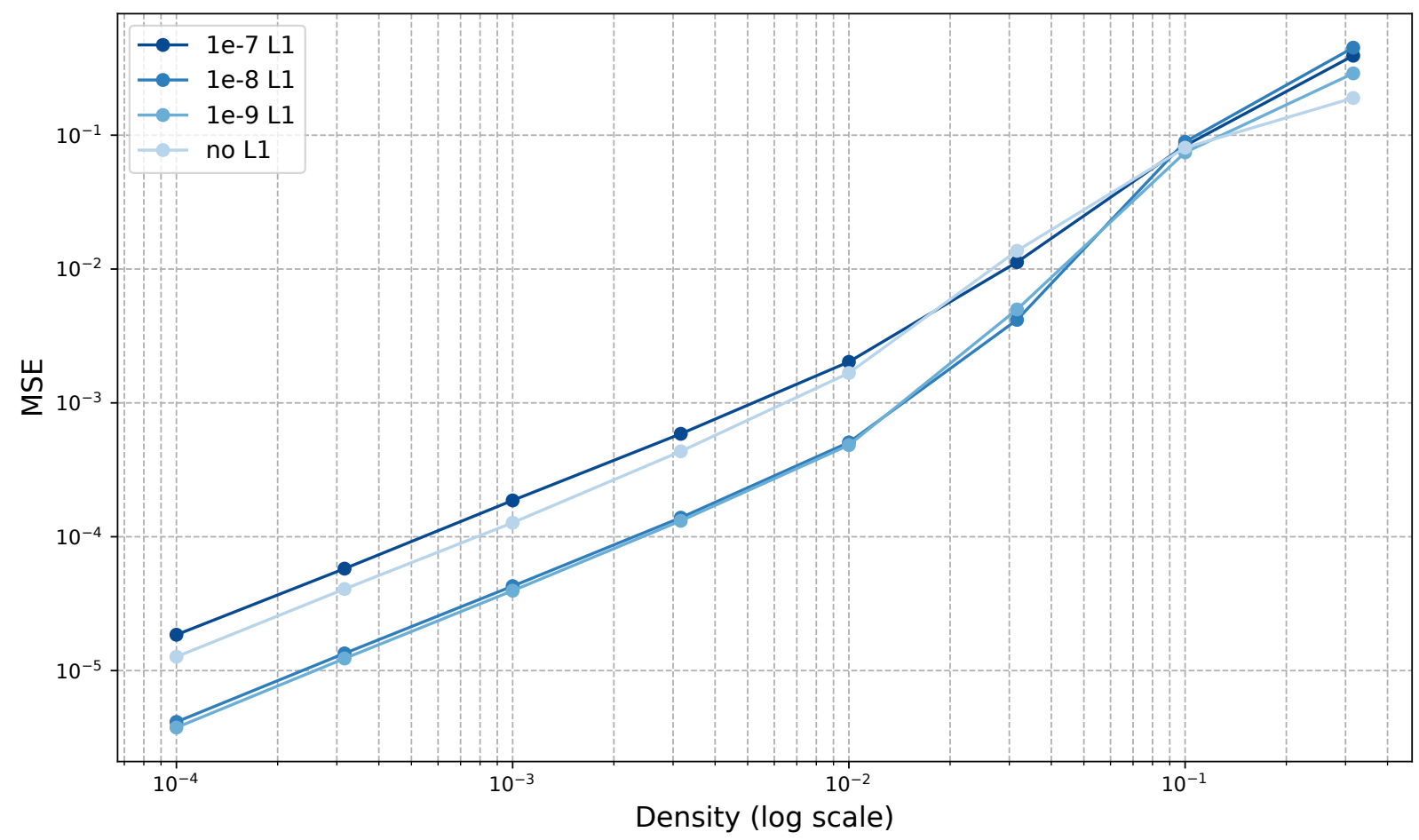

Fig. S5. Reconstruction MSE across different ground truth object density with different L1 regularization strength.

imaging configuration, which consists of an FJW Industries objective (f = 90 mm), a 514/2 nm narrowband filter, and a 40-mm-diameter aperture (though note that we did not perform darkfield illumination). To provide a controllable ground truth (GT) across the full FOV, we employed an iPhone 17 Pro screen as the imaging target, generating synthetic patterns consisting of randomly scattered green dots (corresponding to ~20 µm OLED pixels).

The qualitative results, illustrated in Fig. S6, reveal an empirical threshold for successful image recovery. At a high density ($d > 10^{-2}$), the system is unable to achieve a high-fidelity reconstruction. Regardless of the ℓ_1 regularization weight (λ) applied, the algorithm either fails to reconstruct certain points (false negatives, appearing as red in the overlay) or introduces artifacts (false positives, appearing as green). Conversely, when the sample is sufficiently sparse ($d \sim 10^{-2}$ or lower), artifact-free recovery is readily achieved using an optimal sparsity regularization weight. In these highly sparse regimes, the reconstruction and ground truth are nearly perfectly coincident, appearing as white.

To benchmark our reconstruction performance across varying target densities, we quantitatively evaluated both the continuous pixel intensities and the binary recovery of the dots displayed on the screen in Fig. S7. Prior to computing performance metrics, both the reconstructed images and the ground truth were downsampled by 8× for computational efficiency and smoothed with a Gaussian kernel (σ = 10 pixels). This blurring step accommodates small residual distortion, ensuring the evaluation focuses on successful target recovery. First, we measured the mean squared error (MSE) between these preprocessed images to quantify overall intensity accuracy. Second, we treated the dot recovery as a pixel-wise classification problem. By applying varying intensity thresholds, we generated receiver operating characteristic (ROC) curves and calculated the area under the curve (AUC) for each density level. An AUC of 1.0 indicates a perfect reconstruction, while 0.5 represents a random guess. As demonstrated across multiple regularization weights (λ), reconstruction fidelity remains high for target densities below 10^{-2}. Beyond this density, the MSE steadily increases and the AUC experiences a relatively sharp drop towards 0.5. Note that while we still can perform reconstruction at higher densities, there is a trade-off between false negatives and false positives.

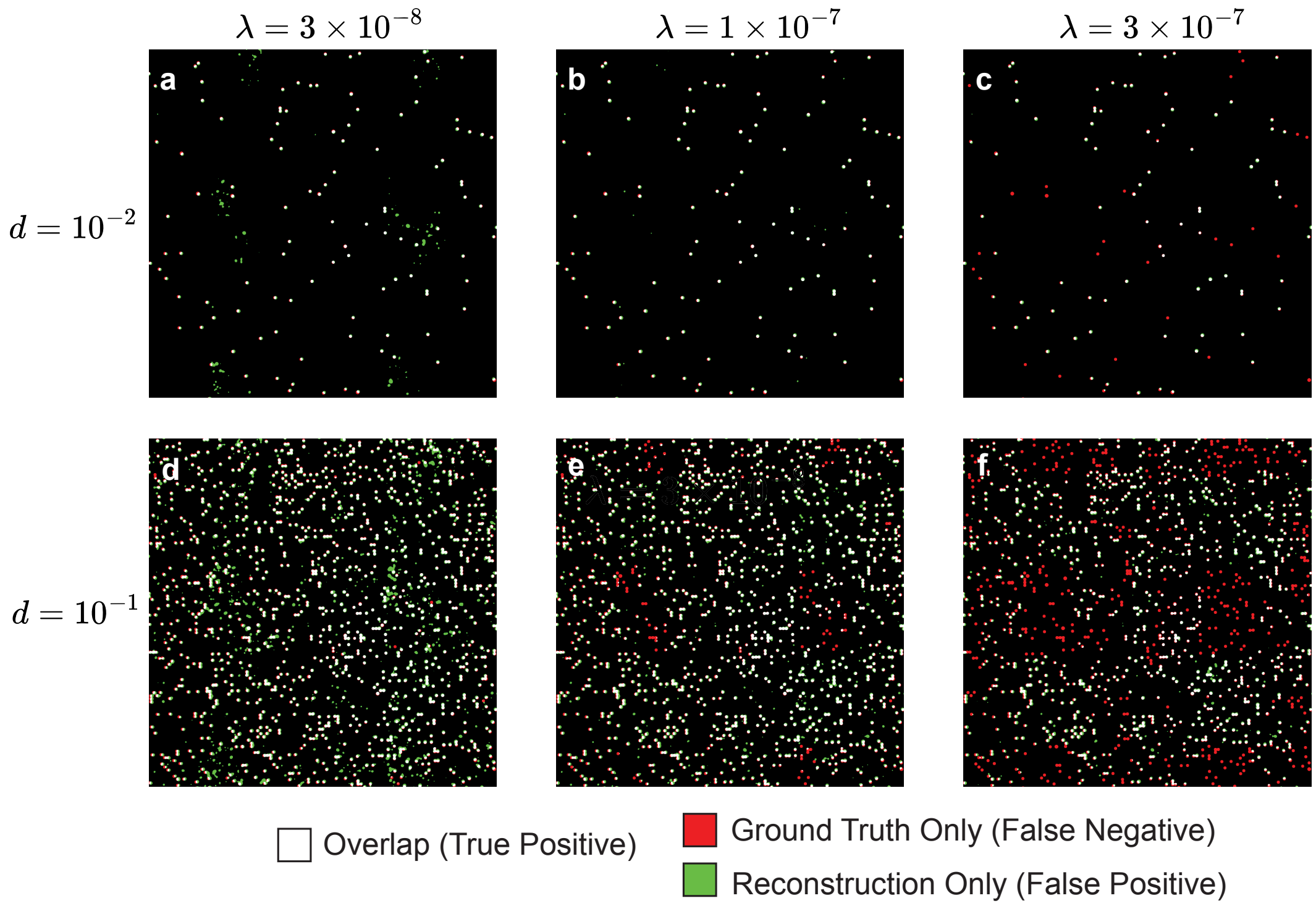

Fig. S6. Experimental evaluation of reconstruction fidelity versus object density. Binarized overlays (threshold 0.1) comparing reconstructions across varying ℓ_1 regularization strengths (λ). White indicates accurate recovery (true positive), red indicates missed ground truth (false negative), and green indicates artifacts (false positive). (a-c) Reconstructions at a lower object density ($d = 10^{-2}$), showing near-perfect overlap at the optimal λ. (d-f) Reconstructions at a higher object density ($d = 10^{-1}$), exhibiting increased false positives and false negatives across all evaluated λ values. All panels display a representative $\sim 6.3 \times 6.3$ mm^2 physical region cropped from the center of the full multi-centimeter field-of-view.

S7. Validating the separable distortion model

Fig. S8 illustrates the separable distortion model described in Sec. 2.2. To experimentally validate this distortion model and calibrate the system, we measured the PSF using a 2 μm pinhole illuminated by an LED as a point source. Because our extended, 15-spot PSF is specifically designed to span the inter-sensor gaps to enable multiplexing, a single-shot measurement of the complete PSF is physically impossible, as any single capture will always be partially cropped by the sensor boundaries. To overcome this, we assumed that the PSF does not change significantly over small displacements. By shifting the point source to 2×3 nearby locations using the motorized stage, we were able to capture all 15 spots within the boundaries of a single sensor and stitch them together to form the complete experimental PSF.

Fig. S9 shows the experimental PSF (at both the center and edge of the FOV) and validates our distortion model. In the left column, the stitched experimental PSF is overlaid with the designed PSF, illustrating the initial mismatch caused by rotational misalignment during DOE mounting and the distortions caused by the lenses. In the right column, the same stitched experimental PSF is overlaid with our computationally parameterized PSF following the co-optimization of

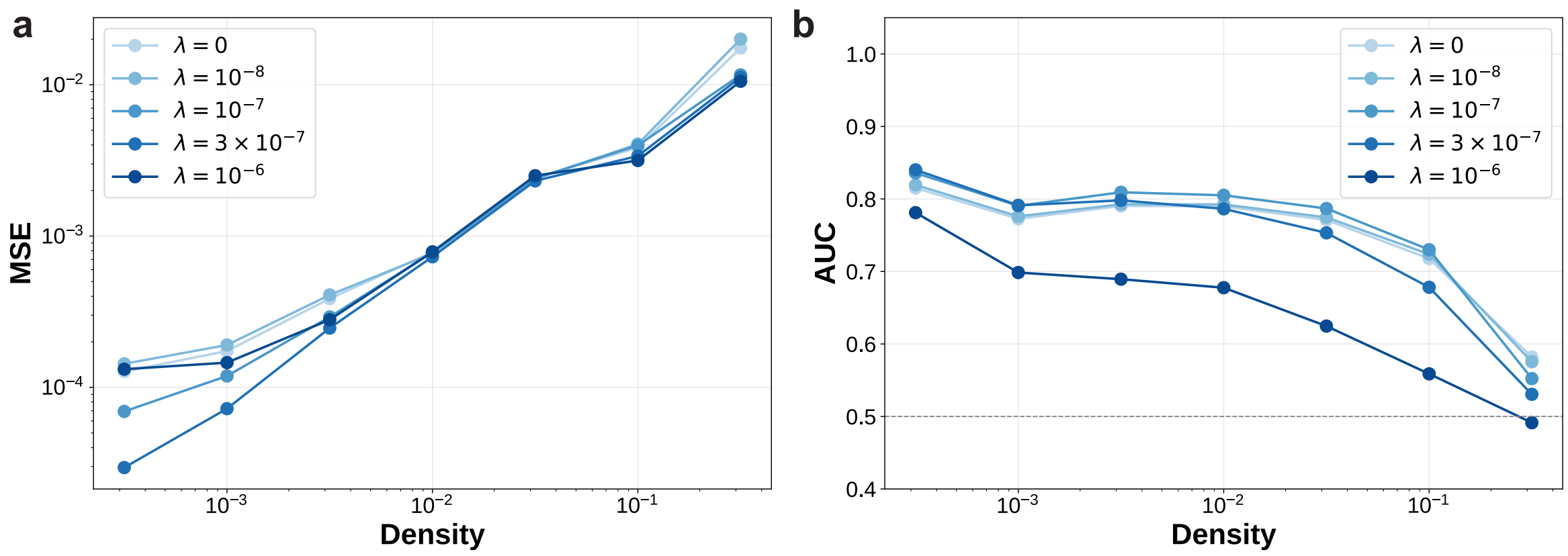

Fig. S7. Quantitative evaluation of reconstruction fidelity across varying target densities. (a) Mean squared error (MSE) between the reconstructed continuous intensities and the ground truth. (b) Area under the curve (AUC) for the pixel-wise classification of binary target dots. Performance is plotted across multiple L_1 regularization parameters (λ) to illustrate the optimization trade-offs. The dashed gray line at 0.5 represents the baseline performance of a random classifier.

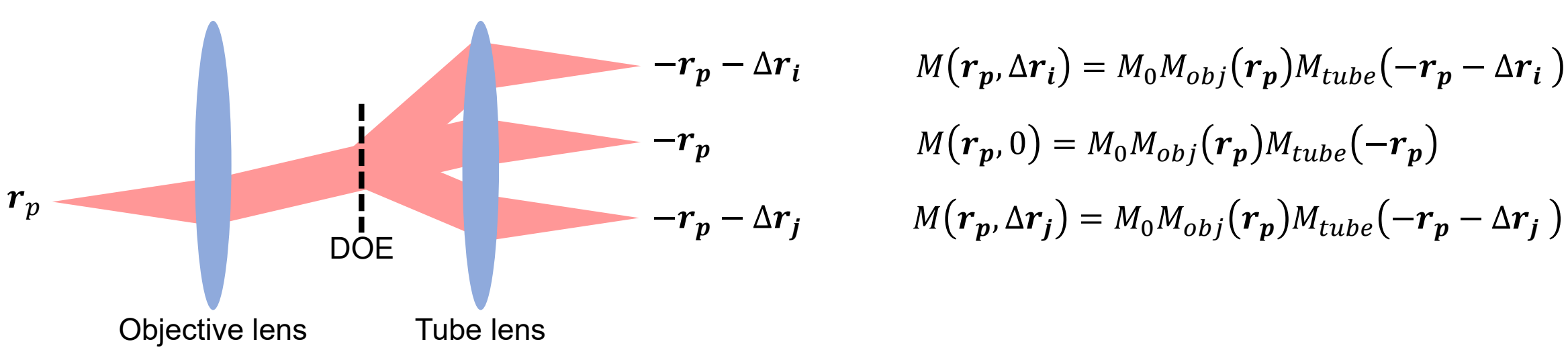

Fig. S8. We model the spatially-varying PSF of our imaging system based on a separable radial distortion model, whereby the objective and tube lens each has its own radial distortion model (i.e., radially-varying magnifications: $M_{obj}(\mathbf{r})$ and $M_{tube}(\mathbf{r})$). In an ideal imaging system with unit magnification, a position at point $\mathbf{r}_p$ in the object plane is mapped to a collection of points, $-\mathbf{r}_p - \Delta\mathbf{r}_i$, comprising the PSF. The total system magnification is thus a product of the independent contributions from the objective ($M_{obj}(\mathbf{r})$), the tube lens ($M_{tube}(\mathbf{r})$), and the nominal magnification (M_0, given by the ratio of the focal lengths of the objective and tube lens).

our distortion model. As demonstrated, this co-optimization results in an improved alignment, verifying that our parameterized distortion model accurately captures the physical distortions of the system.

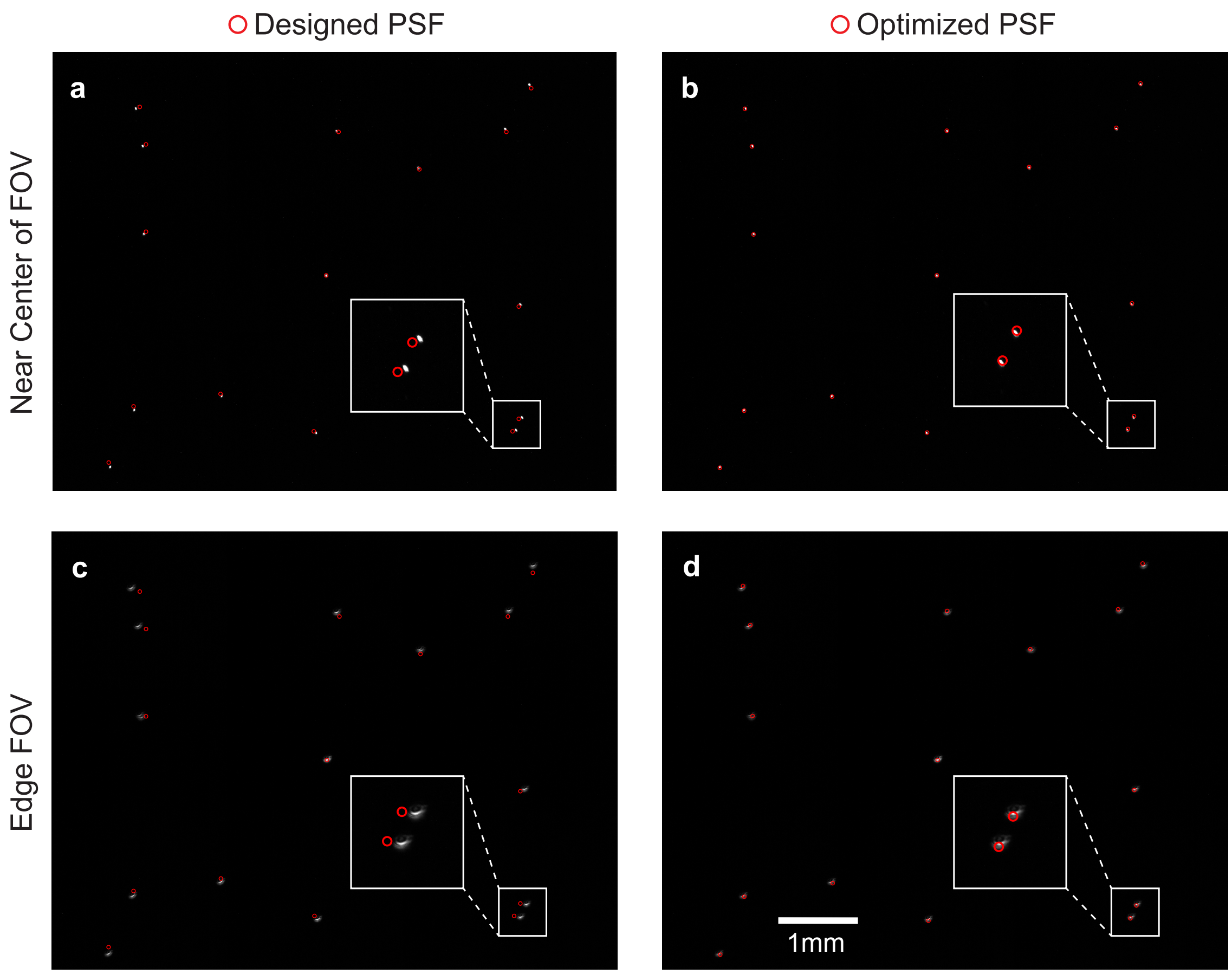

Fig. S9. Experimental validation of the computational distortion model. Stitched experimental PSFs are overlaid with (a, c) the initially designed coordinates and (b, d) the computationally parameterized coordinates after co-optimization (red symbols). The top row (a, b) displays the PSF measured near the center of the field-of-view (FOV), and the bottom row (c, d) displays the PSF at the edge of the FOV. PSFs are intentionally saturated to aid in visualization.

To show the effectiveness of our distortion model, Fig. S10 presents a qualitative comparison of the image reconstructions with and without the distortion model enabled. The left column assumes a globally shift-invariant forward model. While this baseline still optimizes for the overall rotation and scaling of the PSF (parameterized as an affine transformation of the PSF), it does not account for spatially varying lens distortions. Because of these local misalignments, there is a significant loss of image contrast in reconstruction. Conversely, the right column utilizes our fully shift-variant distortion model. Both zoom-in panels of the reconstruction show higher contrast and fewer background artifacts.

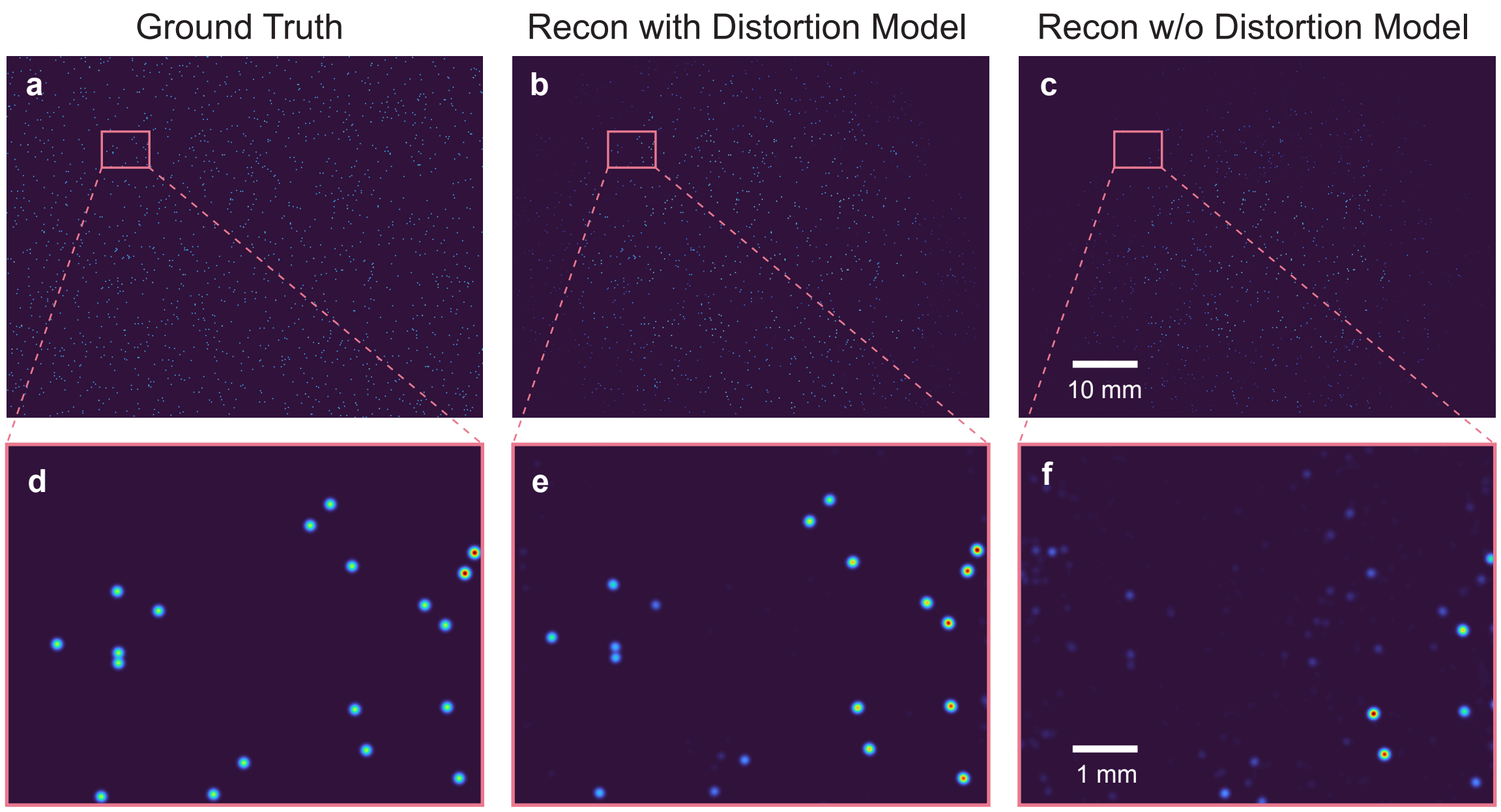

Fig. S10. Qualitative ablation study of the distortion model. Experimental data was captured in darkfield mode using a smartphone display as the target. (a, d) Ground truth target image. (b, e) Image reconstruction utilizing the fully parameterized, shift-variant distortion model. (c, f) Baseline image reconstruction without the distortion model enabled (assuming only a simple affine transformation of the PSF). To enhance the visibility of the microscopic target points across our large FOV, a Gaussian blur (σ = 10 pixels) was applied to all displayed images.

S8. Resolution, depth of field, and field curvature characterization

As described in Sec. 3, to characterize resolution, DOF, and field curvature of the two imaging configurations, we took z-stacks of a USAF target centered at each of the sensors (after magnification by the imaging system) and computed the sharpness based on the mean square image gradient (Fig. S11c,d,g,h). In most cases, we could identify the focal position via the argmax of this sharpness metric across z. However, recognizing that at certain field positions, our imaging system exhibits astigmatism, we identified the circle of least confusion as the focal position by finding the local minimum flanked by two local maxima, corresponding to the two astigmatic foci (e.g., see rightmost column of Fig. S11c). The USAF images at the best-focus positions across all sensors are plotted in Figs. S12-S15. We removed any tilt between the lateral scan trajectory and the focal plane of the objective by fitting and subtracting the plane of best fit, yielding Fig. S11a,e. Finally, we computed the DOFs based on the FWHM of the sharpness curves (Fig. S11b,f).

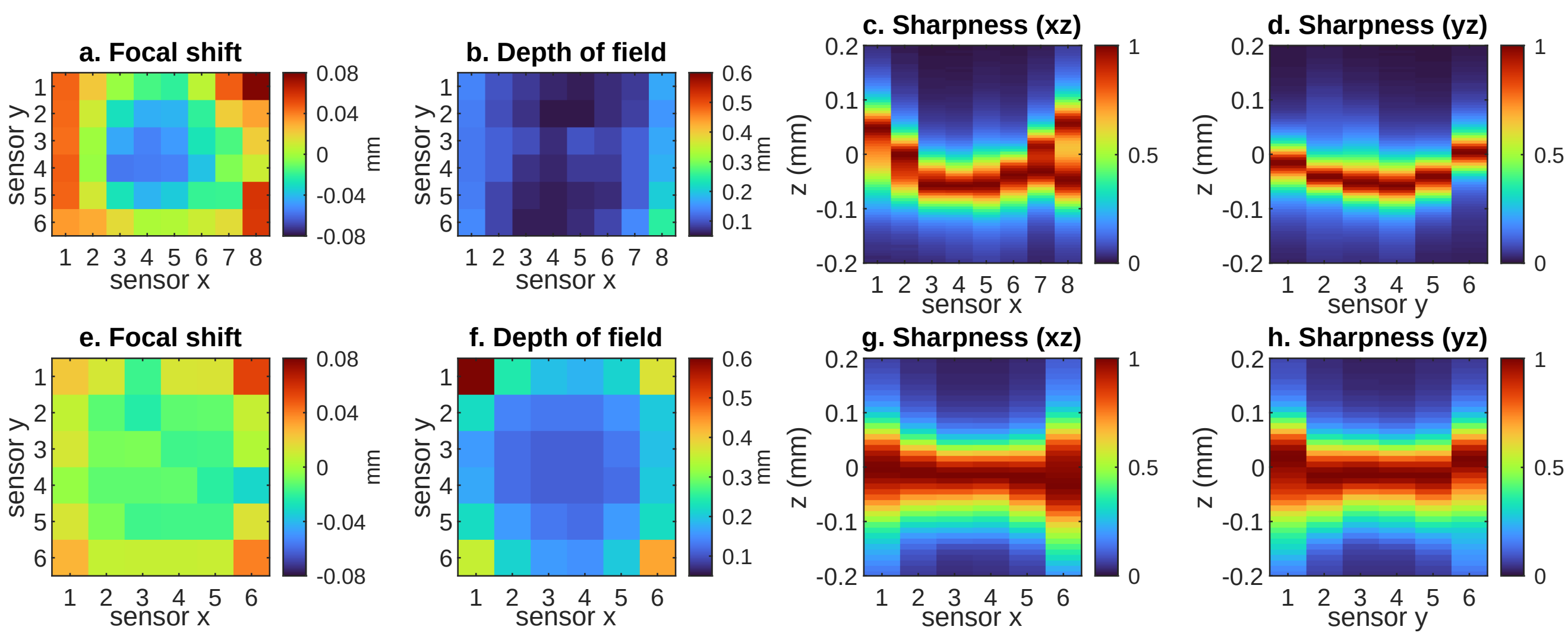

Fig. S11. Field curvature and depth of field (DOF) characterization for the darkfield (a-d) and fluorescence (e-h) imaging modes. (a,e) Focal shift at the centers of all 36 or 48 sensors. (b,f) Depth of field at the centers of all 36 or 48 sensors. (c,g) Sharpness as a function of depth and a central row of the sensor array, normalized to the max sharpness. (d,h) Normalized sharpness as a function of depth and central column of the sensor array.

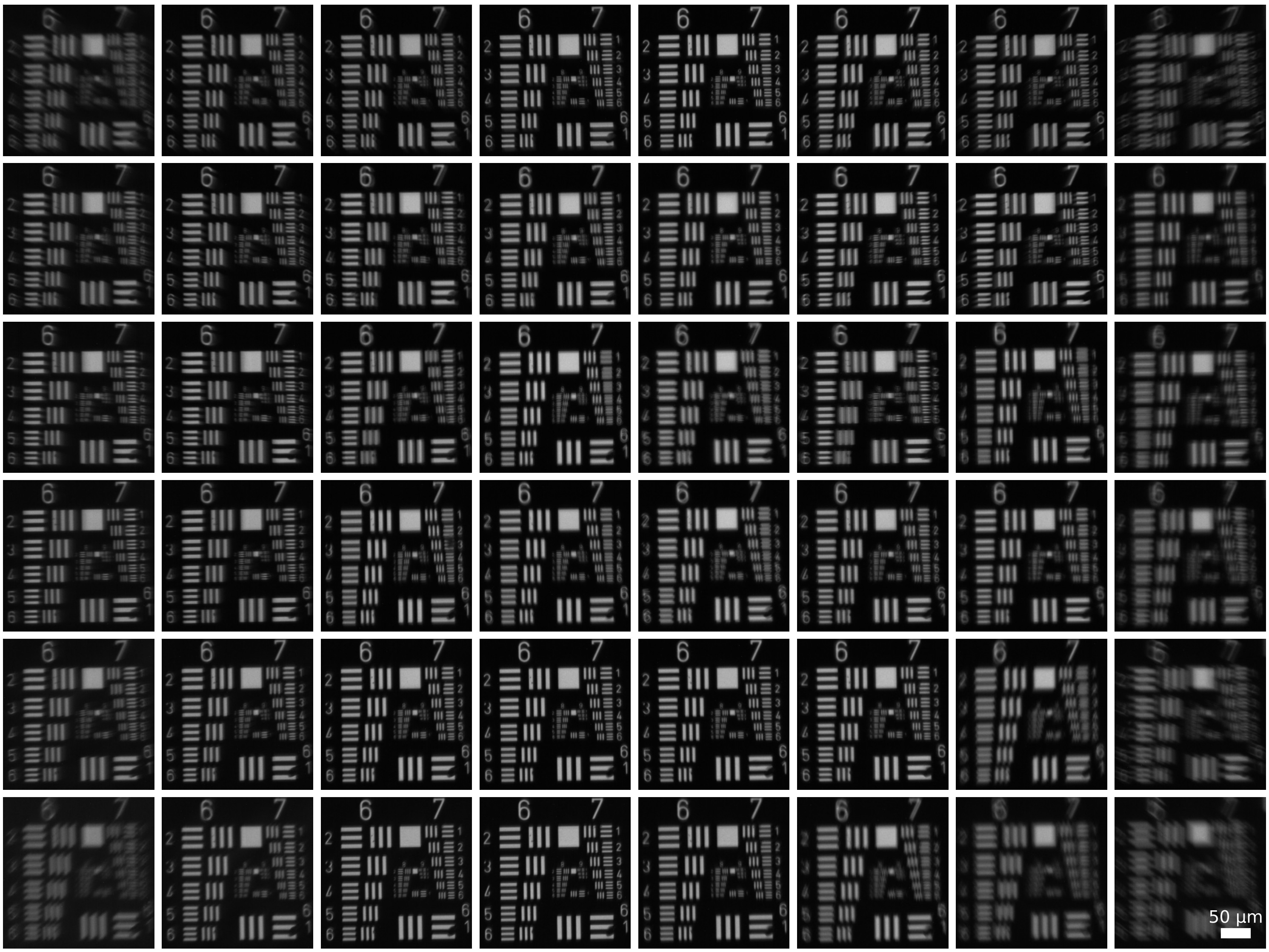

Fig. S12. Characterization of resolution across all 6 × 8 sensors in the darkfield imaging configuration. Groups 6 and higher are shown. Zoom-ins of groups 8 and 9 are shown in Fig. S13.

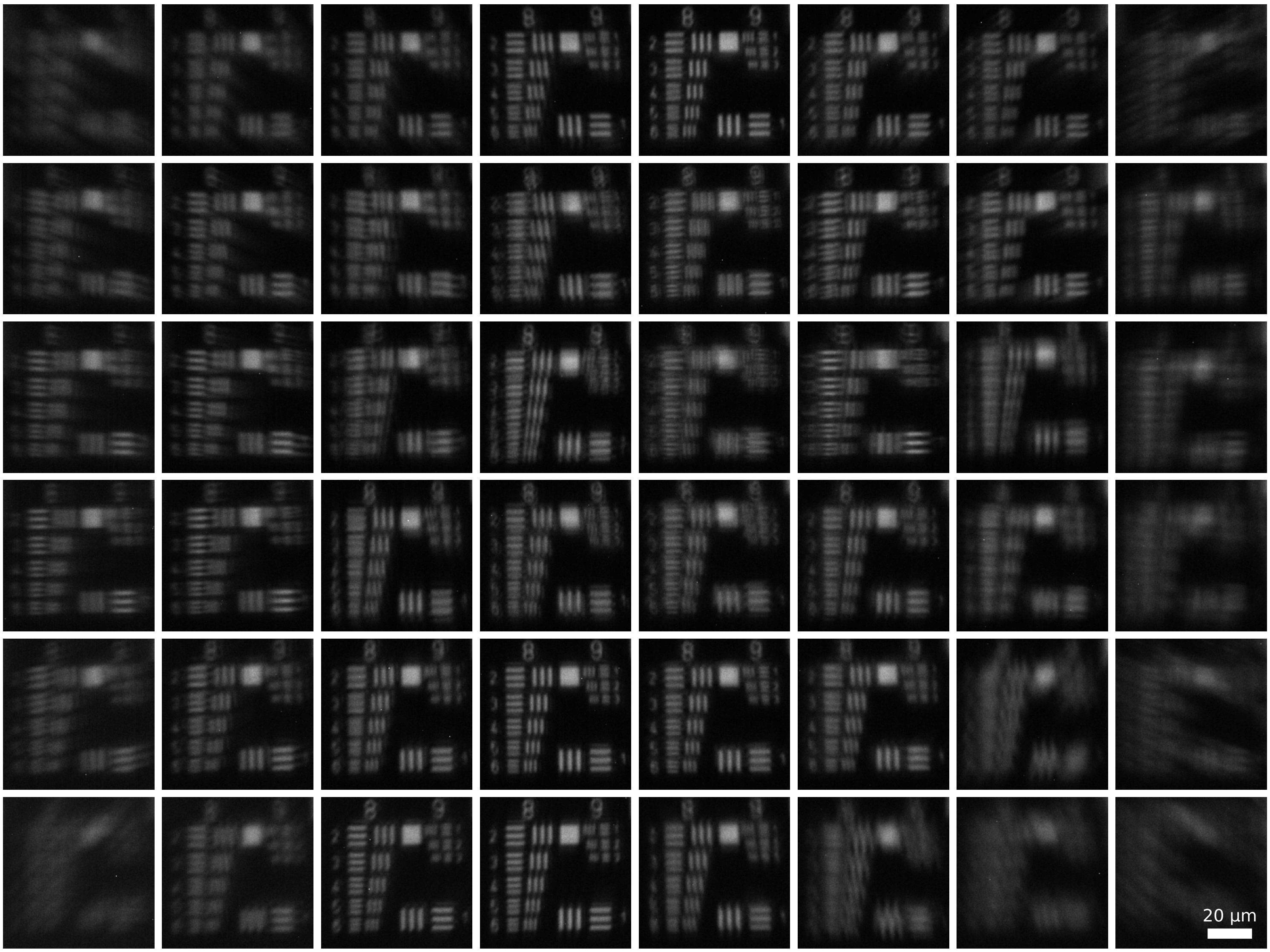

Fig. S13. Characterization of resolution across all 6 × 8 sensors in the darkfield imaging configuration. Groups 8 and 9 are shown.

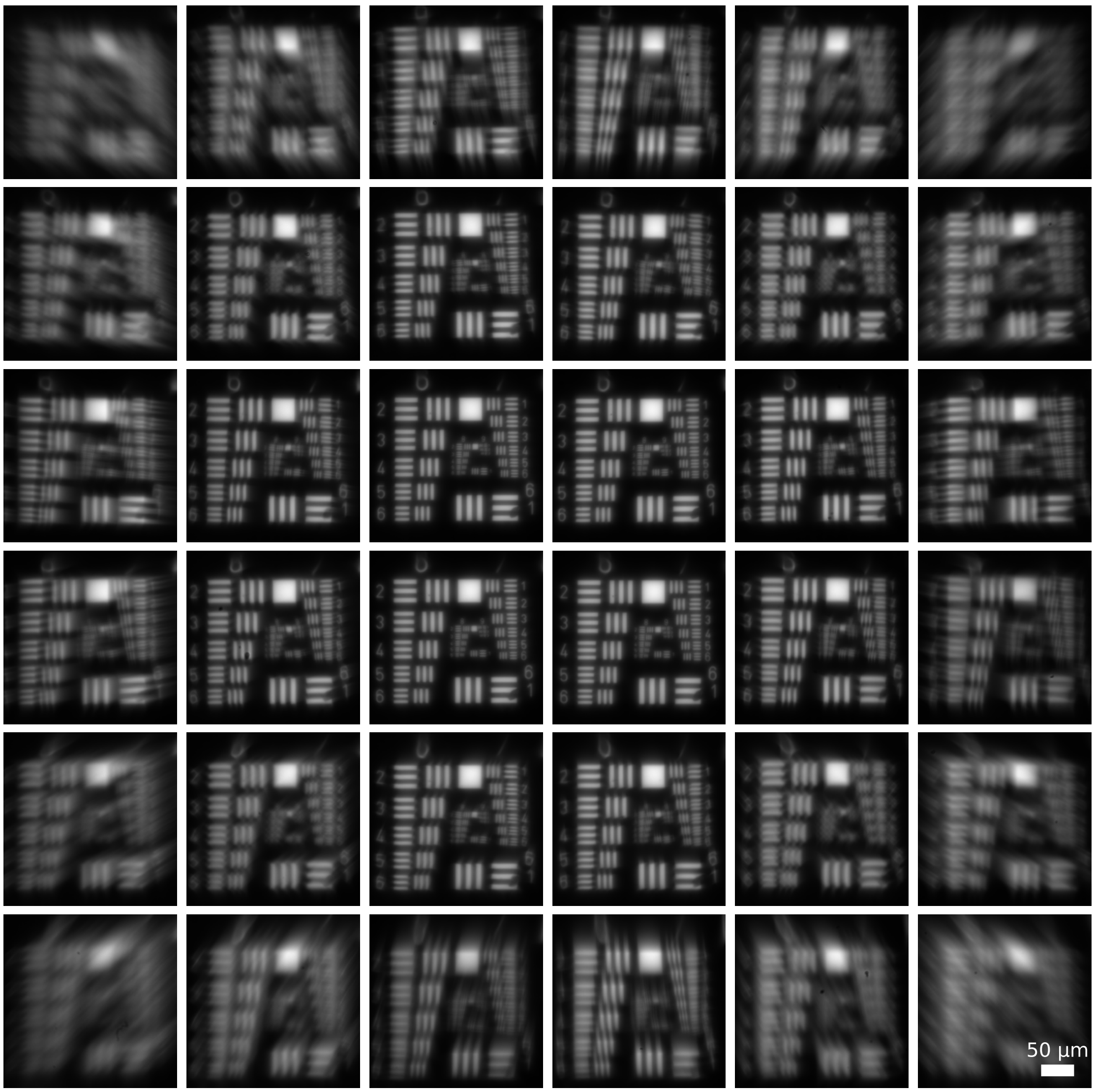

Fig. S14. Characterization of resolution across all 6 × 6 sensors in the fluorescence imaging configuration. Groups 6 and higher are shown. Zoom-ins of groups 8 and 9 are shown in Fig. S15.

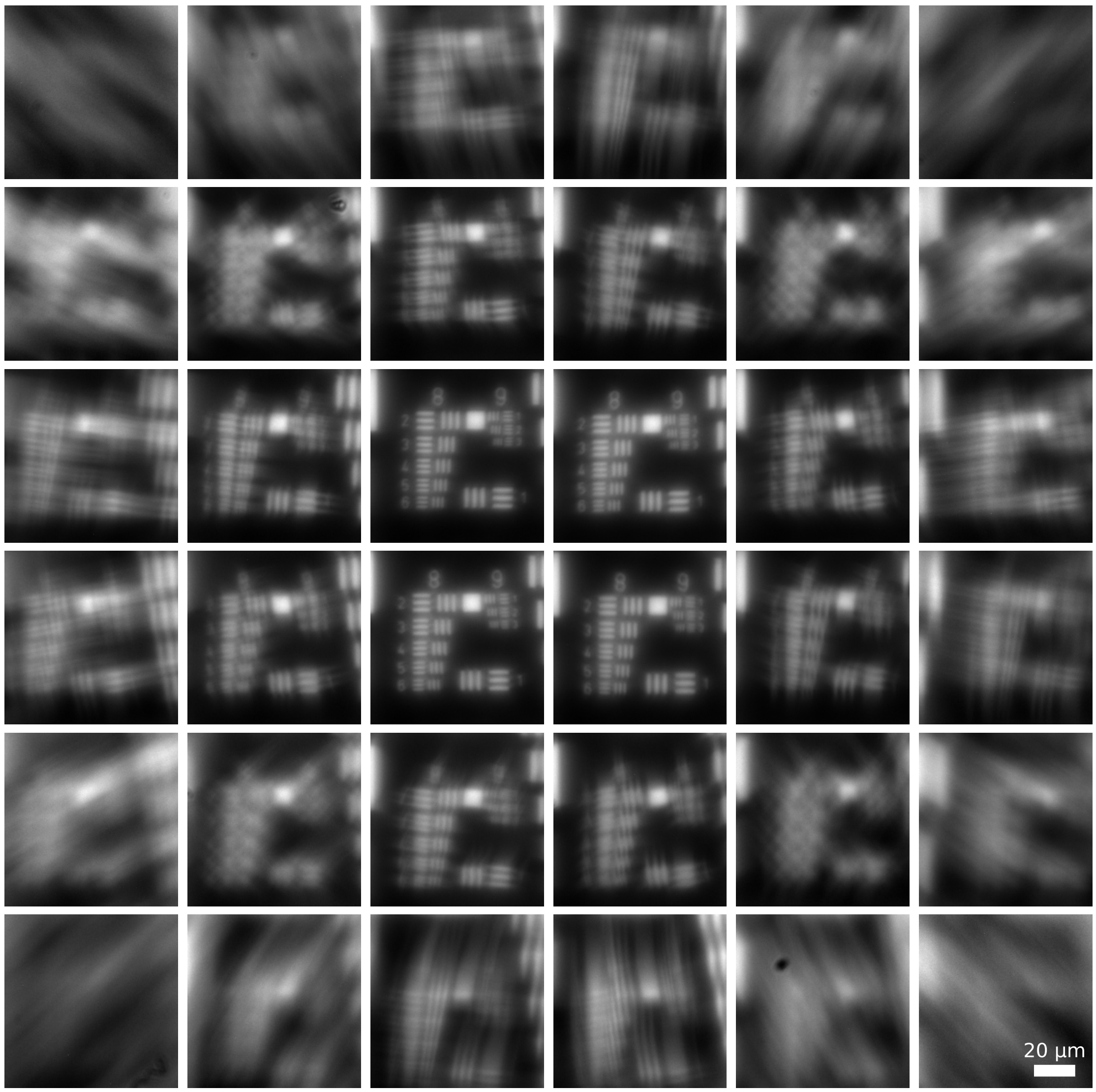

Fig. S15. Characterization of resolution across all 6×6 sensors in the fluorescence imaging configuration. Groups 8 and 9 are shown.

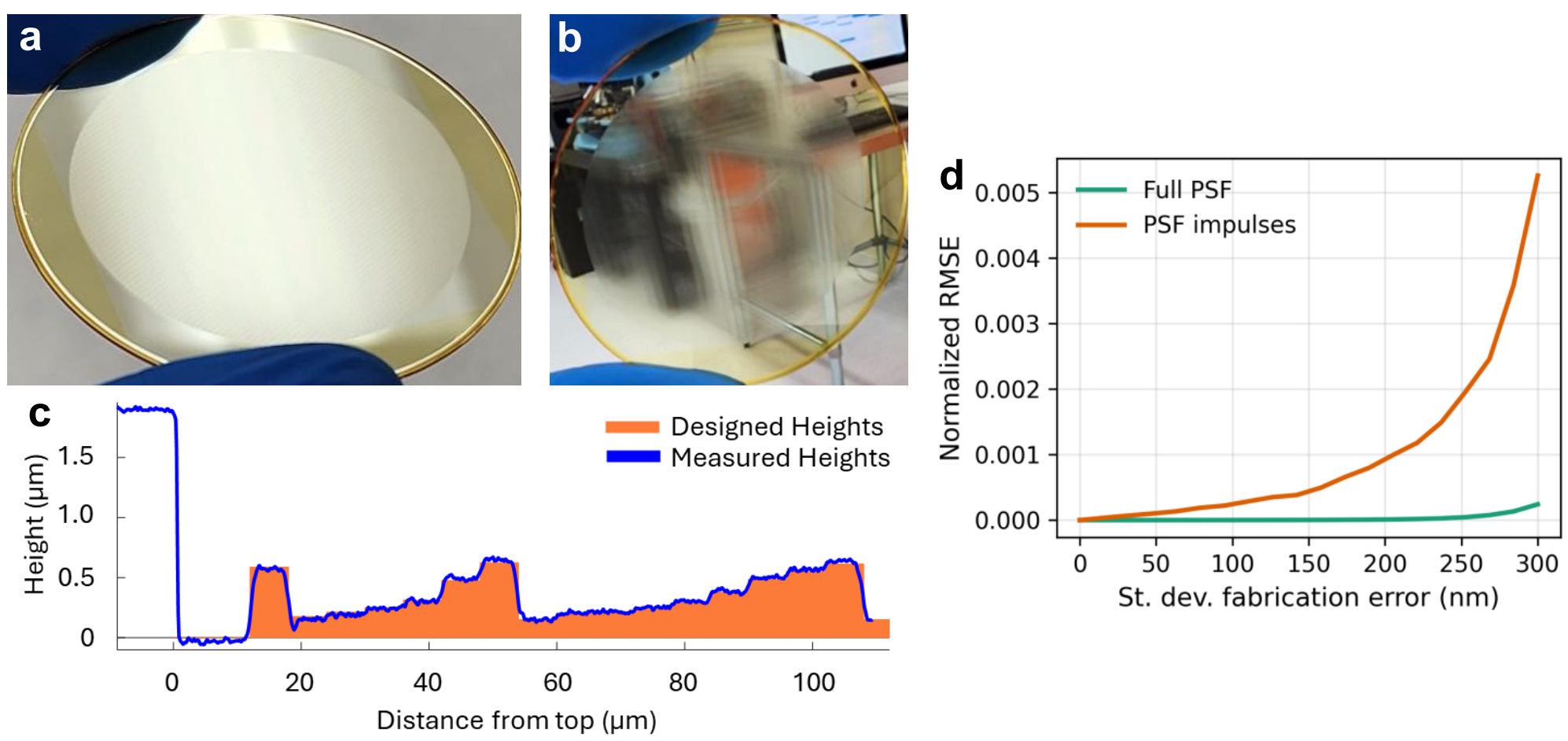

Fig. S16. (a-b) Photographs of the DOE used in our microscope. (b) illustrates the multiplexing effect of the DOE, superimposing multiple copies of the object/scene. (c) Characterization of the height profile of the DOE near edge of the pattern, using a confocal microscope (Olympus LEXT OLS5000), showing close agreement with the target design. (d) Sensitivity of PSF to DOE fabrication error (Gaussian noise with various standard deviations). We computed the root mean square error (RMSE) to the nominal PSF, normalized to the peak intensity of the nominal PSF. Two separate RMSE values were computed, one for the whole PSF, another for small window surrounding each of the 15 impulses. Each curve is an average of n=20 random noise realizations.

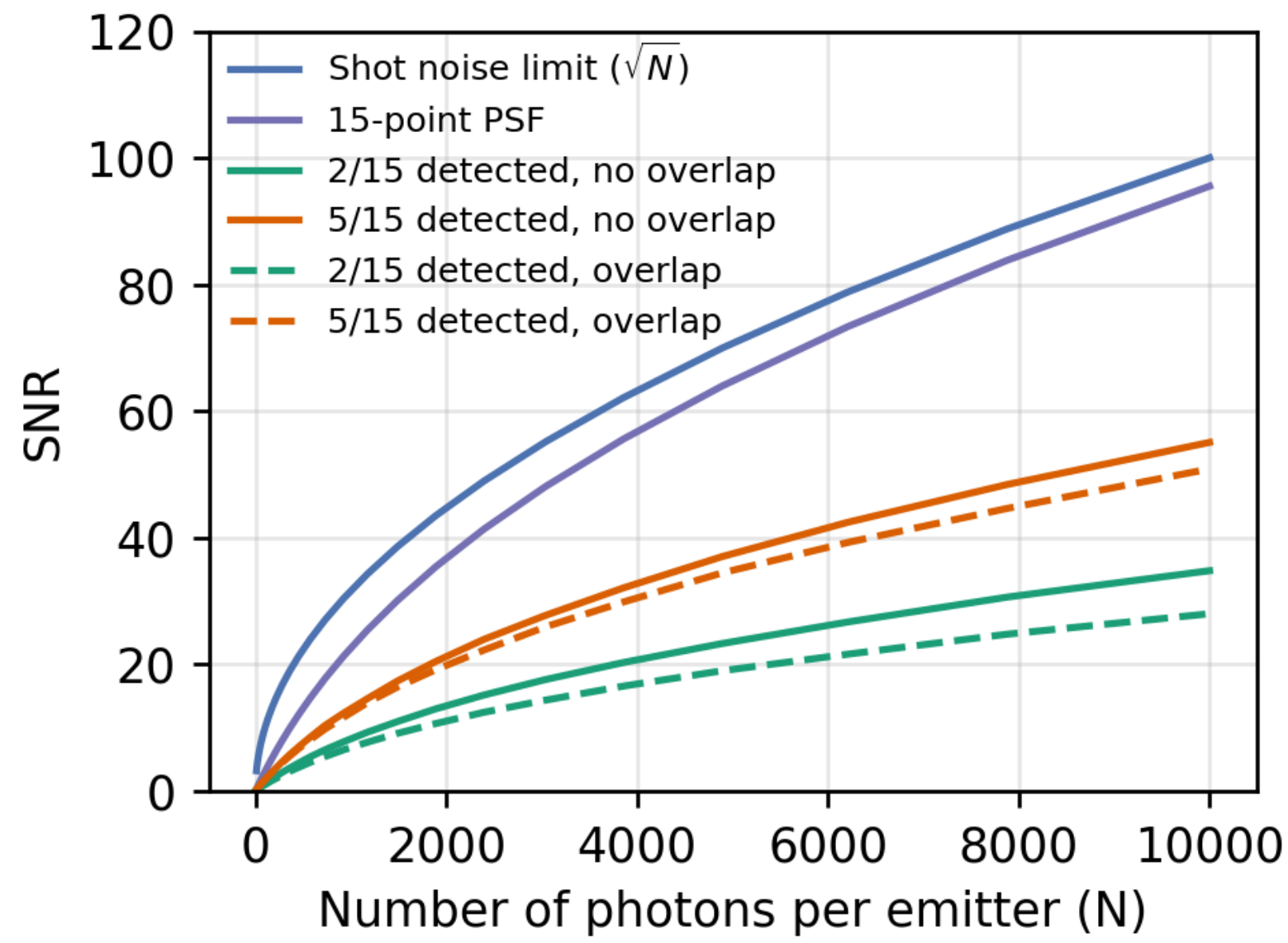

Fig. S17. SNR simulations under shot noise, read noise, and multiplexing (i.e., demixing overlapped signals). Two point emitters, N photons each, are imaged by our system, with the goal of estimating N using a least-squares solution (without regularization). The SNR is defined as the ratio of the mean and standard deviation of the estimates across n=50,000 random realizations. The blue curve represents the shot noise limit, while purple shows the effect of splitting the PSF into 15 points, incurring 15× more read noise (assuming $\sigma_{read} = 4e^-$ and each PSF impulse occupies 2×2 pixels). The green and orange curves illustrate the effect of splitting the PSF into 15 points, where, due to the gappy sensor array, only 2 or 5 of those 15 points are detected, respectively, resulting in a large loss in SNR. Finally, the dotted curves show the SNR reduction effect of multiplexing (i.e., 1 impulse from each PSF overlaps). By breaking the PSF symmetry, we have minimized the effect of multiplexing by ensuring only 1 impulse overlap is possible.

References

1. O. Cossairt and S. Nayar, "Spectral focal sweep: Extended depth of field from chromatic aberrations," in *2010 IEEE International Conference on Computational Photography (ICCP),* (IEEE, 2010), pp. 1–8.
