# Supplementary material for: Large-scale compressive microscopy via diffractive multiplexing across a sensor array
Source: Nat Photonics. 2026 Jul 28;20(10):1240–6. doi: 10.1038/s41566-026-01974-4 (PMC13626960; doi:10.1038/s41566-026-01974-4)
Supplement: Supplementary file 2 [file 41566_2026_1974_MOESM2_ESM.pdf]

nature portfolio

Peer Review File

Large-scale compressive microscopy via diffractive multiplexing across a sensor array

Corresponding Author: Professor Laura Waller

This file contains all reviewer reports in order by version, followed by all author rebuttals in order by version.

Version 0:

Reviewer comments:

Reviewer #1

(Remarks to the Author)
The manuscript presents an interesting integration of multi-sub-camera capture and PSF engineering to extend the field of view (FOV), leading to the development of mesoscale imaging systems for the study of multiple animals. The optical design is well described, and the corresponding results are clearly presented. The FOV enhancement primarily arises from: a) the use of multiple sensor arrays that fill the objective's effective area; b) a PSF design with distributed multi-spots that allow peripheral information to be captured within the sensor's coverage. Overall, the optical design is concise, and the shift-variant forward imaging model is formulated. Nevertheless, I have some concerns regarding the general applicability and interpretation of the method listed below:

1) The use of a multi-spot PSF introduces additional sparsity constraints on the observed scenes and may increase computational complexity. The manuscript thoughtfully acknowledges that the current PSF design is not "optimal by any metric," but further quantitative characterization of the PSF properties would strengthen the work and guide future improvements. For instance, beginning with a simplified 1D analysis could provide useful intuition for optimizing multi-spot PSFs and developing more elegant design strategies.

2) As most optical energy is distributed across multiple PSF spots, the resulting signal could become more sensitive to noise. Even though the manuscript shows that increased light throughput can compensate for background noise, this trade-off may limit the imaging duration or total photon budget. It would be valuable to experimentally evaluate how the system would perform under equal light throughput conditions compared to a conventional single-spot PSF reconstruction.

3) Regarding Section 2.3, two radial-shift-variant magnifications are applied. However, it seems that the 2D spatial shift Δr_i for each impulse PSF is assumed to be spatially invariant. Given that the PSF is modeled as a sum of delta functions, it might be important to verify whether the spacing among PSF spots changes with field position, as this could affect reconstruction accuracy.

4) The comparison between the global shift-variant (delta-simplified) model and the patch-based model in Fig. S4 is interesting. While memory efficiency differences are understandable due to PSF simplification, it may be premature to conclude that patch-based approaches inherently perform worse. A patch-based model using the full wave-optics PSF (without delta simplification) might outperform the global model if properly optimized. Providing additional adjustment or tuning for the patch-based reconstruction would improve the fairness of the comparison.

5) In Fig. S5, the meaning of "density" is not entirely clear. Including examples with varying sample densities, quantitatively and explicitly defining how "density" is computed would help readers interpret the results more easily.

6) In Eq. (7), the notation $M(r_p, \Delta r_i)$ should be $M(r, \Delta r_i)$?.

7) The concept may be more accurately described as "spatial multiplexing" rather than "diffractive multiplexing", since the multiplexing mainly occurs through spatial sampling within the inter-sensor gap areas rather than through diffractive encoding. Clarifying this distinction in the title and text may help readers better understand the novelty of the approach.

(Remarks on code availability)
The codes are straightforward.

Reviewer #2

(Remarks to the Author)
The authors have developed a compressive wide field high resolution microscope using a diffractive optical element to create a coded PSF. The demonstrate several hundred megapixels estimated per frame at 120 fps, resulting in greater than 20 gigapixels per second image acquisition.

This is an obviously interesting instrument and publication in Nature Photonics is warranted. The authors use standard convex optimization techniques using total variation and l1 regularization to estimate the scene.

The manuscript would be substantially improved if the authors presented plots of the actual psf at various field points and analyzed the corresponding MTF. The MTF is presumably quite poor for such an extended PSF, so it would be useful to discuss the level of sparsity needed to recover the scene weak MTF and the how design might be optimized to improve MTF. The objects reconstructed are quite sparse, which means that the pixels per second specification is not really fair. The manuscript would be improved by considering how sparse the scene under test needs to be to recover an image.

The underlying assumptions of the Candes/Tao/Donoho compressive sensing theory are rarely satisfied in imaging systems, although they may be satisfied for images sparse on the natural basis. On the other hand, coding with a 15 lobe PSF in a shift invariant system definitely does not meet the random projection assumptions of CS theory. In view of this and many other performance issues, image reconstruction with TV/L1 optimization as implemented here is definitely not state of the art.
The paper would be greatly improved by using more appropriate estimation algorithms.

(Remarks on code availability)

Reviewer #3

(Remarks to the Author)
The manuscript by Zhou et al., titled "Large-scale compressive microscopy via diffractive multiplexing across a sensor array," proposes a snapshot computational microscope that uses a 6×8 sensor array and a pupil-plane DOE to generate a non-symmetric 15-spot PSF. With 4×4 binning to reach 120 fps acquisition, the system reports darkfield reconstructions at ~2.76 µm resolution over 5.3 cm² and fluorescence at ~4.38 µm resolution over ~9.3×9.3 mm².
The engineering is excellent, and the advance is interesting. There are a few points that merit clarification.
- The paper emphasizes a ~210 MP/frame reconstructed output and ~25.2 GP/s equivalent throughput; however, these reflect the reconstruction grid rather than the raw sampling.
- Reconstruction uses a fully shift-variant forward model with TV+L1 regularization and non-negativity, implemented via direct superposition;
- The DOE (64-level grayscale lithography) and PSF layout are chosen using a triple-correlation criterion. Demonstrations include static targets and C. elegans videos in darkfield and GCaMP fluorescence; fluorescence performance is good, yet non-uniform across the field, and the method currently operates offline rather than end-to-end in real time.
These points were not clear in the initial reading of the abstract, and it could be good to clarify or be more precise about the claims.

Here are some questions and concerns:

1. The C. elegans data is quite sparse, and this can aid in reconstruction. Can the authors demonstrate performance in dense samples and compare with ground truth data with more conventional imaging?
2. Generally, can the authors show when performance breaks down? What are the limits?
3. The paper did not explicitly report the raw pixel count per frame after 4x4 binning. It should be around 38.5 MP. The authors should differentiate the physical sampling throughput and reconstructed output pixels. They should be clear about the claims so that readers do not get confused about the performance.
4. The PSF design of the DOE element is done through computer simulation and triple correlation. It would be nice if the author could provide more information on this.
a. More simulation results on DOE fabrication
b. Monte Carlo tolerance analysis
c. Maybe detail the metrology process
5. The authors could include the synchronization process of all the imaging sensors (global or rolling shutter). Would the assembly tip/tilt affect image reconstruction?
6. The authors claim that the FOV could be expanded to 6-7 mm. The author could quantify the extrapolation boundary by showing a map with number of PSF at each location. The quality of the PSF would degrade at the edge, which might not be suitable for imaging.
7. 8-bit readout is a low dynamic range. The signal might saturate when having a high fluorescence signals and/or weak

signals can get lost in the noise. The authors could provide more information on the characteristics of intensity histograms. Showing cases where the quality of the reconstructed image is poor could help the reader understand the limits.

(Remarks on code availability)

Version 1:

Reviewer comments:

Reviewer #1

(Remarks to the Author)
The authors have conducted many experiments to address my previous concerns, which have now been fully addressed. I would recommend its publication in the current form.

(Remarks on code availability)
The codes are fine.

Reviewer #2

(Remarks to the Author)
The authors have responded extensively to reviewer comments and addressed them well in the manuscript. I do not believe that further revision is needed, the manuscript can be published now.

(Remarks on code availability)

Reviewer #3

(Remarks to the Author)
I appreciate the authors' response. I have no further items. It is an interesting advance and merits sharing.

(Remarks on code availability)

Open Access This Peer Review File is licensed under a Creative Commons Attribution 4.0 International License, which permits use, sharing, adaptation, distribution and reproduction in any medium or format, as long as you give appropriate credit to the original author(s) and the source, provide a link to the Creative Commons license, and indicate if changes were made.
In cases where reviewers are anonymous, credit should be given to 'Anonymous Referee' and the source.

The images or other third party material in this Peer Review File are included in the article's Creative Commons license, unless indicated otherwise in a credit line to the material. If material is not included in the article's Creative Commons license and your intended use is not permitted by statutory regulation or exceeds the permitted use, you will need to obtain permission directly from the copyright holder.
To view a copy of this license, visit https://creativecommons.org/licenses/by/4.0/

Reviewer #1 (Remarks to the Author):

The manuscript presents an interesting integration of multi-sub-camera capture and PSF engineering to extend the field of view (FOV), leading to the development of mesoscale imaging systems for the study of multiple animals. The optical design is well described, and the corresponding results are clearly presented. The FOV enhancement primarily arises from: a) the use of multiple sensor arrays that fill the objective's effective area; b) a PSF design with distributed multi-spots that allow peripheral information to be captured within the sensor's coverage. Overall, the optical design is concise, and the shift-variant forward imaging model is formulated.
We thank the Reviewer for their positive assessment!

Nevertheless, I have some concerns regarding the general applicability and interpretation of the method listed below:
R1.1. The use of a multi-spot PSF introduces additional sparsity constraints on the observed scenes and may increase computational complexity. The manuscript thoughtfully acknowledges that the current PSF design is not "optimal by any metric," but further quantitative characterization of the PSF properties would strengthen the work and guide future improvements. For instance, beginning with a simplified 1D analysis could provide useful intuition for optimizing multi-spot PSFs and developing more elegant design strategies.

Although we acknowledge that the current PSF design is not optimal by any metric, we'd like to clarify that we still designed the PSF and optimized its properties based on several quantitative guiding principles/constraints (as detailed in Supplementary Sec S1).

The most fundamental one is achieving a continuous, gap-free reconstruction, which requires the PSF to be spatially extended – this is a hard constraint that requires the PSF to be at least as large as the largest gap in the sensor array. Another fundamental constraint is that the PSF should have minimal translational ambiguity, meaning that no two field positions should give rise to the same measurement, and measurements from any two positions should be as dissimilar as possible. Conventional imaging systems quantify this property by looking at the autocorrelation of the PSF, or the modulation transfer function (MTF). Even if we ignore aberrations and distortions, and assume a perfect 4F system, the gaps in the sensor array make our system not linear shift-invariant. Hence, traditional MTF/autocorrelation-based criteria are not directly applicable. For example, a PSF could be "good" for one part of the FOV where most of it lands on an active sensor, yet "bad" at another part of the FOV where it lands on a gap. We account for this shift variance by generalizing the PSF autocorrelation to a triple correlation (i.e., between the PSF, the PSF (again), and the sensor array), which is a more computationally efficient shortcut for assessing the rank of the linear forward model describing the imaging system. Future PSF designs should still follow these fundamental constraints/criteria.

Another constraint is that the PSF should be sparse, since a distributed PSF can suffer from low SNR in low-light imaging conditions. In principle this soft constraint still gives us a lot of freedom in the PSF design, but in practice we limited ourselves to a discrete number of impulses, a design choice well-supported by the broader computational imaging literature. For instance,

recent work on lensless imagers by Kabuli et al. (*"Designing lensless imaging systems to maximize information capture," Optica, 13(2), 227–235, 2026*) similarly constrained their search to lenslet-based designs (resulting in a PSF consisting of a set of impulses) for the same reason. The discrete impulse design also helped with computational complexity of the full-scale inverse problem when we reconstructed images using a real-space superposition method. Nevertheless, this design heuristic is not required, and future designs can impose softer spatial sparsity constraints.

Since we have decided on a multi-impulse PSF parameterization and a pupil-plane DOE implementation, another soft constraint is that the PSF should be as small in extent as possible to minimize chromatic aberrations by the DOE. In other words, the further away an impulse is from the zero-order impulse, the more radially blurred that impulse becomes (in proportion to the radial distance). As such, we constrained the PSF to be as small as possible, while still satisfying the hard constraint of gap-free reconstruction. Again, this constraint is not strictly required, and future PSF design optimizations can jointly consider the DOE chromatic aberrations with the PSF sparsity.

Even within these constraints, there are still an infinite number of possible PSFs and so future designs can add further constraints or design principles in addition to relaxing some of the ones we chose, depending on the type of samples and applications. For example, although our current design was driven by spatial sparsity, samples that are sparse in other domains (e.g., wavelet, gradient, Fourier) would warrant other PSF designs, perhaps data-driven.

We have added a new paragraph in the Discussion section that reflects this above discussion (also in response to R2.2 below), complementing the existing discussion in Supplementary Sec. S1:

> "The PSF design for our compressive microscope also warrants further discussion. Here, we used a design heuristic based on a discrete distribution of impulses that maximizes the rank of the linear forward model, while balancing SNR considerations (Supplementary Sec. S1; Fig. S17). Our heuristic also reduces the column correlations of our linear model by breaking PSF symmetry so that the maximum PSF overlap between any two field positions is a single impulse. However, there are infinitely many PSFs that satisfy these criteria. Furthermore, such a design presupposes sparsity in the native space domain, which we enforced by ensuring sparse distributions of *C. elegans*. Thus, there is room for future investigation to further optimize the PSF design for more general samples based on sparsity in other domains or in a data-driven fashion. Such multiplexing PSF designs must be balanced with practical constraints, such as low photon counts typical of high-speed fluorescence imaging, nonnegativity of the PSF, and chromatic aberrations of larger PSFs."

R1.2. As most optical energy is distributed across multiple PSF spots, the resulting signal could become more sensitive to noise. Even though the manuscript shows that increased light throughput can compensate for background noise, this trade-off may limit the imaging duration or total photon budget. It would be valuable to experimentally evaluate how the system would

perform under equal light throughput conditions compared to a conventional single-spot PSF reconstruction.

Indeed, there is an important tradeoff between multiplexing and SNR – a problem common to any compressive/multiplexing approach (Kabuli et al. *Optica*, 13(2), 2026). The Reviewer is correct that distributing light across multiple pixels makes our imaging system more susceptible to noise for at least two reasons: 1) if there is significant sensor read noise, splitting the light into k times more pixels results in k times more read noise events, and 2) computationally demixing signals overlapped from multiple object points also incurs an SNR penalty (due to non-zero correlations in the columns of the A matrix in our linear forward model, y=Ax). We can simulate the effects of both contributions by examining the task of reconstructing two point emitters under shot noise, read noise, and multiplexing (overlapping PSFs). The analysis is complicated by the fact that SNR will vary for different parts of the FOV, depending on how many of the impulses making up the PSF land on an active sensor area. For example, a single-spot PSF will have shot noise-limited SNR for areas of the image where sensors are placed, but zero SNR in the gap areas.

To this end, we have created a new figure (Fig. S17), which shows the SNR vs. number of emitted photons under different scenarios:

- The shot noise limit (blue), which is the case of a diffraction-limited single-spot PSF that lands on an active sensor area (i.e. a gap-free sensor)
- Splitting the PSF into 15 points across a gap-free sensor (purple), illustrating the effect of 15x more read noise without accounting for sensor gaps
- Splitting the PSF into 15 points, of which only 2 (green) or 5 (orange) are detected, representing typical scenarios in our setup.
- Finally, the same scenario as the previous bullet, except the two PSFs happen to have one of the impulses from each overlapping with one of the impulses from the other, causing further ambiguity (dotted lines).

As one might expect, the biggest effect on SNR comes from light lost in the sensor gaps. We have minimized the effect of multiplexing by breaking the PSF symmetry, resulting in a worst-case scenario of overlap at a single PSF impulse. As the Reviewer points out, our multiplexing strategy may thus limit applications like fluorescence imaging for which fluorophores emit only a finite number of photons before they photobleach.

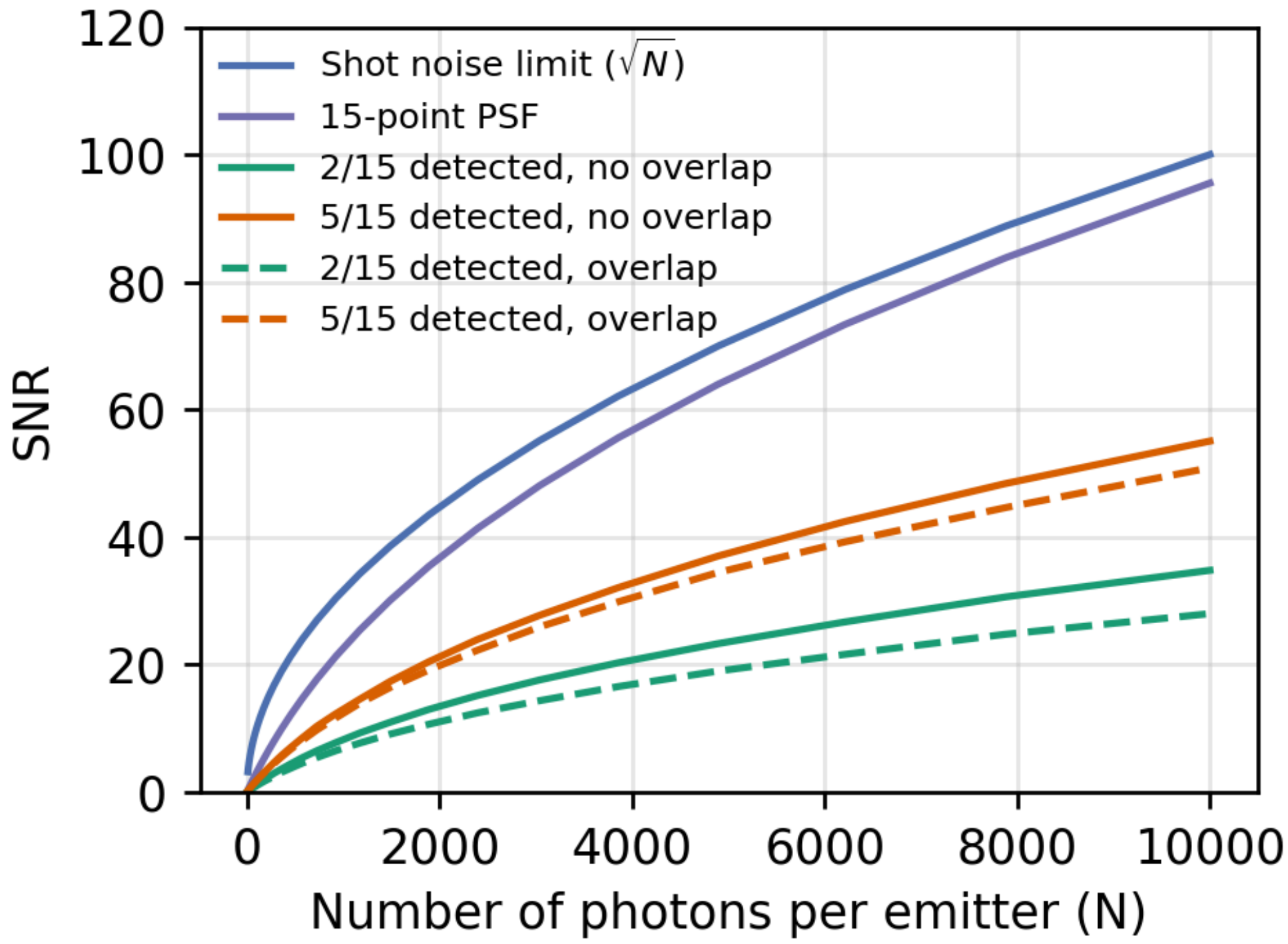

Fig. S17. SNR simulations under shot noise, read noise, and multiplexing (i.e., demixing overlapped signals). Two point emitters, N photons each, are imaged by our system, with the goal of estimating N using a least-squares solution (without regularization). The SNR is defined as the ratio of the mean and standard deviation of the estimates across n=50,000 random realizations. The blue curve represents the shot noise limit, while purple shows the effect of splitting the PSF into 15 points, incurring 15× more read noise (assuming σ_{read} = 4e− and each PSF impulse occupies 2×2 pixels). The green and orange curves illustrate the effect of splitting the PSF into 15 points, where, due to the gappy sensor array, only 2 or 5 of those 15 points are detected, respectively, resulting in a large loss in SNR. Finally, the dotted curves show the SNR reduction effect of multiplexing (i.e., 1 impulse from each PSF overlaps). By breaking the PSF symmetry, we have minimized the effect of multiplexing by ensuring only 1 impulse overlap is possible.

This new figure is now referenced in the Discussion section in the third paragraph.

R1.3. Regarding Section 2.3, two radial-shift-variant magnifications are applied. However, it seems that the 2D spatial shift Δr_i for each impulse PSF is assumed to be spatially invariant. Given that the PSF is modeled as a sum of delta functions, it might be important to verify whether the spacing among PSF spots changes with field position, as this could affect reconstruction accuracy.

We thank the Reviewer for bringing this up, as it is important to clarify that our imaging system is modeled with a shift-variant PSF. Eq. 3 gives a simplified shift-invariant model for clarity, while Eqs. 5-6 show the two separable radial distortion models that capture the shift-variance. To experimentally show that the PSF does indeed change with field position, we measured the PSF

at two different field positions and compared a shift-invariant model with our shift-variant model (Eqs. 5-6):

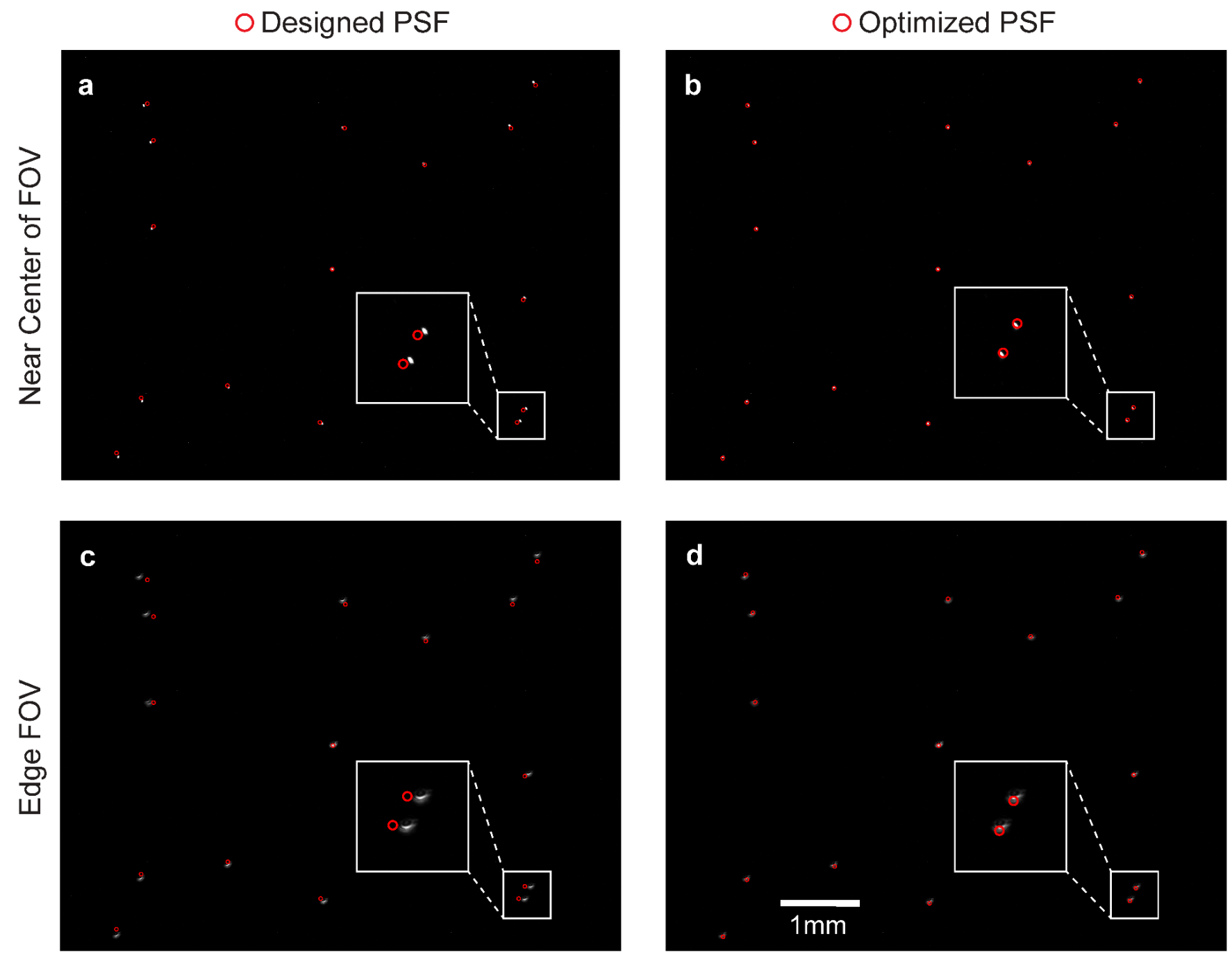

Fig. S9. Experimental validation of the computational distortion model. Stitched experimental PSFs are overlaid with (a, c) the initially designed coordinates and (b, d) the computationally parameterized coordinates after co-optimization (red symbols). The top row (a, b) displays the PSF measured near the center of the field-of-view (FOV), and the bottom row (c, d) displays the PSF at the edge of the FOV. PSFs are intentionally saturated to aid in visualization.

As demonstrated, these optimized PSFs result in an improved alignment at both locations, verifying that our parameterized distortion model accurately captures the physical distortions of the system.

R1.4. The comparison between the global shift-variant (delta-simplified) model and the patch-based model in Fig. S4 is interesting. While memory efficiency differences are understandable due to PSF simplification, it may be premature to conclude that patch-based approaches inherently perform worse. A patch-based model using the full wave-optics PSF (without delta simplification) might outperform the global model if properly optimized. Providing additional adjustment or tuning for the patch-based reconstruction would improve the fairness of the comparison.

This is a good point, and we agree that patch-based models are not inherently inferior; we have modified the text to reflect this (at the end of Sec 2.2):

> "While our computationally efficient, spatially varying, direct convolution approach enabled fast reconstructions, a patch-based FFT approach could yield more accurate results by modeling the aberrations of the individual PSF impulses."

A properly optimized patch-based model using a full wave-optics PSF, rather than assuming delta impulses, is indeed theoretically highly capable, which could potentially improve resolution by deconvolving the aberrations. However, as demonstrated in Supplementary Section S3 and Fig. S4, patch-based reconstruction underperforms our direct method when constrained by a similar computational budget. Uniform patching inherently assumes that the PSF varies slowly across the FOV and remains approximately shift-invariant within each patch. While this assumption holds near the center of the FOV, it breaks down significantly in the periphery, where the PSF exhibits much more rapid spatial variations. To overcome this limitation and match or exceed the peripheral accuracy of our delta-simplified, fully shift-variant model, the patch-based method would require much finer patch divisions at the edges of the FOV. This, in turn, would incur a prohibitive memory cost, rendering it unsuitable for our goal of full-resolution reconstruction without downsampling.

R1.5. In Fig. S5, the meaning of "density" is not entirely clear. Including examples with varying sample densities, quantitatively and explicitly defining how "density" is computed would help readers interpret the results more easily.
We thank the Reviewer for the constructive feedback. We have updated Supplementary Section S5 to explicitly define density as the fraction of non-zero pixels across the object field-of-view:

> "Here, density is defined as the percentage of non-zero points within the reconstructed FOV."

We agree with the Reviewer that including examples with varying densities would be helpful – to that end, have also generated new experimental results using a smartphone display as the sample to enable direct control over the sample density. Our new Supplementary Fig. S6 shows these new results:

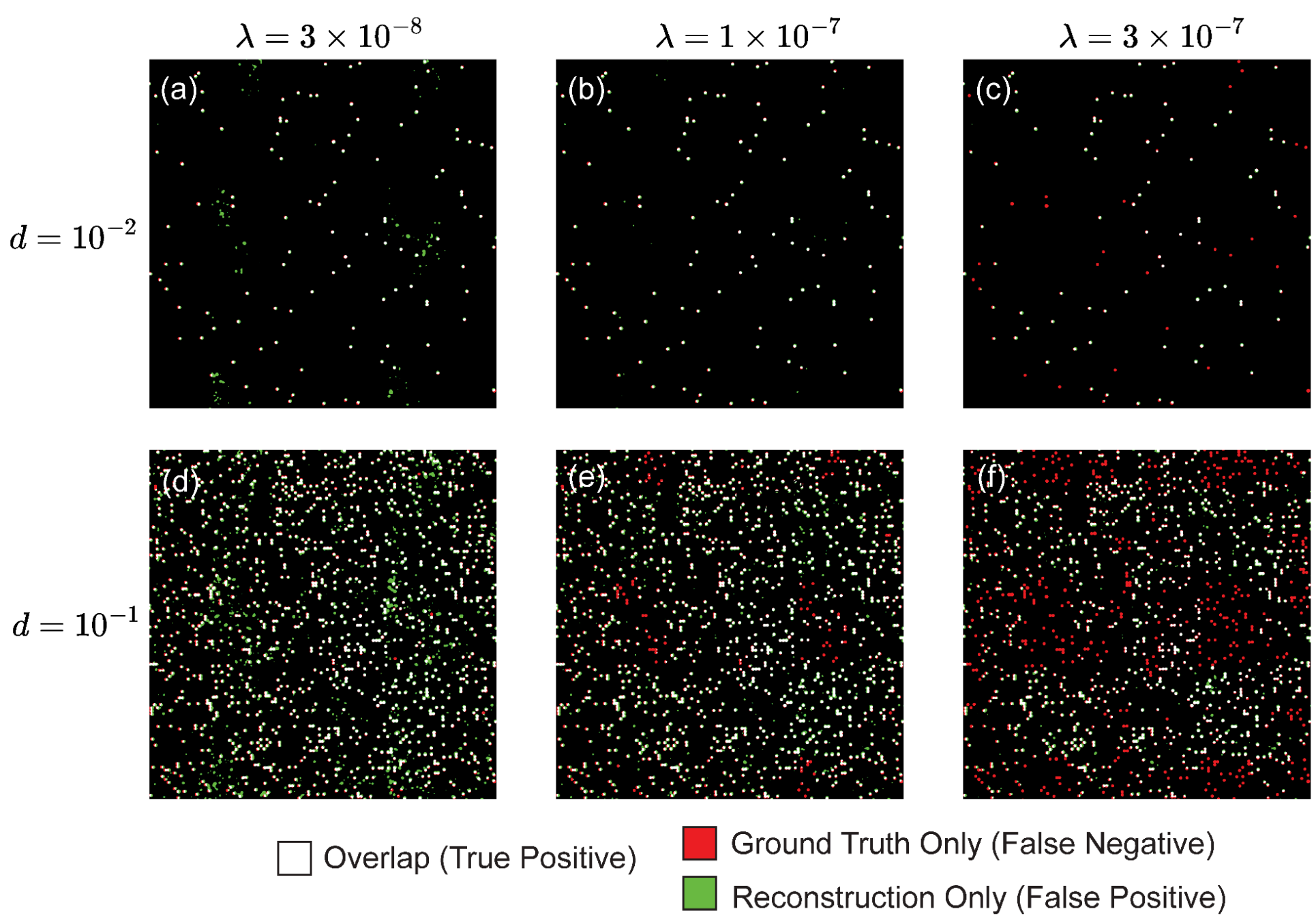

Fig. S6. Experimental evaluation of reconstruction fidelity versus object density. Binarized overlays (threshold 0.1) comparing reconstructions across varying L_1

regularization strengths (λ). White indicates accurate recovery (true positive), red indicates missed ground truth (false negative), and green indicates artifacts (false positive). (a-c) Reconstructions at a lower object density ($d=10^{-2}$), showing near-perfect overlap at the optimal λ. (d-f) Reconstructions at a higher object density ($d=10^{-1}$), exhibiting increased false positives and false negatives across all evaluated λ values. All panels display a representative ~6.3 x 6.3 mm^2 physical region cropped from the center of the full multi-centimeter field-of-view.

R1.6. In Eq. (7), the notation $M(r_p, \Delta r_i)$ should be $M(r, \Delta r_i)$?.
Yes, thank you for catching this error. We originally used r_p to denote a point in the object plane at position r_p, but in Eq. (7) this is equivalent to r. We have updated the notation to $M(r, \Delta r_i)$.

R1.7. The concept may be more accurately described as "spatial multiplexing" rather than "diffractive multiplexing", since the multiplexing mainly occurs through spatial sampling within the inter-sensor gap areas rather than through diffractive encoding. Clarifying this distinction in the title and text may help readers better understand the novelty of the approach.
Indeed, "spatial multiplexing" accurately characterizes our approach; however, "multiplexing" can take on different meanings in compressive sensing compared to conventional imaging – in the latter case, we often see "multiplexing" take on a similar meaning to "parallelizing", such that "spatial multiplexing" could be interpreted as simply using many spatially offset camera sensors (which we also do in our method). Thus, to emphasize that the diffractive element and sensor array are working in concert, we believe that "diffractive multiplexing" conveys our message slightly more clearly.

Reviewer #1 (Remarks on code availability):

The codes are straightforward.

Reviewer #2 (Remarks to the Author):

The authors have developed a compressive wide field high resolution microscope using a diffractive optical element to create a coded PSF. The demonstrate several hundred megapixels estimated per frame at 120 fps, resulting in greater than 20 gigapixels per second image acquisition.

This is an obviously interesting instrument and publication in Nature Photonics is warranted. The authors use standard convex optimization techniques using total variation and l1 regularization to estimate the scene.
We thank the Reviewer for their positive assessment!

R2.1. The manuscript would be substantially improved if the authors presented plots of the actual psf at various field points and analyzed the corresponding MTF. The MTF is presumably quite poor for such an extended PSF, so it would be useful to discuss the level of sparsity needed to recover the scene weak MTF and the how design might be optimized to improve MTF. The objects reconstructed are quite sparse, which means that the pixels per second specification is not really fair. The manuscript would be improved by considering how sparse the scene under test needs to be to recover an image.
We thank the Reviewer for these constructive suggestions. We have added significantly to the manuscript to address these concerns, which are indeed important. We note that the extent of the PSF can be quite large without significantly degrading the MTF, if each PSF point is a diffraction-limited spot; the primary downside is degraded SNR, as seen above in R1.2.

First, we included visualizations of the actual stitched PSFs at multiple field points in Supplementary Fig. S9:

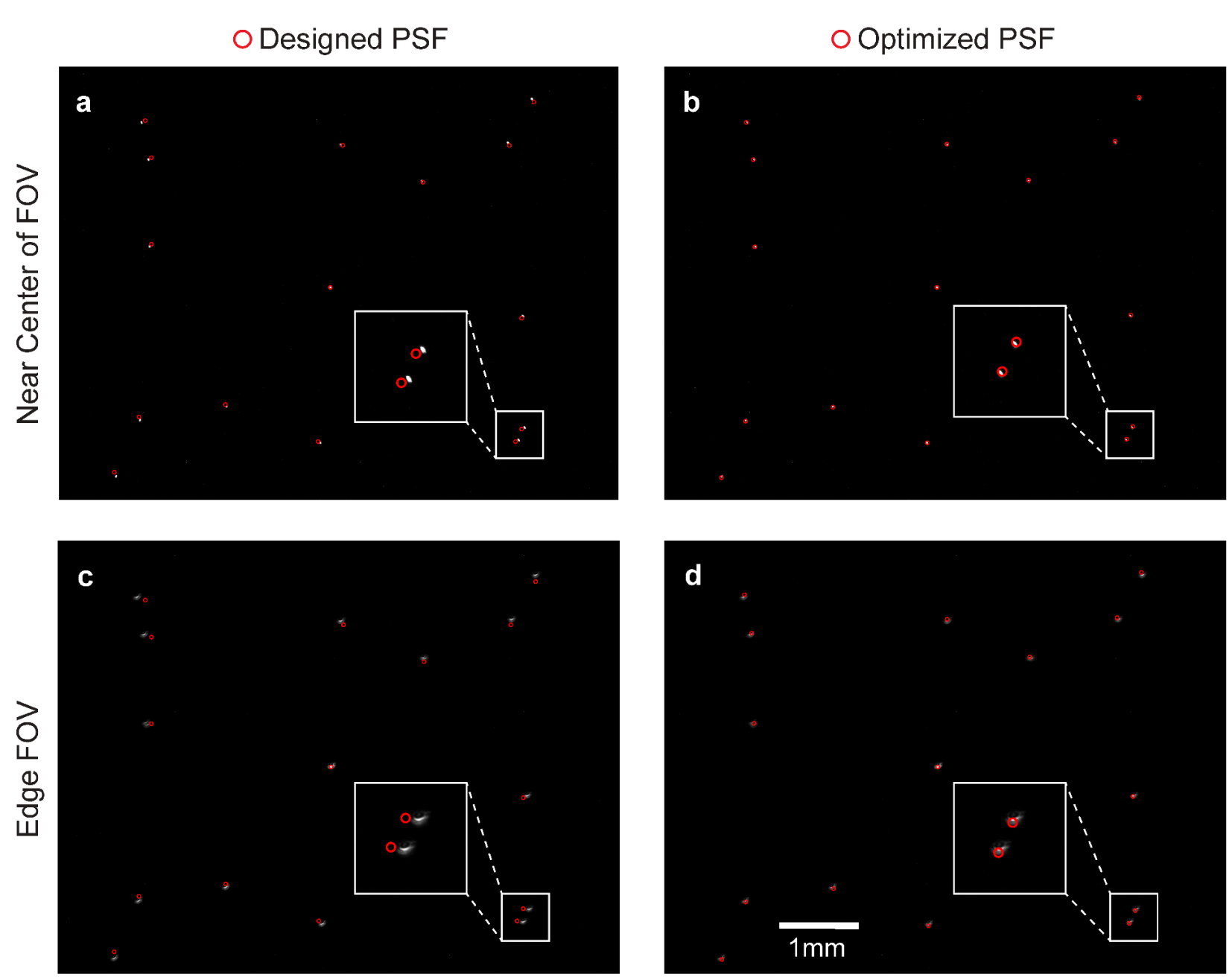

Fig. S9. Experimental validation of the computational distortion model. Stitched experimental PSFs are overlaid with (a, c) the initially designed coordinates and (b, d) the computationally parameterized coordinates after co-optimization (red symbols). The top row (a, b) displays the PSF measured near the center of the field-of-view (FOV), and the bottom row (c, d) displays the PSF at the edge of the FOV. PSFs are intentionally saturated to aid in visualization.

While the MTF magnitude for our extended, multi-spot PSF is indeed lower than that of an ideal single-point PSF, the extended nature of the PSF is one of our system's core requirements for filling in the gaps between sensors. In particular, our PSF must be physically extended to span the largest inter-sensor gaps. Supplementary Figs. S12-S15 give a sense of the MTF at various field positions across the extended FOV, specifically the contribution due to the lens aberrations. We note that the impulses of our PSF are not diffraction-limited, due to lack of lens options with high space-bandwidth products (>100 MP). As such, our fluorescence imaging mode throughput is aberration-limited, but our darkfield imaging mode is approximately sampling-limited despite not being diffraction-limited.

To explicitly address how sparse the scene needs to be for successful recovery, we have added new experimental results for imaging objects with varying sparsity levels, along with a quantitative analysis (Supplementary Section S6 and Figures S6-S7). Empirically, we establish a threshold for artifact-free recovery at target densities of ~10^{-2} or lower.

- Sec. S6 (Impact of sample sparsity on reconstruction quality):

  To quantify the effect of sample sparsity on reconstruction fidelity, we conducted an empirical study using a programmable target. Sparsity is quantified by the sample density, d, defined as the fraction of non-zero pixels across the object field-of-view (FOV). We utilized the darkfield imaging configuration, which consists of an FJW Industries objective (f = 90 mm), a 514/2 nm narrowband filter, and a 40-mm-diameter aperture (though note that we did not perform darkfield illumination). To provide a controllable ground truth (GT) across the full FOV, we employed an iPhone 17 Pro screen as the imaging target, generating synthetic patterns consisting of randomly scattered green dots (corresponding to ~20 μm OLED pixels).

  The qualitative results, illustrated in Fig. S6, reveal an empirical threshold for successful image recovery. At a high density ($d > 10^{-2}$), the system is unable to achieve a high-fidelity reconstruction. Regardless of the ℓ_1 regularization weight (λ) applied, the algorithm either fails to reconstruct certain points (false negatives, appearing as red in the overlay) or introduces artifacts (false positives, appearing as green). Conversely, when the sample is sufficiently sparse ($d \sim 10^{-2}$ or lower), artifact-free recovery is readily achieved using an optimal sparsity regularization weight. In these highly sparse regimes, the reconstruction and ground truth are nearly perfectly coincident, appearing as white.

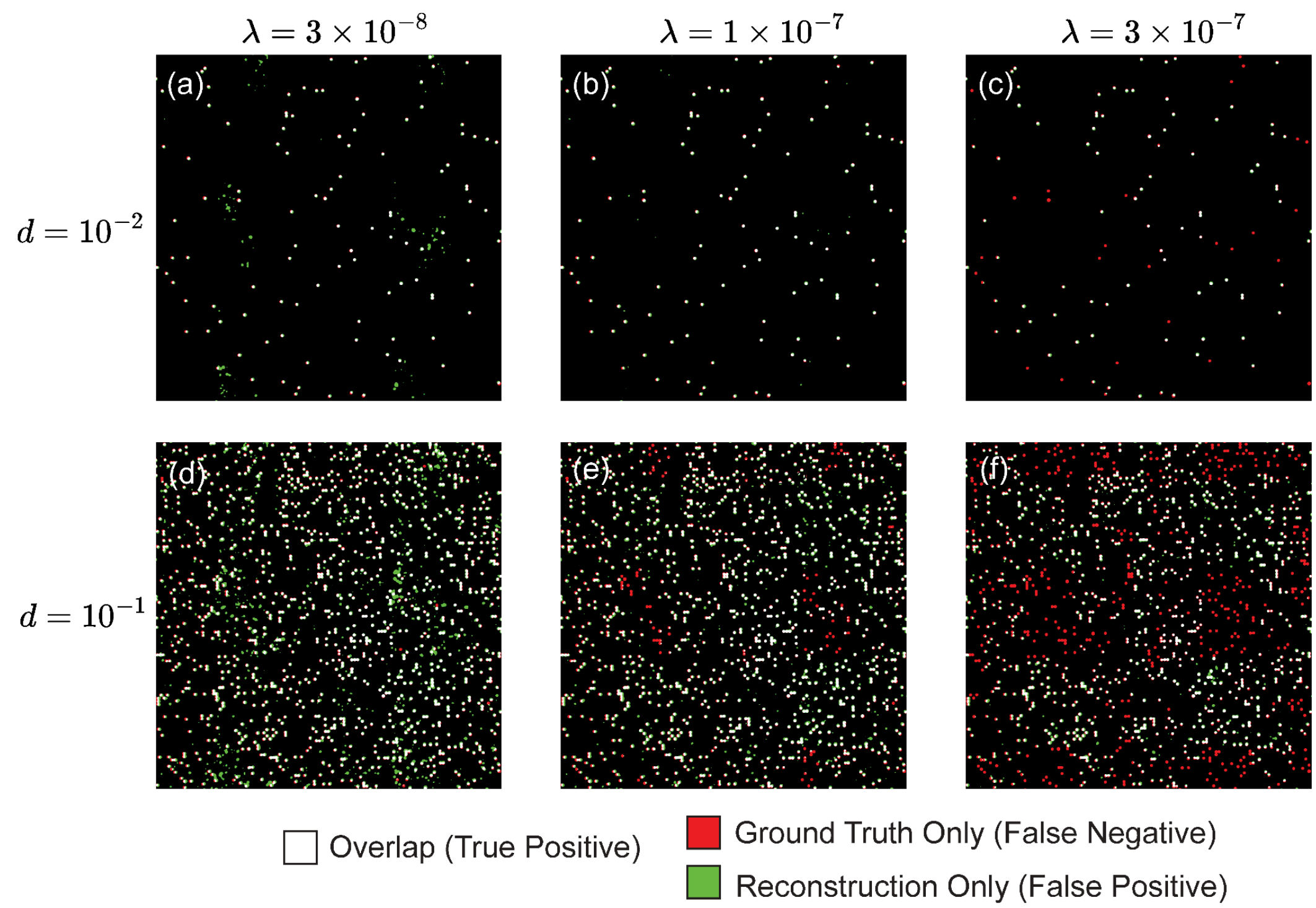

Fig. S6. Experimental evaluation of reconstruction fidelity versus object density. Binarized overlays (threshold 0.1) comparing reconstructions across varying L_1 regularization strengths (λ). White indicates accurate recovery (true positive), red indicates missed ground truth (false negative), and green indicates artifacts (false positive). (a-c) Reconstructions at a lower object density ($d=10^{-2}$), showing near-perfect overlap at the optimal λ. (d-f) Reconstructions at a higher object density ($d=10^{-1}$), exhibiting increased false positives and false negatives across all evaluated λ values. All panels display a representative ~6.3 x 6.3 mm^2 physical region cropped from the center of the full multi-centimeter field-of-view.

To benchmark our reconstruction performance across varying target densities, we quantitatively evaluated both the continuous pixel intensities and the binary recovery of the dots displayed on the screen in Fig. S7. Prior to computing performance metrics, both the reconstructed images and the ground truth were downsampled by 8× for computational efficiency and smoothed with a Gaussian kernel (σ = 10 pixels). This blurring step accommodates small residual distortion, ensuring the evaluation focuses on successful target recovery. First, we measured the mean squared error (MSE) between these preprocessed images to quantify overall intensity accuracy. Second, we treated the dot recovery as a pixel-wise classification problem. By applying varying intensity thresholds, we generated receiver operating characteristic (ROC) curves and calculated the area under the curve (AUC) for each density level. An AUC of 1.0 indicates a perfect reconstruction, while 0.5 represents a random guess. As demonstrated across multiple regularization weights (λ), reconstruction fidelity remains high for target densities below 10^{-2}. Beyond this density, the MSE steadily increases and the AUC experiences a

relatively sharp drop towards 0.5. Note that while we still can perform reconstruction at higher densities, there is a trade-off between false negatives and false positives.

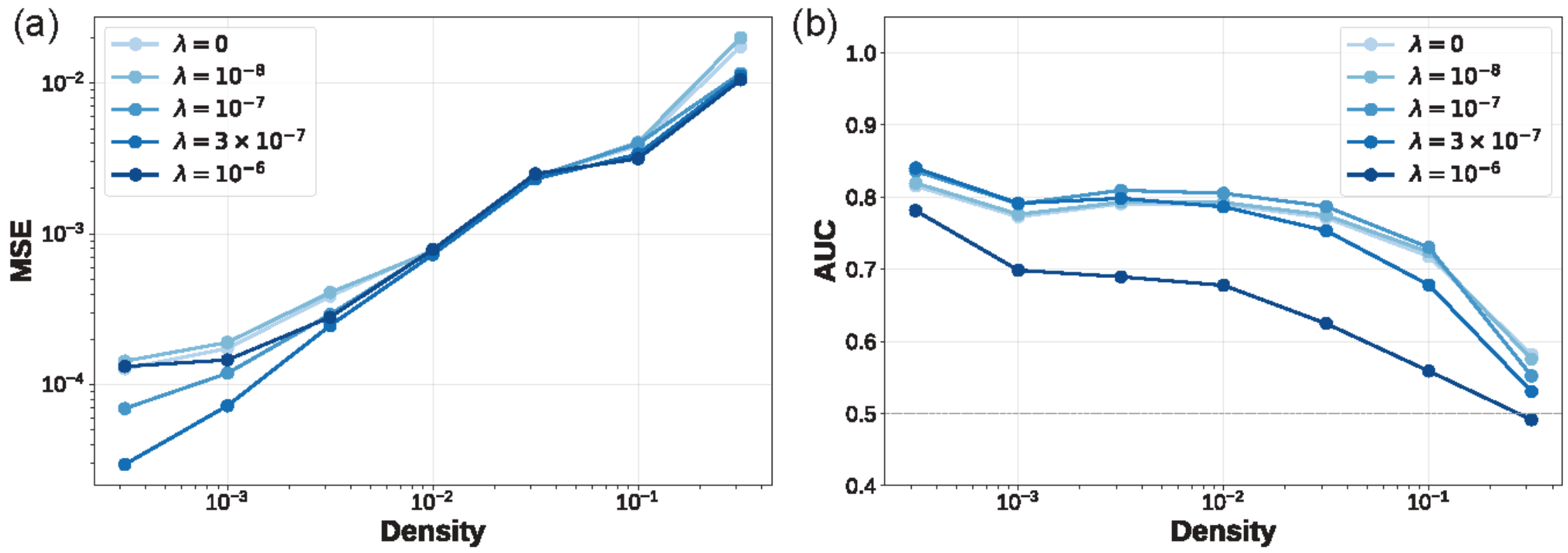

Fig. S7. Quantitative evaluation of reconstruction fidelity across varying target densities. (a) Mean squared error (MSE) between the reconstructed continuous intensities and the ground truth. (b) Area under the curve (AUC) for the pixel-wise classification of binary target dots. Performance is plotted across multiple L_1 regularization parameters (λ) to illustrate the optimization trade-offs. The dashed gray line at 0.5 represents the baseline performance of a random classifier.

Finally, we agree with the Reviewer that the pixels-per-second specification is contingent on satisfying the above sparsity constraints. From an information perspective, we can consider our sensor array's pixel rate of 4-5 GP/sec as a limit. Thus, we have qualified this the reconstructed throughput values as follows throughout the manuscript:

- Abstract: "culminating in an effective total spatiotemporal throughput of 25.2 billion pixels per second"
- Sec. 3.1 (System Characterization):
    - "for a total per-frame pixel count of 12621 x 16655 = 210 MPs and a total effective imaging throughput of up to ~25.2 GP/sec"
    - "Thus, our fluorescence imaging resolution is aberration-limited, for a total effective imaging throughput of up to ~2.17 GP/sec."

R2.2. The underlying assumptions of the Candes/Tao/Donoho compressive sensing theory are rarely satisfied in imaging systems, although they may be satisfied for images sparse on the natural basis. On the other hand, coding with a 15 lobe PSF in a shift invariant system definitely does not meet the random projection assumptions of CS theory. In view of this and many other performance issues, image reconstruction with TV/L1 optimization as implemented here is definitely not state of the art.
The paper would be greatly improved by using more appropriate estimation algorithms.
Indeed, we entirely agree with the Reviewer that our quasi-random 15-point PSF is not "optimal" for compressive sensing, and we admitted as much in our original manuscript that our "PSF [...] is not optimal by any metric" (Supp. Sec. S1.2, end of first paragraph). A better design of the A matrix, for example, would be one that minimizes pairwise correlation between the columns

(which is equivalent to minimizing the autocorrelation of a PSF of a shift-invariant, gap-free imaging system at non-zero shifts). However, in our situation, we run up against a few additional practical design constraints: 1) because this is an incoherent imaging system, the PSF (and therefore entries of A) must be nonnegative, 2) because we are photon-starved (e.g., weak fluorescence, short camera exposures), we need to minimize the number of pixels into which our PSF is distributed, which can be at odds with the random projections/low mutual coherence criteria of compressive sensing, and 3) chromatic aberrations induced by the DOE that worsen with PSF extent, inducing a tradeoff between signal level (i.e., due to necessary spectral filtering) and minimizing the PSF feature size. Given such practical constraints, among others, as the Reviewer points out, the underlying tenets of compressive sensing are rarely satisfied in imaging systems, ours included.

Due to constraint 1, which is common among all types of incoherent imaging systems, it is very hard to make pairwise column correlations of A close to 0 for all pairs for an extended, nonnegative PSF. For example, DiffuserCam [29], which uses a distributed, nonnegative, caustic PSF, exhibits a nearly uniform correlation of ~0.5 for any two non-identical field positions. In our case of the quasi-random 15-point PSF, if we first assume a gap-free sensor, the autocorrelation of the PSF is 0 at almost all spatial shifts except at a few shifts where the correlation is 1/15 = 0.067 – when one point overlaps in the PSFs (we have designed the pattern such that only one will overlap at once). In this sense, our system (if using gap-free sensor) has lower mutual coherence than DiffuserCam as well as systems with speckle PSFs. If we now consider our actual sensor array, with gaps, the column correlations increase, as the denominator is now < 15 (i.e., not all 15 points are detected) – in the worst case, only two PSF points are seen by a sensor and hence the correlation can reach 0.5 in a few places, matching that of caustic or speckle PSFs. This is not to say that such PSFs are necessarily ideal PSFs for compressive sensing, but rather that they have been successfully employed.

We can decrease the column correlations further by increasing the number of points, k, in our PSF beyond k=15. As long as their distribution is strategically placed such that at most one point overlaps at any two given field positions, the maximum correlation would be 1/k and hence can be made arbitrarily small, which is good for compressive sensing. Indeed, this strategy was loosely adopted in the Fourier DiffuserScope [27]. However, due to the aforementioned constraint 2 (low-light conditions), we cannot arbitrarily increase k because our signal would be drowned in read noise. Thus, we struck a balance between these two constraints in choosing k=15. For completeness, we note a third design constraint driven by pan-visibility (i.e., no matter the position in the FOV, the imaging system maps signal to at least one sensor); however, this constraint promotes larger k and hence is not at odds with compressive sensing.

So far in this discussion and in our paper, we have assumed sparsity in the native spatial domain, and thus to meet this assumption we restricted our samples to have relatively few *C. elegans*. However, other potential samples of interest may be sparse in other domains (e.g., gradient, wavelet, Fourier) as the Reviewer points out, in which case we would need to adopt a different PSF design strategy. This would be an interesting avenue for future investigation.

Some of these above considerations are discussed in Supplementary Sec. S1.1, but we do recognize, thanks to the Reviewer, that some of these discussions are missing. We have thus added the following paragraph to the Discussion section:

> “The PSF design for our compressive microscope also warrants further discussion. Here, we used a design heuristic based on a discrete distribution of impulses that maximizes the rank of the linear forward model, while balancing SNR considerations (Supplementary Sec. S1; Fig. S17). Our heuristic also reduces the column correlations of our linear model by breaking PSF symmetry so that the maximum PSF overlap between any two field positions is a single impulse. However, there are infinitely many PSFs that satisfy these criteria. Furthermore, such a design presupposes sparsity in the native space domain, which we enforced by ensuring sparse distributions of *C. elegans*. Thus, there is room for future investigation to further optimize the PSF design for more general samples based on sparsity in other domains or in a data-driven fashion. Such multiplexing PSF designs must be balanced with practical constraints, such as low photon counts typical of high-speed fluorescence imaging, nonnegativity of the PSF, and chromatic aberrations of larger PSFs.”

Reviewer #3 (Remarks to the Author):

The manuscript by Zhou et al., titled "Large-scale compressive microscopy via diffractive multiplexing across a sensor array," proposes a snapshot computational microscope that uses a 6×8 sensor array and a pupil-plane DOE to generate a non-symmetric 15-spot PSF. With 4×4 binning to reach 120 fps acquisition, the system reports darkfield reconstructions at ~2.76 μm resolution over 5.3 cm² and fluorescence at ~4.38 μm resolution over ~9.3×9.3 mm².

The engineering is excellent, and the advance is interesting.
We thank the Reviewer for their positive assessment!

R3.1. There are a few points that merit clarification. These points were not clear in the initial reading of the abstract, and it could be good to clarify or be more precise about the claims.

R3.1a. The paper emphasizes a ~210 MP/frame reconstructed output and ~25.2 GP/s equivalent throughput; however, these reflect the reconstruction grid rather than the raw sampling.
Yes, the Reviewer raised a very important point – we should be careful not to conflate data rate with reconstructed pixel rate, where the former is a more information-theoretic limit set by the camera streaming bandwidth (4-5 GB/sec). To minimize such confusions, we have now qualified the reconstructed pixel rate as the "effective throughput", in several places:

- Abstract: "culminating in an effective total spatiotemporal throughput of 25.2 billion pixels per second"
- Sec. 3.1 (System Characterization):
  - "for a total per-frame pixel count of 12621 x 16655 = 210 MPs and a total effective imaging throughput of up to ~25.2 GP/sec"
  - "Thus, our fluorescence imaging resolution is aberration-limited, for a total effective imaging throughput of up to ~2.17 GP/sec."

R3.1b. Reconstruction uses a fully shift-variant forward model with TV+L1 regularization and non-negativity, implemented via direct superposition;
Yes, we agree it would be useful to clarify these points – to this end, we have updated the abstract:

> "We then perform a large-scale regularized reconstruction using a direct fully shift-variant yet efficient convolutional forward model, assuming the object is sparse in some domain."

R3.1c. The DOE (64-level grayscale lithography) and PSF layout are chosen using a triple-correlation criterion. Demonstrations include static targets and C. elegans videos in darkfield and GCaMP fluorescence; fluorescence performance is good, yet non-uniform across the field, and the method currently operates offline rather than end-to-end in real time.
We thank the Reviewer for raising these points of clarification – we have updated the manuscript in a few places to include most of these points:

- End of the introduction: “To implement this PSF, we fabricated a 64-level diffractive optical element (DOE; Sec. 5.2) using grayscale lithography and placed it in the Fourier plane of a nearly 4f system.”
- End of the introduction: “Our PSF design is optimized to maximize the rank of the forward model, which we evaluated using a triple correlation between the PSF, itself, and the sensor array support (Eq. S2).”
- Discussion (paragraph 2): “As a result, our microscope contains aberrations and vignetting (see corners of Figs. 3c and 4c) that prevent diffraction-limited resolution across the entire FOV (Supplementary Figs. S12-S15).”

Regarding the Reviewer’s last point, we acknowledge that our method requires offline post-processing and is thus not real time. We believe this is not a significant limitation, as the high effective throughput of our system (210 MP/frame at 120 fps) exceeds the bandwidth of human visual processing (and that of most, if not all, modern display monitors) and is thus not meant to be consumed by a human observer in real time. Nevertheless, at <5 sec/frame, our processing speed is still quite fast for such a large-scale computational reconstruction, owing to JAX acceleration. As such, reconstructing crops of the full FOV would be proportionally faster – potentially in real time for human consumption.

Here are some questions and concerns:

R3.2. The C. elegans data is quite sparse, and this can aid in reconstruction. Can the authors demonstrate performance in dense samples and compare with ground truth data with more conventional imaging?

In this revision, we have added a suite of experimental results analyzing the effects of sparsity on reconstruction quality. We used a smartphone OLED display to generate randomly scattered dots at different densities. The exact digital image displayed on the screen serves as our ground truth after scaling based on the OLED pixel pitch.

We have added an experimental study in Supplementary Section S6 and Fig. S6 to discuss the sparsity limitations and the resulting trade-offs. As the Reviewer correctly intuited, sparsity aids reconstruction. For sufficiently sparse targets (density $d \leq 10^{-2}$), we achieve near-perfect, artifact-free recovery. For denser samples (density $d > 10^{-2}$), high-fidelity recovery becomes more challenging. While the system can still reconstruct these denser samples, there is an unavoidable trade-off between false negatives (missed target points) and false positives (reconstruction artifacts), which is modulated by the chosen L_1 regularization strength. This trend of degraded performance at higher densities is quantitatively tracked using mean squared error (MSE) and area under the curve (AUC) metrics across varying sample densities in Fig. S7.

- Sec. S6 (Impact of sample sparsity on reconstruction quality):

  To quantify the effect of sample sparsity on reconstruction fidelity, we conducted an empirical study using a programmable target. Sparsity is quantified by the sample density, *d*, defined as the fraction of non-zero pixels across the object field-of-view (FOV). We utilized the darkfield imaging configuration, which consists of an FJW Industries objective (*f* = 90 mm), a 514/2 nm narrowband filter, and a 40-mm-diameter

aperture (though note that we did not perform darkfield illumination). To provide a controllable ground truth (GT) across the full FOV, we employed an iPhone 17 Pro screen as the imaging target, generating synthetic patterns consisting of randomly scattered green dots (corresponding to ~20 µm OLED pixels).

The qualitative results, illustrated in Fig. S6, reveal an empirical threshold for successful image recovery. At a high density ($d > 10^{-2}$), the system is unable to achieve a high-fidelity reconstruction. Regardless of the ℓ_1 regularization weight (λ) applied, the algorithm either fails to reconstruct certain points (false negatives, appearing as red in the overlay) or introduces artifacts (false positives, appearing as green). Conversely, when the sample is sufficiently sparse ($d \sim 10^{-2}$ or lower), artifact-free recovery is readily achieved using an optimal sparsity regularization weight. In these highly sparse regimes, the reconstruction and ground truth are nearly perfectly coincident, appearing as white.

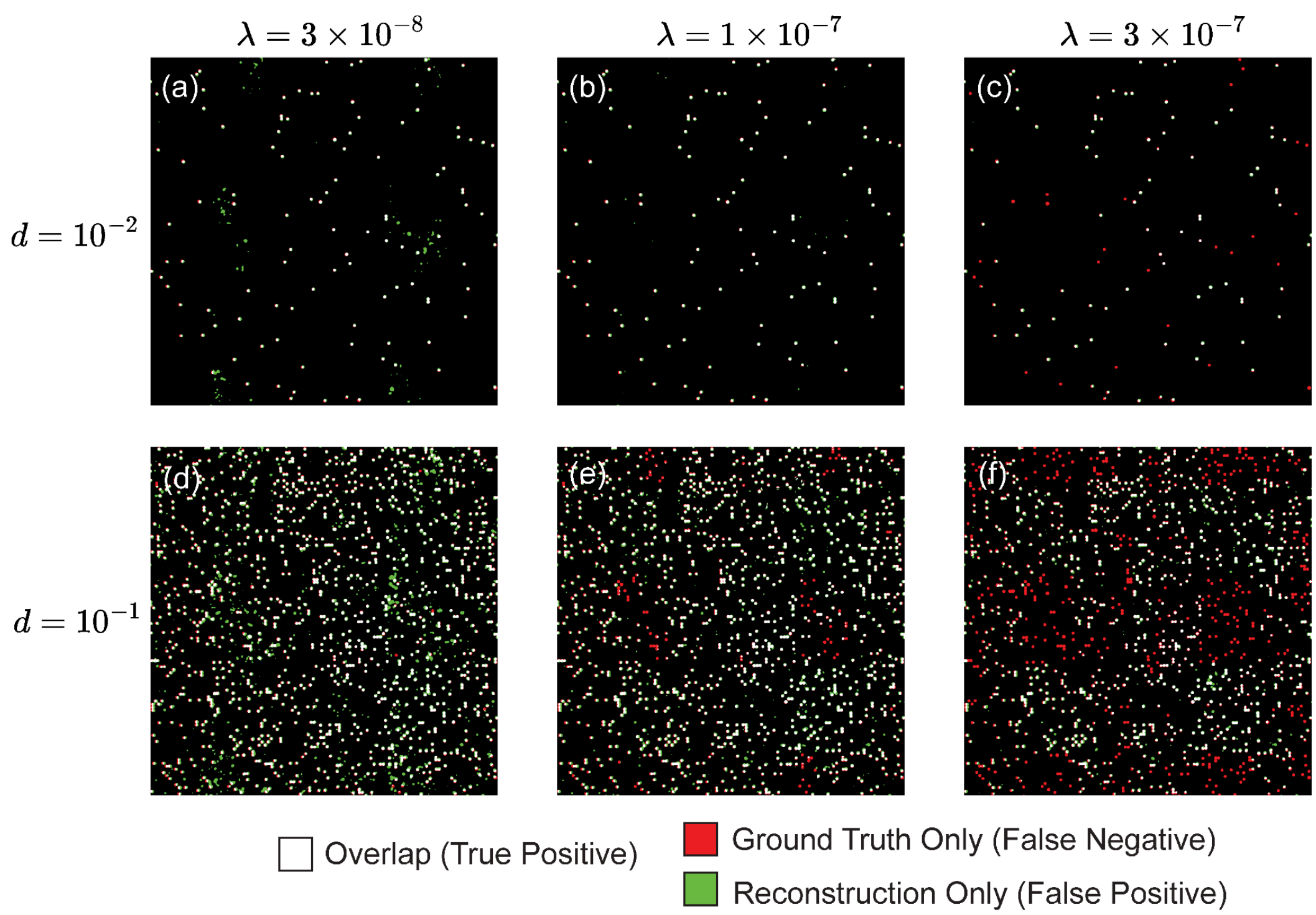

Fig. S6. Experimental evaluation of reconstruction fidelity versus object density. Binarized overlays (threshold 0.1) comparing reconstructions across varying L_1 regularization strengths (λ). White indicates accurate recovery (true positive), red indicates missed ground truth (false negative), and green indicates artifacts (false positive). (a-c) Reconstructions at a lower object density ($d=10^{-2}$), showing near-perfect overlap at the optimal λ. (d-f) Reconstructions at a higher object density ($d=10^{-1}$), exhibiting increased false positives and false negatives across all evaluated λ values. All panels display a representative ~6.3 x 6.3 mm^2 physical region cropped from the center of the full multi-centimeter field-of-view.

To benchmark our reconstruction performance across varying target densities, we quantitatively evaluated both the continuous pixel intensities and the binary recovery of the dots displayed on the screen in Fig. S7. Prior to computing performance metrics, both the reconstructed images and the ground truth were downsampled by 8× for computational efficiency and smoothed with a Gaussian kernel (σ = 10 pixels). This blurring step accommodates small residual distortion, ensuring the evaluation focuses on successful target recovery. First, we measured the mean squared error (MSE) between these preprocessed images to quantify overall intensity accuracy. Second, we treated the dot recovery as a pixel-wise classification problem. By applying varying intensity thresholds, we generated receiver operating characteristic (ROC) curves and calculated the area under the curve (AUC) for each density level. An AUC of 1.0 indicates a perfect reconstruction, while 0.5 represents a random guess. As demonstrated across multiple regularization weights (λ), reconstruction fidelity remains high for target densities below 10^{-2}. Beyond this density, the MSE steadily increases and the AUC experiences a relatively sharp drop towards 0.5. Note that while we still can perform reconstruction at higher densities, there is a trade-off between false negatives and false positives.

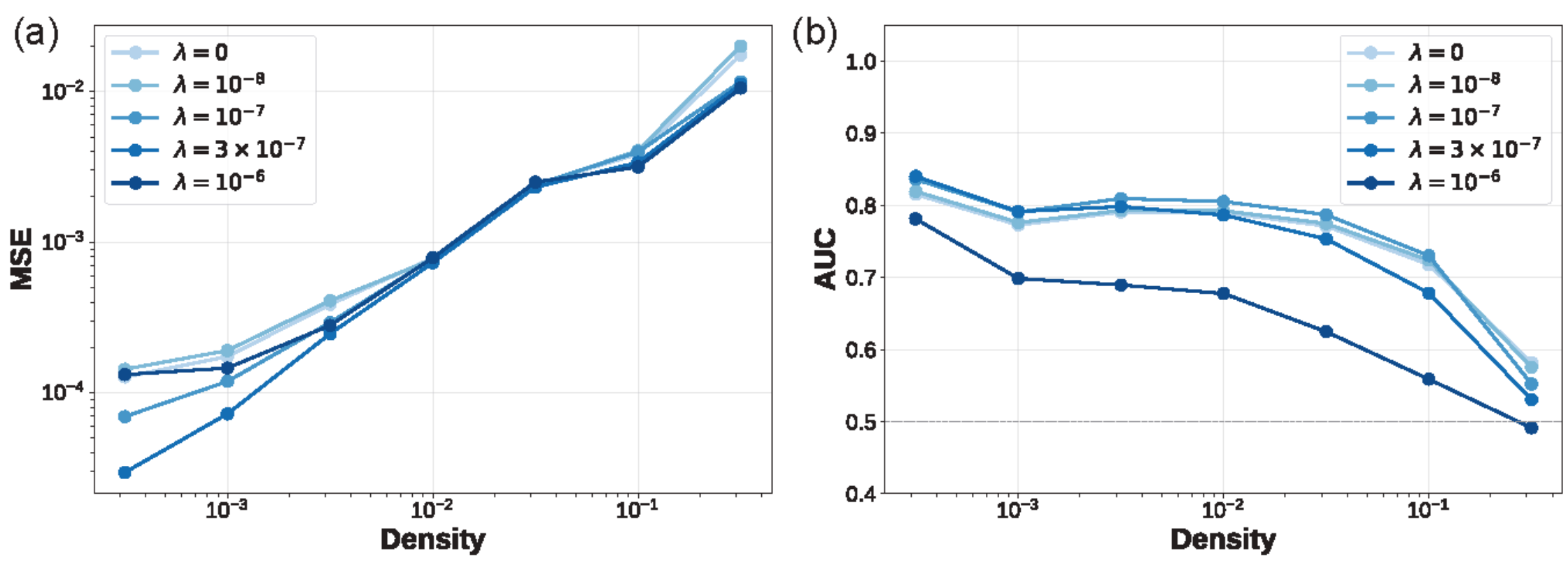

Fig. S7. Quantitative evaluation of reconstruction fidelity across varying target densities. (a) Mean squared error (MSE) between the reconstructed continuous intensities and the ground truth. (b) Area under the curve (AUC) for the pixel-wise classification of binary target dots. Performance is plotted across multiple L_1 regularization parameters (λ) to illustrate the optimization trade-offs. The dashed gray line at 0.5 represents the baseline performance of a random classifier.

R3.3. Generally, can the authors show when performance breaks down? What are the limits?
We appreciate the reviewer's follow-up question regarding the operational limits of our method. Defining a strict point where the system "breaks down" is somewhat nuanced, as the reconstruction error actually increases steadily as the object becomes denser, rather than experiencing a sudden, catastrophic failure. However, in terms of practical limits for high-fidelity imaging, our empirical analysis reveals a performance threshold at a sample density of roughly $d \sim 10^{-2}$. Beyond this density limit, the area under the curve (AUC) for target recovery drops sharply and the system struggles to balance false positives and false negatives regardless of the chosen L_1 regularization strength. We have incorporated this discussion into Supplementary

Section S6 and directed readers to the quantitative performance curves in Fig. S7 to illustrate exactly how and where this gradual degradation occurs (see above in R3.2).

R3.4. The paper did not explicitly report the raw pixel count per frame after 4x4 binning. It should be around 38.5 MP. The authors should differentiate the physical sampling throughput and reconstructed output pixels. They should be clear about the claims so that readers do not get confused about the performance.
In addition to the changes we made in response to your previous comment (R3.1a), where we have qualified reconstructed throughput figures with “effective”, we have also updated Sec. 2.1 to clarify that the 617 MP/frame refers to the un-downsampled pixel count while 38.5 MP/frame refers to the 4x4 downsampled count:

> “The “super sensor” consists of a 6×8 array of 48 8-bit monochrome sensors (3136×4096 pixels, 1.1-μm pixel pitch, 9-mm center-to-center spacing) for a total of 0.617 gigapixels (GPs) per snapshot (without downsampling), whose rectangular hull covers an area of 4.95 cm × 6.64 cm. The sensor array frame rate has two bottlenecks: the data transfer bandwidth of 6.17 GB/sec and the sensor readout rate. At full 0.617 GP sampling, the frame rate is bandwidth-limited to 10 fps, which in practice is too slow for imaging freely moving C. elegans at high resolution. To achieve higher frame rates, we use 4×4 pixel binning (38.5 MP per snapshot) to achieve 120 fps (readout-limited).”

R3.5. The PSF design of the DOE element is done through computer simulation and triple correlation. It would be nice if the author could provide more information on this.
a. More simulation results on DOE fabrication
b. Monte Carlo tolerance analysis
c. Maybe detail the metrology process
We have added a new figure (Fig. S16), whose panel c shows representative metrological characterization of the height profile of the DOE using a commercial confocal microscope (Olympus LEXT OLS5000):

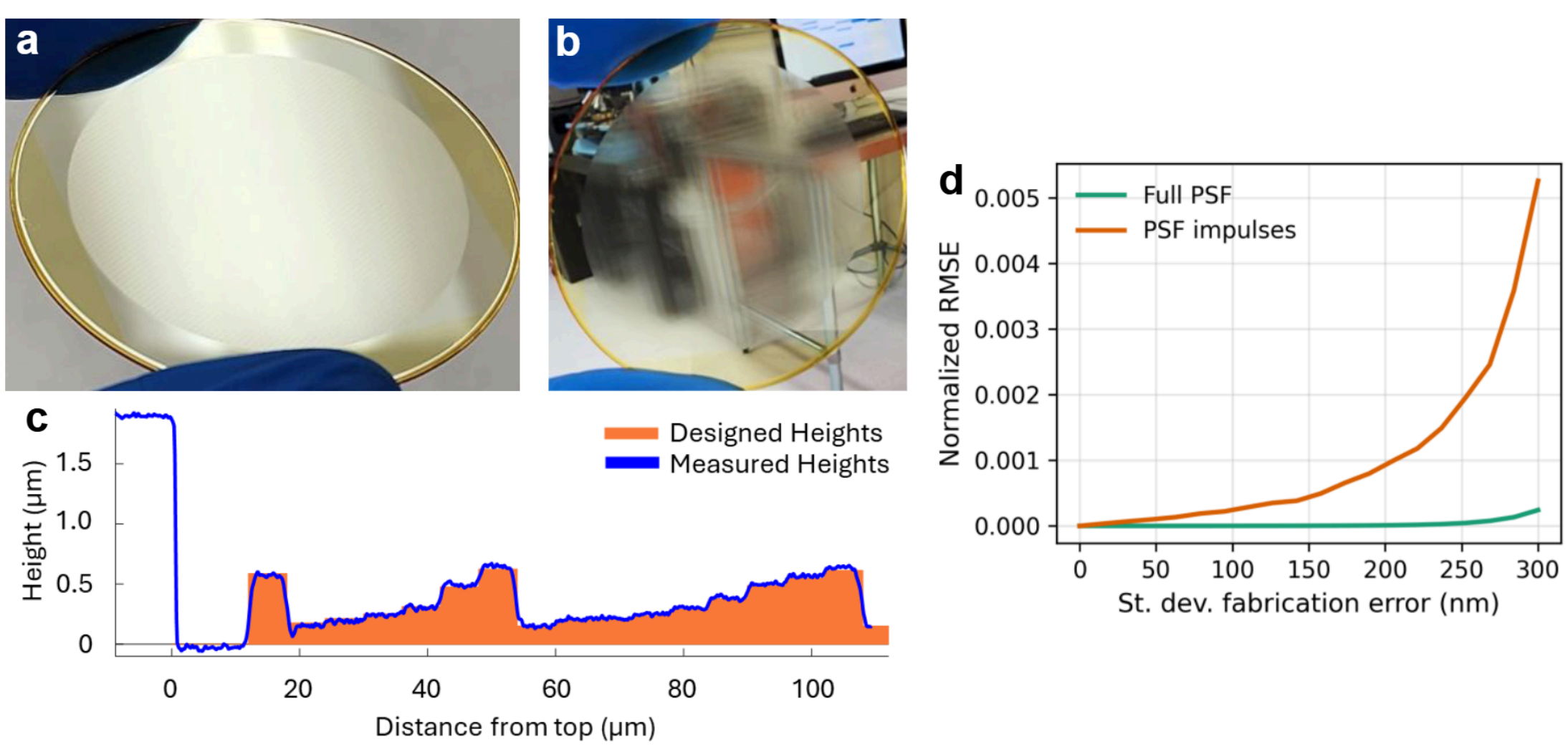

Fig. S16. (a-b) Photographs of the DOE used in our microscope. (b) illustrates the multiplexing effect of the DOE, superimposing multiple copies of the object/scene.

(c) Characterization of the height profile of the DOE near edge of the pattern, using a confocal microscope (Olympus LEXT OLS5000), showing close agreement with the target design. (d) Sensitivity of PSF to DOE fabrication error (Gaussian noise with various standard deviations). We computed the root mean square error (RMSE) to the nominal PSF, normalized to the peak intensity of the nominal PSF. Two separate RMSE values were computed, one for the whole PSF, another for small window surrounding each of the 15 impulses. Each curve is an average of n=20 random noise realizations.

We also performed new simulations to assess tolerance of the PSF intensity distribution to DOE fabrication errors (panel d above). In particular, we added Gaussian noise with varying standard deviations to the design height distribution of the DOE, computed the Fourier transform to obtain the intensity PSF. From there, we computed the root mean square error (RMSE) to the nominal PSF, normalized to the peak intensity of the nominal PSF. We computed two such RMSE values, one across the entire PSF, another across a small window around each of the 15 impulses. These two curves are based on an average of 20 random noise realizations. Since our DOE fabrication error standard deviation is ~30 nm (which may be an overestimate, due to the measurement uncertainty of our confocal microscope), the impact on the PSF accuracy is negligible.

The text that references this figure is from Sec. 5.2:

> "A confocal microscope (Olympus LEXT OLS5000) was used to measure the heights of several outermost pixels in various locations on the sample (Fig. S16c). The average height difference between fabrication and ideal design was approximately 20 nm, with a standard deviation of 30 nm, which would have negligible impact to the accuracy of the PSF (Fig. S16d)."

R3.6. The authors could include the synchronization process of all the imaging sensors (global or rolling shutter). Would the assembly tip/tilt affect image reconstruction?

We have added the following text near the end of paragraph 1 of Sec. 2, regarding synchronization:

> "The 48 sensors all have the same rolling shutter readout pattern, synchronized to within 10 μs, limited by the I2C serial communication protocol [19]."

Regarding your second question about tip/tilt, yes, this was a very important consideration due to our extremely wide FOV compared to the DOF. We addressed this issue entirely in the hardware by placing the sample on a large tip-tilt stage to align the flat sample with the focal plane of our microscope. We added the following sentence near the beginning of the Results section (Sec. 3):

> "The sample was placed on a tip-tilt stage to ensure that it was flat across the entire FOV."

R3.7. The authors claim that the FOV could be expanded to 6-7 mm. The author could quantify the extrapolation boundary by showing a map with number of PSF at each location. The quality of the PSF would degrade at the edge, which might not be suitable for imaging.

Yes, in practice the extrapolation boundary is relatively small compared to the boundary enclosing the sensor array, as we are only extrapolating by half of the PSF extent beyond all four boundaries. Fig. S3 experimentally demonstrates the feasibility of such an extrapolation:

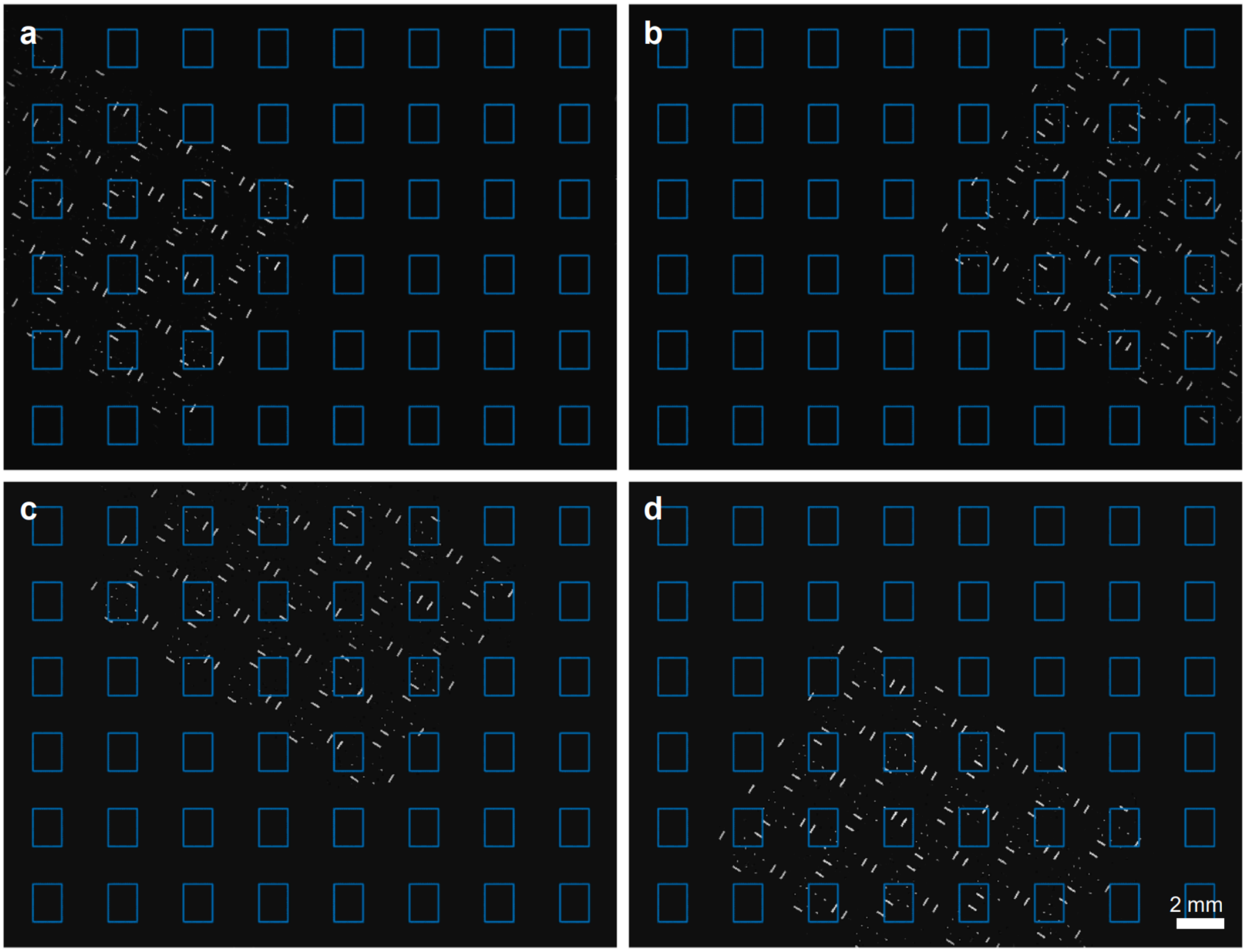

Here, the blue rectangles represent the coverage of the sensor array and the intervening regions represent the computationally “filled in” regions, including the marginal borders. The same binary lithographic mask sample is imaged in all four panels at different positions straddling the four boundaries of the FOV, indicating that we obtain accurate reconstructions in the extrapolated margins.

R3.8. 8-bit readout is a low dynamic range. The signal might saturate when having a high fluorescence signals and/or weak signals can get lost in the noise. The authors could provide more information on the characteristics of intensity histograms. Showing cases where the quality of the reconstructed image is poor could help the reader understand the limits.
The Reviewer raises an excellent point – indeed, the limited 8-bit dynamic range of our sensors could pose problems. In fact, if we plot the histograms of both the darkfield and fluorescence *C. elegans* videos, we can see that we don’t even use all 255 gray levels in this result, so that it’s effectively 6.5-7-bit. We compiled the detected photoelectron counts across all frames of the video for a central sensor, as plotted in the histogram below.

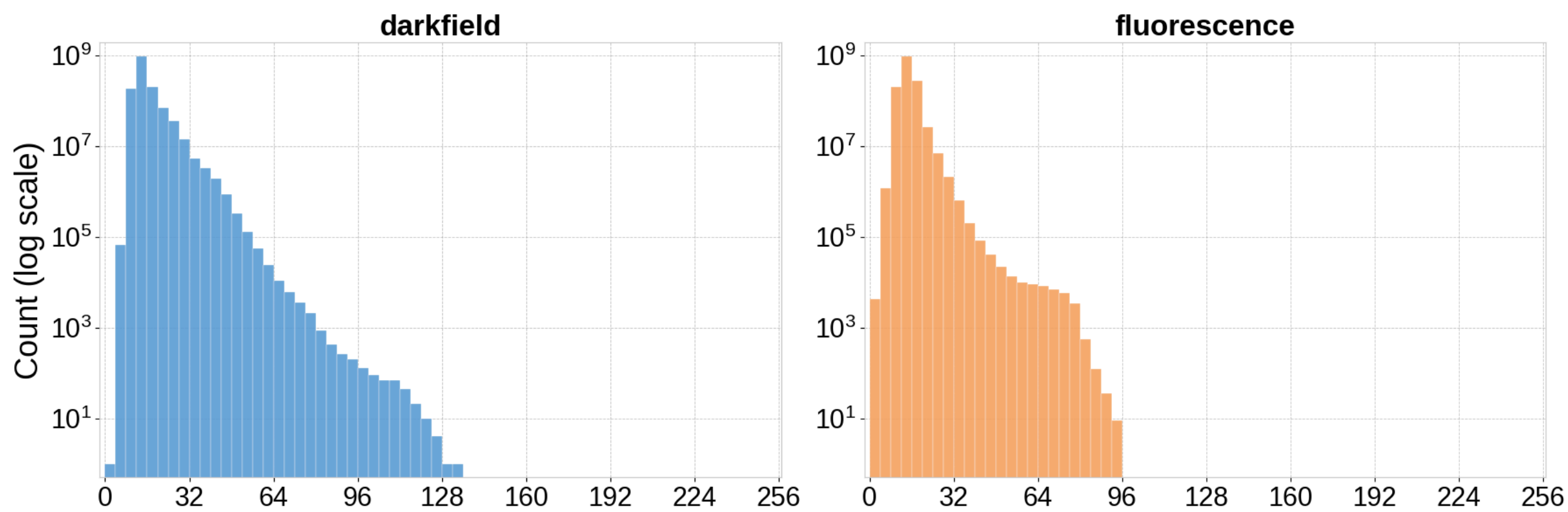

This is particularly relevant in our situation due to 1) intrinsically weak fluorescence/darkfield signals, due to our high frame rates, and 2) multiplexing, further splitting the weak signals into multiple pixels and introducing overlap. These considerations also interact with our sparsity-promoting regularizers, which may lead to false negative reconstructions. This places a lower limit on how bright the samples we're imaging need to be, as the signal needs to be above the discretization noise floor after being distributed into 15 points. However, in practice, it is difficult to distinguish the effects of low bit depth and sparsity-regularized reconstruction, as they both tend to erase/underestimate weak signals.

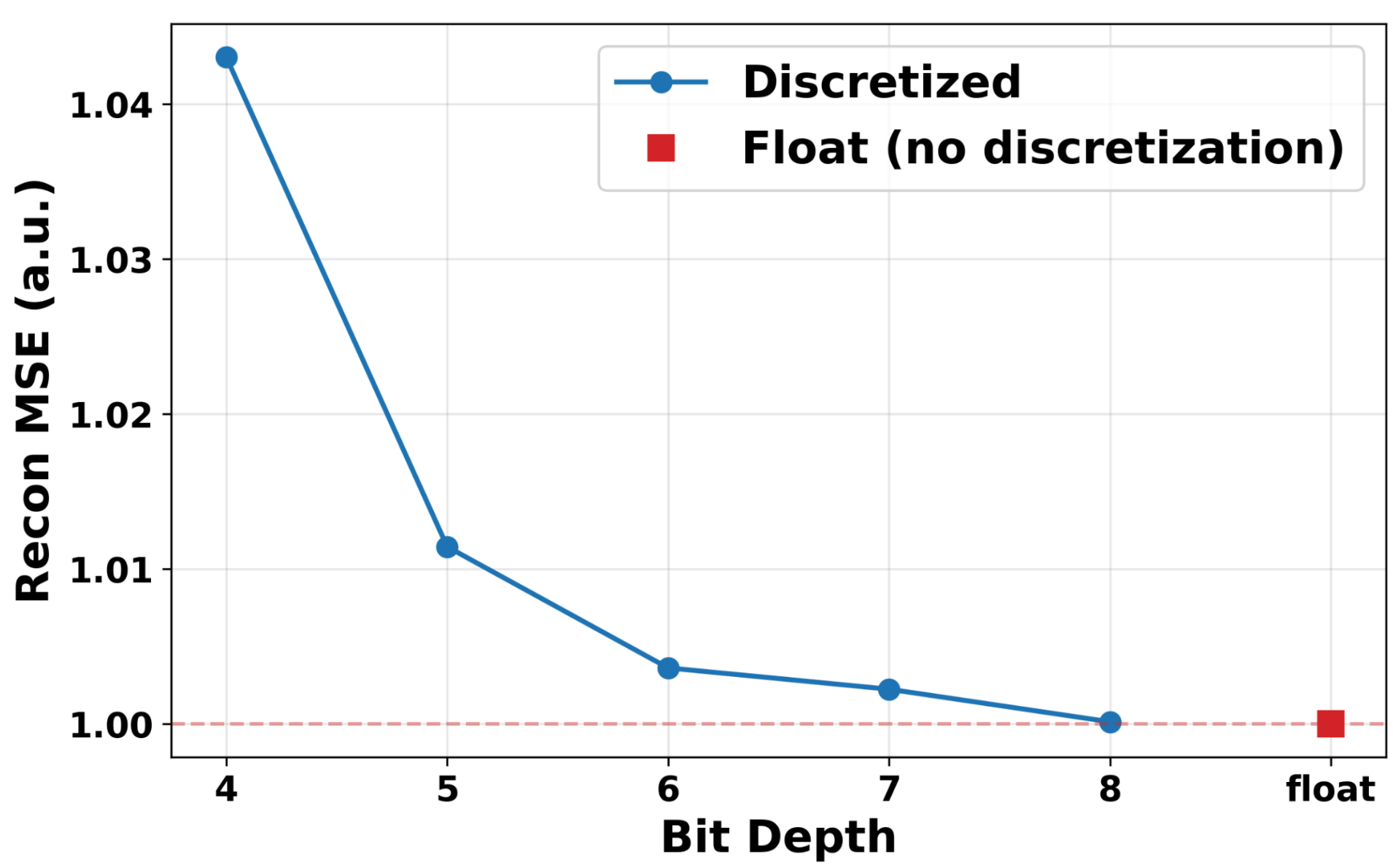

This figure shows the impact of bit depth on reconstruction accuracy. Simulated measurements were generated in a shot-noise limited regime with a peak per-pixel count of 312 photoelectrons, approximately matching the sensor's effective saturation capacity at the experimental analog and digital gain settings. These continuous signals were then quantized to bit depths ranging from 4 to 8 bits prior to image reconstruction. The reconstruction mean

squared error (MSE), normalized to the unquantized baseline, demonstrates that the 8-bit reconstruction error converges to the unquantized limit, so theoretically 8-bit digitization introduces negligible quantization error and does not cause significant loss of weak signals beyond the shot-noise limit. In fact, lower bit depths are also acceptable.
